# Microglial derived extracellular vesicles activate autophagy and mediate multi‐target signaling to maintain cellular homeostasis

**DOI:** 10.1002/jev2.12022

**Published:** 2020-11-25

**Authors:** Bram Van den Broek, Isabel Pintelon, Ibrahim Hamad, Sofie Kessels, Mansour Haidar, Niels Hellings, Jerome J.A. Hendriks, Markus Kleinewietfeld, Bert Brône, Vincent Timmerman, Jean‐Pierre Timmermans, Veerle Somers, Luc Michiels, Joy Irobi

**Affiliations:** ^1^ Biomedical Research Institute UHasselt Hasselt University Hasselt Belgium; ^2^ Laboratory of Cell Biology & Histology Antwerp Centre for Advanced Microscopy (ACAM) University of Antwerp Antwerp Belgium; ^3^ VIB Laboratory of Translational Immunomodulation VIB Center for Inflammation Research Hasselt Belgium; ^4^ Peripheral Neuropathy Research Group Department of Biomedical Sciences Institute Born Bunge and University of Antwerp Antwerp Belgium

**Keywords:** autophagy, cellular homeostasis, extracellular vesicles, gene expression, immunity, inflammation genes, microglia, RNA sequencing

## Abstract

Microglia, the immunocompetent cells of the central nervous system (CNS), play an important role in maintaining cellular homeostasis in the CNS. These cells secrete immunomodulatory factors including nanovesicles and participate in the removal of cellular debris by phagocytosis or autophagy. Accumulating evidence indicates that specifically the cellular exchange of small extracellular vesicles (EVs), participates in physiology and disease through intercellular communication. However, the contribution of microglial‐derived extracellular vesicles (M‐EVs) to the maintenance of microglia homeostasis and how M‐EVs could influence the phenotype and gene function of other microglia subtypes is unclear. In addition, knowledge of canonical signalling pathways of inflammation and immunity gene expression patterns in human microglia exposed to M‐EVs is limited. Here, we analysed the effects of M‐EVs produced in vitro by either tumour necrosis factor alpha (TNFα) activated or non‐activated microglia BV2 cells. We showed that M‐EVs are internalized by both mouse and human C20 microglia cells and that the uptake of M‐EVs in microglia induced autophagic vesicles at various stages of degradation including autophagosomes and autolysosomes. Consistently, stimulation of microglia with M‐EVs increased the protein expression of the autophagy marker, microtubule‐associated proteins 1A/1B light chain 3B isoform II (LC3B‐II), and promoted autophagic flux in live cells. To elucidate the biological activities occurring at the transcriptional level in C20 microglia stimulated with M‐EVs, the gene expression profiles, potential upstream regulators, and enrichment pathways were characterized using targeted RNA sequencing. Inflammation and immunity transcriptome gene panel sequencing of both activated and normal microglia stimulated with M‐EVs showed involvement of several canonical pathways and reduced expression of key genes involved in neuroinflammation, inflammasome and apoptosis signalling pathways compared to control cells. In this study, we provide the perspective that a beneficial activity of in vitro cell culture produced EVs could be the modulation of autophagy during cellular stress. Therefore, we use a monoculture system to study microglia‐microglia crosstalk which is important in the prevention and propagation of inflammation in the brain. We demonstrate that in vitro produced microglial EVs are able to influence multiple biological pathways and promote activation of autophagy in order to maintain microglia survival and homeostasis.

## 1 INTRODUCTION

The central nervous system (CNS) is a multifaceted structure in which cell‐to‐cell communication is vital to maintain homeostasis. Glial cells are involved in maintaining CNS functions including neuronal development and repair (Domingues, Portugal, Socodato, & Relvas, 2016). Furthermore, crosstalk between glia and neurons is vital for a range of biological functions and to maintain protein homeostasis within the CNS. However, these proteostasis networks decline with age and during persistent neuroinflammation, facilitating neurodegenerative diseases (Sweeney et al., 2017). Microglia are the resident immune cells of the CNS, in which each microglia subtype displays intrinsic properties and performs unique functions important for maintaining CNS homeostasis (Bialas et al., 2017; Lenz & Nelson, 2018; Paolicelli et al., 2017; Stratoulias, Venero, Tremblay, & Joseph, 2019; Yin, Valin, Dixon, & Leavenworth, 2017). In addition, being the mediators of neuroinflammation, microglia secreted factors have been ascribed various physiological roles, for example, providing trophic support to neurons and clearance of myelin debris (Graeber, Li, & Rodriguez, 2011; Paolicelli, Bergamini, & Rajendran, 2019; Smolders et al., 2019). As the primary source of inflammatory mediators in the CNS, microglia play a key role in immune regulation within the brain by the production of immunological factors including chemokines, cytokines, reactive oxygen species and extracellular vesicles (Colonna & Butovsky, 2017; Li & Barres, 2018; Miyamoto et al., 2016; Paolicelli et al., 2019; Parkhurst et al., 2013; Smith, Das, Ray, & Banik, 2012; Wang, Tan, Yu, & Tan, 2015; Wolf, Boddeke, & Kettenmann, 2017). Recent reports indicate that extracellular vesicles (EVs) play a crucial role in delivering trophic support to maintain the glia‐neuron crosstalk (Frühbeis, Fröhlich, Kuo, & Krämer‐Albers, 2013). Glia and neurons can communicate by releasing and receiving EVs, which allows an organized communication across short and long distances (Budnik, Ruiz‐Cañada, & Wendler, 2016; Galluzzi, Yamazaki, & Kroemer, 2018; Kramer‐Albers & Hill, 2016; Lai & Breakefield, 2012; Rajendran et al., 2014). EVs are nanosized membrane vesicles that are released by almost every cell type (Paolicelli et al., 2019). Depending on their size and origin, EVs can be subdivided into exosomes, microvesicles (MVs), and apoptotic bodies, and are released by either healthy or diseased cells (Colombo, Raposo, & Théry, 2014; György et al., 2011; Théry, Ostrowski, & Segura, 2009). A recent report has shown the detrimental and protective action of microglial EVs (M‐EVs) on myelin lesions (Lombardi et al., 2019), suggesting that M‐EVs could regulate biological processes in different cell types.

EVs are involved in intercellular signalling under either pathological or physiological conditions and deliver a complex cargo of proteins, lipids and nucleic acids to recipient cells (Abels & Breakefield, 2016). EVs from diverse origins are described to target microglia (Bahrini, Song, Diez, & Hanayama, 2015; Bialas et al., 2017; Fauré et al., 2006; Hong et al., 2016; Lui et al., 2016, Paolicelli et al., 2017). Recent studies have reported the critical role of EVs originating from neurons in regulating microglial activity, by upregulating the complement molecule C3 to facilitate the removal of degenerating neurites by microglia autophagy (Bahrini et al., 2015; Fauré et al., 2006; Jin et al., 2018). Autophagy is important, not only in preserving the source of nutrients necessary for cell survival, but also as a regulator of cytoplasmic quality control (Jin et al., 2018, Levine & Kroemer, 2008). Dysregulated autophagy, either excessive or impaired, has been linked to neurodegeneration and plays a crucial role in modulating neuroinflammation (Jin et al., 2018).

Cell type‐specific EVs are transiting towards clinical applications and the exchange of small EVs has been proposed to be one of the mediators of the therapeutic activities of mesenchymal stromal cells (Lener et al., 2015). EVs prepared from in vitro cell cultures have been reported to exhibit therapeutic activities in many pre‐clinical models of immunological and degenerative diseases (Moll et al., 2019; Witwer et al., 2019; Xu & Yang, 2019). Here, we provide the perspective that a beneficial activity of in vitro cell culture produced M‐EVs could be the modulation of autophagy during cellular stress. We determine to what extent proinflammatory microglia can influence the phenotype and gene function of other microglia via EVs. Therefore, a monoculture system is used to study microglia–microglia crosstalk which is important in the prevention and propagation of inflammation in the brain.

The activity of in vitro produced M‐EVs on activated human microglial and the mechanism by which these M‐EVs modulate neuroinflammation gene networks are not known. Importantly, the knowledge of M‐EVs impact on the human immunity and inflammation transcription profiles and how in vitro produced M‐EVs could modulate biological pathways in activated microglia is limited. Therefore, we hypothesize that in a monoculture system; microglia derived‐EVs modulate cellular stress response by activating autophagy and cell survival pathways.

In this study, we produced two subsets of microglia‐derived EV (M‐EVs). We denoted M‐EVs produced in non‐activated microglia as normal EVs (nEVs), and M‐EVs produced in microglia that were activated by TNFα to trigger cellular stress as activated EVs (aEVs). We investigated the effects of in vitro produced nEVs and aEVs on mouse BV2, human C20 and primary microglia cells, and examined how autophagy, cell survival, inflammation and immunity transcriptome profiles are modified.

## 2 MATERIAL AND METHODS

### 2.1 Cell culture material and conditions

The human microglia C20 cell line was kindly donated by Dr. David Alvarez‐Carbonell, Case Western Reserve University Cleveland, Ohio. The cells were grown at 37°C and 5% CO_2_ in Dulbecco's Modified Eagle's Medium (DMEM, Sigma‐Aldrich, D6429), supplemented with 1× N‐2 supplement‐A (Gibco‐Invitrogen, 17502‐048), 1 μM dexamethasone (PromoKine, PK‐CA577‐1042‐1), 1× penicillin streptomycin (Sigma‐Aldrich, P4333), and 10% fetal bovine serum (FBS, Biowest, S181B). Mouse microglia BV2 cell line was cultured at 37°C and 5% CO_2_ in complete medium, containing DMEM (Sigma‐Aldrich, D6429) supplemented with 1× penicillin streptomycin (Sigma‐Aldrich, P4333), and 10% FBS (Biowest, S181B).

### 2.2 Animals

Wild‐type C57BL/6J mice were mated to isolate primary microglia from postnatal day 3 (P3) pups. Mice were maintained in the animal facility of the Biomedical Research Institute (BIOMED) at Hasselt University in accordance with the guidelines of the Belgian Law and the European Council Directive. The animals were housed in standard cages on a 12 h light/dark schedule with a controlled room temperature of 22°C. Food and water were provided ad libitum. Experiments were conducted in accordance with the European Community guiding principles on the care and use of animals and with the approval of the Ethical Committee on Animal Research of Hasselt University.

### 2.3 Isolation and culturing of primary microglia

Primary microglia were isolated from P3 wild‐type mouse pups using the shake‐off method. Briefly, pups were decapitated and the cerebra were acutely isolated and transferred to ice‐cold 1× Hank's balanced salt solution (HBSS; Gibco, 14175‐053) supplemented with 7 mM HEPES (Gibco, 15630‐049), 100 U/ml penicillin, and 100 μg/ml streptomycin (1% P/S; Sigma‐Aldrich, P4333). The meninges were carefully removed using a stereomicroscope. The hemispheres were transferred to ice‐cold high glucose Dulbecco's Modified Eagle Medium (DMEM, Sigma‐Aldrich, D5796) containing 1% P/S and were mechanically homogenized and sieved through a 70 μm cell strainer. After centrifugation (300 × *g*, 10 min, 4°C), the pellet was re‐suspended in DMEM D5796 supplemented with 10% fetal bovine serum (Gibco, 26140‐079), 10% horse serum (Gibco, 26050–088) and 1% P/S. Mixed glial cultures were obtained by seeding the cell suspension in poly‐d‐lysine pre‐coated (PDL; 20 μg/ml, 1 h coating at 37°C, Sigma‐Aldrich, P6407‐5MG) T175 culture flasks for 7 days without changing the medium. Subsequently, the mixed glial cultures were incubated for 7 days in culture medium supplemented with 30% L929 conditioned medium containing macrophage colony‐stimulating factor (M‐CSF). The microglia‐enriched cultures were thoroughly agitated on a pre‐warmed orbital shaker (3 h, 230 rpm, 37°C) to collect detached microglia and seed them in PDL‐coated plates at cell densities appropriate for each assay. After 30 min of incubation, the medium was replaced to eliminate non‐adherent cells, hence increasing the microglia purity. Generally, this method provides a >95% pure microglia yield (Mecha et al., 2011). Magnetic activated cell sorting (MACS) was used to isolate primary microglia from P3 wild‐type mouse pups, hence reducing the time that microglia were maintained in culture. Upon removal of the meninges, the brains were mechanically dissociated and enzymatically digested with Papain and DNaseI. Mononuclear cells were isolated using a 30%‐70% Percoll gradient. MACS was performed according to the manufacturer's guidelines (MACS, Miltenyi Biotec) using MS columns (130‐042‐201) and CD11b microbeads (130‐049‐601). Microglia were seeded in PDL‐coated plates at cell densities appropriate for each assay. Microglia phenotyping was performed using RT‐PCR for microglial genes (HexB, Cx3cr1, Iba1, and CD11b) and non‐microglial genes (NeuN, GFAP) (Figure S1A). Primer sequences can be found in Table S5. Additionally, Iba1/GFAP immunocytochemistry was performed to determine microglia purity (Figure S1B).

### 2.4 Isolation of Microglial EVs from cell cultures

Microglia BV2 cells were seeded in a 175 cm^2^ Cellstar cell culture flask (Greiner bio‐one) and cultured at 37°C and 5% CO_2_ in complete growth medium, containing DMEM (Sigma‐Aldrich, D6429) supplemented with 1× penicillin streptomycin (Sigma‐Aldrich, P4333), and 10% FBS (Biowest, S181B). At 60% confluence, cells were washed with PBS (Lonza, 17–516F) and moved to reduced medium containing DMEM (Sigma‐Aldrich D6429), supplemented with 1× penicillin streptomycin (Sigma‐Aldrich, P4333) and 5% exosome depleted FBS (Gibco, A27208‐01). To activate microglia, cells were exposed to 10 ng/ml recombinant human TNFα (Immunotools, 11343015) for 24 h. To measure proliferation rate, cells were trypsinized and stained with trypan blue to determine cell count and viability using the EVE automatic cell counter (NanoEnTek). Isolation and characterization of M‐EVs was performed according to MISEV 2018 guidelines (Théry et al., 2018). EVs were either isolated from supernatant of non‐activated or TNFα‐activated microglia using differential centrifugation. Briefly, supernatant was collected and centrifuged at 300 × *g* for 10 min to remove living cells, at 2000 × *g* for 10 min to remove dead cells and debris, and at 10,000 × *g* for 30 min to remove large microvesicles. Supernatants were then transferred and ultra‐centrifuged at 115,000 × *g* for 3 h to pellet EVs. All centrifugation steps were performed at 4°C using an Eppendorf 5804 R centrifuge or a Beckman Optima XPN‐80 ultracentrifuge equipped with a Ti70 rotor. EV pellets were washed and resuspended in PBS, aliquoted and stored at ‐80°C. Size‐exclusion chromatography (SEC) was performed using chromatography columns (Bio‐Rad Laboratories, 732–1010) containing 10 ml sepharose CL‐2B beads (60–200 μm) (GE Healthcare, 17‐0140‐01). The columns were sterilized with 70% ethanol (VWR chemicals, 84857.360) and washed twice with PBS. One millilitre volume of differential ultra‐centrifuged EVs concentrate was loaded on the SEC column, followed by elution with PBS buffer to further purify the EVs. The eluate was collected immediately after EVs loading in 10 sequential fractions of 1 ml flow‐through. Based on Nanoparticle Tracking Analysis, collection volumes 4, 5 and 6 contained the highest EV concentration (data not shown). However, Böing et al. reported the presence of protein contaminants co‐eluting from collection volume fraction 6 (Böing et al., 2014). Therefore, fraction 6 was discarded, and up‐concentration was further performed for fractions 4 and 5 using Amicon 10k filters (Merck Millipore, UFC201024).

### 2.5 Quantification of Microglial EVs

Nanoparticle Tracking Analysis (NTA) was used to analyse the size distribution and concentration of BV2‐derived EVs based on the tracking of moving EV light scatter under Brownian motion using the NanoSight NS300 system (Malvern Panalytical) equipped with a 532‐nm laser. Briefly, samples were diluted in PBS (Lonza, 17–516F) over a concentration range between 10 and 100 particles per frame. All settings were manually set at the beginning of the measurements and kept constant during all acquisitions: camera level, 14; camera gain, 1; pump rate, 80; viscosity, 1. For each sample, a minimum of three recordings of 60 s were recorded and analysed using the NTA software 3.0 with default settings.

### 2.6 Transmission electron microscopy of purified microglial EVs

BV2‐derived EVs morphology was observed using transmission electron microscopy (TEM, Tecnai G2 Spirit Bio Twin Microscope (FEI, Eindhoven, The Netherlands)). Twenty‐microliter droplets of microglial EVs resuspended in PBS were placed on a clean Parafilm, after which formvar‐nickel TEM slots were placed on top of the droplets and allowed to stand for 60 min to adsorb the fluid. Slots with adherent EVs were washed and fixed with 2% glutaraldehyde for 10 min. After washing steps, slots were transferred to droplets of 2% uranyl acetate for 15 min. Finally, the slots were embedded in 0.13% methyl cellulose and 0.4% uranyl acetate and dried before examination with a transmission electron microscope at 120 kV.

### 2.7 Western blotting

The BV2 derived EVs or BV2 and C20 cells were lysed in ice‐cold RIPA buffer (ThermoFisher, 89900), supplemented with protease (Roche, 05892970001) and phosphatase inhibitor (Roche, 04906845001) for 30 min on ice and cleared at 17,000 × *g* force for 15 min. Protein concentrations were determined using Pierce BCA protein assay (ThermoFisher Scientific, 23227) according to the manufacturer's protocol. Afterward, proteins were denatured at 95°C for 5 min with 1× sample buffer, equal loaded and separated on a 12 or 15% SDS‐PAGE gel, transferred to a polyvinylidene difluoride membrane (Immobilon, IPVH00010) and subsequently blocked for 1 h either using 5% milk powder or 5% BSA (Life Sciences, A1324) in PBS (Lonza, 17–516F) solution containing 0.1% Tween 20 (VWR, MERC8.22185.0500). After blocking, membranes were incubated overnight at 4°C in the presence of a specific primary antibody. Horseradish peroxidase‐conjugated secondary antibodies (Dako, Denmark) at a dilution of 1/2000 were used for 1 h at room temperature. Visualization was performed using Pierce ECL Plus Western Blotting Substrate (ThermoFisher Scientific, 32132) for cell lysate or the WesternBright Sirius detection kit (Advansta, K‐12043‐D10) for EVs lysate. Band intensities were determined by quantifying the mean pixel grey values using the ImageJ software. The following primary antibodies were used in this study: anti‐CD81 (Cell Signalling Technology, D502Q), anti‐Annexin A2 (Cell Signalling Technology, D1162), anti‐Flotillin‐1 (Cell Signalling Technology, D2V7J), anti‐Grp94 (Cell Signalling Technology, D6 × 2Q), anti‐LC3B (Sigma‐Aldrich, L7543), anti‐Caspase‐1 (Cell Signalling Technology, D7F10), anti‐Caspase‐3 (Cell Signalling Technology, 8G10), anti‐Caspase‐8 (Cell Signalling Technology D35G2), anti‐β‐actin (Santa Cruz Biotechnology, sc‐47778). The following secondary antibodies were used: polyclonal Rabbit Anti‐Mouse Immunoglobulin/HRP (Dako, P0260), and polyclonal Goat Anti‐Rabbit Immunoglobulin/HRP (Dako, P0448).

### 2.8 Microglia EVs uptake by flow cytometry and immunofluorescence microscopy

BV2 or C20 cells were seeded in complete medium with or without a coverslip in 12 or 24‐well plates, respectively, at a density of 5 × 10^4^ or 1 × 10^5^ cells per well. After 48 h, cells were washed with PBS (Lonza, 17–516F) and stimulated with microglia EVs in reduced medium. EVs were harvested from non‐activated microglia cultures (nEVs) or TNFα‐activated microglia cultures (aEVs). Equal volumes were labelled with 555 nm Vybrant DiI Cell‐Labeling Solution (Life Technologies, 15704352) for 30 min at 37°C, and purified by size exclusion chromatography to remove unincorporated dye. NTA was performed to determine EVs concentration and EVs uptake assay optimizations were performed using 1×, 2× or 5 × 10^9^ of the DiI‐labelled EVs per ml. Optimization showed that a concentration of either 1× or 2 × 10^9^ EVs per ml volume is sufficient for downstream experiments.

Cultures plates containing 1 ml (12 well) or 0.5 ml (24 well) reduced medium, were incubated for 3 h at 37°C to allow EVs uptake. Cells seeded without coverslip were harvested using trypsin EDTA (Sigma‐Aldrich, T3924) and centrifuged for 5 min at 300 × *g* force. Supernatant was discarded and cell pellets were resuspended in 500 μL PBS containing 4% FBS (Biowest, S181B). Single cell mean fluorescent intensities were quantified at a wavelength of 555 nm by flow cytometry on a BD‐LSRFortessa flow cytometer (BD Biosciences) using FACSDiva software (BD Biosciences). The C20 cells seeded on a coverslip were fixed with 4% paraformaldehyde (ChemCruz, sc‐281692) at room temperature for 30 min. Nuclear staining was performed by 4′,6‐diamidino‐2‐phenylindolene (DAPI, Thermo Fisher Scientific, 62248) for 30 min at room temperature and mounted on a cover glass with Shandon Immu‐Mount medium (Thermo Fisher Scientific, 9990402). Cells were visualized with the ZEISS LSM880 Airyscan laser scanning confocal microscope using a 40×/1.3 Plan‐Neofluar objective. All images used for quantification were acquired using identical fluorescence excitation and detection settings that avoid channel crosstalk and three replicate wells were used per condition. Image analysis and quantification were performed using CellProfiler software (Kamentsky et al., 2011). An image analysis pipeline was designed in CellProfiler to automatically quantify the number of EVs punctate structures (DiI‐labelled EV puncta) per individual cell. To this end, nuclei were segmented using the DAPI channel (Adaptive thresholding Otsu method) and used as seeds to segment the cells (Propagation method with global Otsu thresholding). Punctate structures were segmented in the appropriate channel using the global MoG thresholding channel. Standard CellProfiler modules were used for relating the different masks, for measurements of shape and intensity parameters and to create output tables. Statistical analysis was then performed in GraphPad Prism.

### 2.9 Immunocytochemistry and confocal microscopy

Immunostainings were performed according to standard protocols. Briefly, 5 × 10^4^ C20 cells or 1.5 × 10^5^ primary microglia cells per well were seeded on a glass coverslip. After 24 h, complete medium was changed by reduced medium and cells were TNFα (20 ng/ml) activated and stimulated with 1 × 10^9^ nEVs or aEVs per ml for 48 h in reduced medium. Autophagy induction was performed using 10 μM Rapamycin (Enzo, ENZ‐51031) for 24 h or by serum deprivation for 3 h. Inhibition of lysosomal degradation was performed using 10 μM of chloroquine for 18 h (Enzo, 51005‐CLQ). Fixation was performed by incubating cells in ice‐cold methanol (VWR, 30847) for 20 min. Afterward, cells were permeabilized using 0.1% Triton X‐100 (VWR, 437002A) in PBS (Lonza, 17–516F). Blocking was performed with 5% BSA (Life Sciences, A1324) diluted in PBS for 1 h. The primary LC3B antibody was incubated overnight at 4°C and the secondary antibody was incubated for 1 h, diluted in 5% BSA in PBS supplemented with 0.1% Tween 20 (VWR, MERC8.22185.0500). Nuclear staining was performed by using DAPI (Thermo Fisher Scientific, 62248). After staining, cells were mounted with fluorescent mounting medium (Thermo Scientific, 9990402). Confocal microscopy images were taken on a Zeiss LSM880 Airyscan laser scanning confocal microscope as described above. All images used for quantification were acquired using identical fluorescence excitation and detection settings that avoided channel crosstalk. Two replicate wells were used per condition. An image analysis pipeline was designed in CellProfiler to automatically quantify the number of punctate structures (LC3B, mean fluorescence intensity) per individual cell as described above.

### 2.10 Autophagy induction and treatment

Induction of autophagy was performed by serum starvation by culturing cells in FBS deprived culture medium for 3 h at 37°C or by stimulating cells with 10 μM rapamycin for 24 h. To block degradation of autophagosomes by lysosomes, the lysosomal inhibitor bafilomycin A_1_ (Invivogen, tlrl‐baf1) or chloroquine (Enzo, 51005‐CLQ) was used at a concentration of respectively 10 nM and 10 μM.

### 2.11 CYTO ID autophagy assay

Autophagy was measured using the CYTO‐ID Autophagy Detection Kit (Enzo, ENZ‐51031) according to the manufacturer's protocol. Briefly, 5 × 10^4^ BV2 cells or 1 × 10^5^ primary microglia cells were seeded in respectively 12‐well or 96‐well plates in complete medium. The next day, complete medium was changed by reduced medium and cells were activated using TNFα and stimulated with 1 × 10^9^ nEVs or aEVs per ml for 48 h in reduced medium. Autophagy induction was performed using 10 μM Rapamycin (Enzo, ENZ‐51031) for 24 h. Lysosomal degradation was blocked by using 10 μM chloroquine for 18 h (Enzo, 51005‐CLQ). Cells were harvested, centrifuged, PBS‐washed, again centrifuged, and resuspended in 0.5 ml assay buffer. Subsequently, cells were stained with the CYTO‐ID green detection dye to measure autophagic vacuoles for 30 min at 37°C in the dark. After centrifugation, supernatant was discarded and the pellet was resuspended in 0.5 ml assay buffer. Single cell mean fluorescent intensities were quantified at a wavelength of 488 nm by flow cytometry on a BD‐LSRFortessa flow cytometer (BD Biosciences) using FACSDiva software (BD Biosciences).

### 2.12 Transmission electron microscopy assessment of autophagy

Human microglia C20 cells (5 × 10^4^), were grown in 8‐well chambered Permanox slides (Nunc, Lab‐Tek, C7182‐1PAK) and stimulated with 1 × 10^9^ nEVs or aEVs in 400 μL reduced medium. As a positive control, to induce accumulation of autophagosomes, the lysosomal inhibitor bafilomycin A_1_ (Invivogen, tlrl‐baf1) at a concentration of 10 nM was used. Cells were fixed in 0.1 M sodium cacodylate‐buffered, pH 7.4, 2.5% glutaraldehyde solution for 2 h at 4°C. After rinsing in 0.1 M sodium cacodylate, pH 7.4 (Sigma‐ Aldrich, 6131‐99‐3) containing 7.5% saccharose (Sigma‐Aldrich, 57‐50‐1), cells were post‐fixed in 1% OsO4 solution (Sigma‐Aldrich, 20816‐12‐0) for 1 h. After dehydration in an ethanol gradient, cells were embedded in EM‐bed812 (Electron Microscopy Sciences, EMS14120). Ultrathin sections were stained with lead citrate and samples were examined in a Tecnai G2 Spirit Bio Twin Microscope (FEI, Eindhoven, The Netherlands) at 120 kV. Quantification of autophagic vesicles was achieved by manually counting vesicles in 20 images per condition. Images were blinded and randomly taken at a magnification of 26,000x in the cytoplasm of the cells. Autophagic vesicles were identified based on ultrastructural characteristics as described by Ylä‐Anttila et al (Yla‐Anttila, Vihinen, Jokitalo, & Eskelinen, 2009).

### 2.13 RNA sequencing

Human microglia C20 cells were seeded at a density of 1 × 10^6^ cells per flask either activated with 20 ng/ml TNFα or non‐activated. Cells were stimulated with M‐EVs (nEVs or aEVs) at a concentration of 1 × 10^9^ EVs per million cells for 48 h. Non‐activated or TNFα only‐activated cells were used as controls. Targeted RNA sequencing libraries were prepared and sequenced using the MiSeq technology. In short, 400 ng of total RNA is converted into cDNA using the QIAseq targeted RNA first strand synthesis component reagents (The human Inflammation & Immunity Transcriptome QIAseq RNA panel RHS 005Z). The panel contains cytokines, chemokines, pro‐ or anti‐apoptotic genes, growth factors and their receptors, downstream signalling pathway genes and transcription factors involved in coordinating diverse functions of the immune system. Additionally, rRNA depletion, blocking or poly‐A selection of mRNA is not necessary. The RNA panels integrate a molecular barcode or tag (MT) technology into a highly multiplexed PCR‐based target enrichment process, enabling unbiased and accurate quantification of a targeted panel of mRNA transcripts by next generation sequencing (NGS). The MT assignment step makes use of a multiplex primer panel (targeting 475 genes and 25 reference normalization genes) and an input of 40 ng cDNA. After the molecular barcoding step with a gene specific primer 1 (GSP1), the barcoded cDNA is purified over QIAseq beads to remove residual primers/dimers, and a PCR reaction is set up with a second pool of gene‐specific primers (GSP2) and the common uni1 primer (RS2), which anneals to a common sequence present on the GSP1 primers. This reaction ensures that intended targets are enriched sufficiently to be represented in the final library. A universal PCR is then run to amplify the products and add sample indexing as a final step. The purified libraries were then normalized and the multiplexed samples were pooled into a single library for sequencing. Single‐end 150 bp sequencing was performed on a Miseq platform (Illumina) using the QIAseq Read 1 Primer 1 custom primer (Qiagen, Germany). A total of 4.7 Gb was obtained from a 1149 K/mm^2^ cluster density with cluster passing quality control filters at 94.95% (28,573,092 clusters).

### 2.14 RNA sequencing data processing

The quality of raw sequence reads was checked using FastQC version 0.11.8, and nucleotide calls with a quality score of 28 or higher were considered of very good quality. Adapters were removed using cutadapt v.2.4 (Martin, 2011). The reads were then aligned to the hg19 genome reference using STAR (2.5.0e). A maximum of two mismatches was allowed. All other options were set as STAR default values. Gene counts were retrieved using htseq‐count (Anders, Pyl, & Huber, 2015) using the “union” option. After removing absent features (zero counts in all samples), the raw counts were imported to R/Bioconductor package DESeq2 v.3.9 (Love, Huber, & Anders, 2014) to identify differentially expressed genes among samples. The default DESeq2 options were used, including log fold change shrinkage. Differentially expressed genes were considered only when the Benjamini‐Hochberg adjusted *P* value (false discovery rate [FDR]) was <0.05. Heatmap hierarchical clustering and bar plots were created using the gplots::heatmap.2() and barplot() function, respectively, on transformed raw counts. Differentially expressed gene data were generated for each condition. The abundance of RNA transcripts was evaluated using differentially expressed gene analysis and clustergram was used to display a heatmap indicating changes in genes in individual samples.

### 2.15 Pathway analysis

Ingenuity Pathway Analysis (IPA; Ingenuity Systems/Qiagen, Redwood City, CA, USA) was used to map lists of significant genes (FDR < 0.05) to gene ontology groups and biological pathways. The functional and canonical pathway analysis was used to identify the significant biological functions and pathways. iPathwayGuide analysis was used for further impact and network analysis (Advaita Bioinformatics, Ann Arbor, MI, USA). Functions and pathways with a *P* ‐value less than 0.05 (Fisher's exact test) were considered to be statistically significant.

### 2.16 Quantitative PCR

For quantitative polymerase chain reaction (qPCR) on cells stimulated with microglia EVs, 2 × 10^5^ C20 cells were seeded in six‐well plates in complete medium. After 24 h, complete medium was changed by reduced medium and cells were stimulated with 2 × 10^9^ nEVs or aEVs in 2 ml reduced medium for 24 h, followed by 20 ng/ml TNFα‐activation for 24 h. Subsequently, cells were harvested and total RNA was prepared using the RNeasy mini kit (Qiagen, 74106) according to the manufacturer's instructions. RNA quality was determined using a NanoDrop spectrophotometer (Isogen Life Science, IJsselstein, The Netherlands). Further, RNA was reverse transcribed using the qScript cDNA SuperMix (Quanta Biosciences, 95048) and quantitative PCR was performed on a StepOnePlus RT‐PCR System (Applied Biosystems, Halle Belgium). Fast SYBR Green (Applied Biosystems, 4385612), 10 μM forward and reverse primer mixture (Integrated DNA Technologies, Leuven, Belgium) and 10 ng RNA was used per reaction. Relative quantification of gene expression was accomplished using the ΔΔCt method. Expression levels were normalized using the most stable housekeeping genes determined with geNorm. Primer sequences can be found in Table S5.

### 2.17 ELISA

C20 cells were seeded and treated as previously described in the qPCR section. Cells were collected and lysed using cell extraction buffer PTR (Abcam, ab193970). Whole protein concentrations were determined using Pierce BCA protein assay (ThermoFisher Scientific, 23227) according to the manufacturer's protocol. Elisa kits for Human IL‐1β (Abcam, ab46052), caspase 1 (Abcam, ab219633), caspase 8 (Invitrogen, BMS2024) and FAS (Abcam, ab100515) were used to assess specific protein concentrations. Optical density of standards and samples was measured at OD 595 nm using a Multiskan FC Microplate Absorbance Reader (Thermo Scientific).

### 2.18 JC‐1 apoptosis assay

The JC‐1 Mitochondrial Membrane Potential Assay Kit (BD Biosciences, 551302) was used to investigate the level of cells in early apoptosis according to the manufacturer's protocol. In brief, 5 × 10^4^ BV2 cells were seeded in 12‐well plates in complete medium. After 48 h, complete medium was changed by reduced medium and cells were stimulated with 1 × 10^9^ nEVs or aEVs in 1 ml reduced medium for 24 h, followed by 20 ng/ml TNFα‐activation or 1 μM Valinomycin (Enzo, BML‐KC140) stimulation for 24 h. Subsequently, cells were harvested, centrifuged, PBS‐washed, again centrifuged, and resuspended in 0.5 ml assay buffer. Next, cells were stained with the JC‐1 green detection dye for 30 min at 37°C in the dark. After centrifugation, the supernatant was discarded and the pellet was resuspended in 0.5 ml assay buffer. Single cell mean fluorescent intensities were quantified at a wavelength of 488 and 594 nm by flow cytometry on a BD‐LSRFortessa flow cytometer (BD Biosciences) using FACSDiva software (BD Biosciences).

### 2.19 Annexin V phosphatidylserine (PS) apoptosis assay

In order to determine early apoptosis, phosphatidylserine (PS) exposure was measured using the PS‐binding protein Annexin V conjugated to a fluorescent FITC label. Therefore, 2.5 × 10^5^ primary microglia cells or 5 × 10^4^ BV2 and C20 cells were seeded in complete medium. The next day, complete medium was replaced by reduced medium and cells were stimulated with 1 × 10^9^ nEVs or aEVs per 1 ml reduced medium for 24 h, followed by TNFα‐activation for 24 h. Apoptosis was induced using 1 μM Valinomycin (Enzo, BML‐KC140) stimulation for 24 h. Cells were harvested, centrifuged and resuspended in 100 μl of assay buffer (Invitrogen, PNN1001). Per reaction, 5 μl Annexin V‐FITC (Invitrogen, 11‐8005‐74) and 2.5 μl 7AAD dye (BD Pharmingen, 51–68981E) were incubated in the dark for 15 min at room temperature. Afterward, cells were centrifuged, washed and resuspended in 1× assay buffer. Single cell fluorescent intensities were quantified by flow cytometry on a BD‐LSRFortessa flow cytometer (BD Biosciences) using FACSDiva software (BD Biosciences).

### 2.20 Statistical analysis

Statistical analyses were performed using Student's two‐tailed t‐test or ANOVA unless noted otherwise. Data were analyzed using the GraphPad Prism software and presented as the mean ± SEM. Values of *P* < 0.05 were considered statistically significant.

### 2.21 EV track

We have submitted all relevant data of our experiments to the EV‐TRACK knowledgebase (EV‐TRACK ID: EV190095, EV‐TRACK score: 78%).

## 3 RESULTS

### 3.1 Characterization of microglia‐derived small extracellular vesicles

**FIGURE 1:**
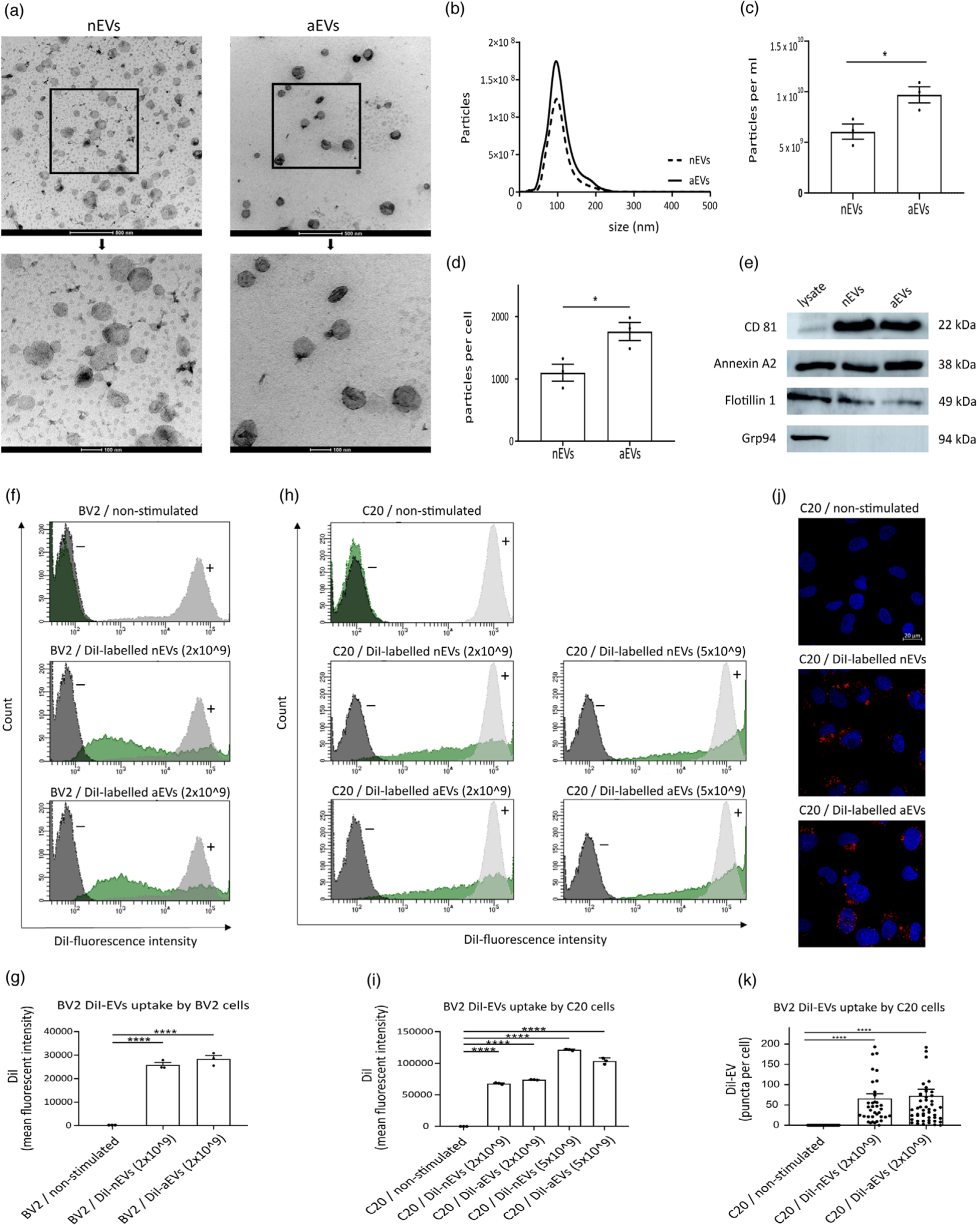
Characterization and uptake of microglia‐derived small extracellular vesicles. (a) Representative overview (scale bar 500 nm) and cropped (scale bar 100 nm) image showing microglial small extracellular vesicle morphology revealed by transmission electron microscopy (TEM) of non‐activated microglia‐derived EVs (nEVs) and TNFα‐activated microglia derived EVs (aEVs). (b‐d) Particle size profile distribution, particles per ml and particles per cell measured by Nanoparticle Tracking Analysis (NTA) of nEVs and aEVs, pelleted from 20 ml microglia cell culture medium and re‐suspended in 1 ml of PBS. (e) Western blotting analysis of small extracellular vesicle protein markers CD81, Annexin A2, Flotillin 1 and as negative control marker Grp94. Quantification of cellular uptake of DiI‐labelled nEVs and aEVs was analyzed by (f‐i) flow cytometry and by (j‐k) confocal microscopy. Merged representative images of DiI (red) and the DAPI nucleus stain (blue) are shown (scale bar: 20 μm). Fluorescence intensities of DiI‐labelled nEVs or aEVs per cell were analysed using CellProfiler software and GraphPad was used for statistical analysis. One‐way ANOVA Dunnett's multiple comparison test was used to determine significance (*P < 0.05, ****P < 0.0001). Data are presented as means ± SEM (n is three biological replicates per group)

The isolated microglia‐derived small extracellular vesicles (nEVs and aEVs) were characterized according to the MISEV 2018 guidelines (Théry et al., 2018). Transmission electron microscopy (TEM) images revealed that both nEVs and aEVs had a similar cup‐shaped or spherical morphology (Figure 1a). Nanoparticle tracking analysis (NTA) showed that the distribution curves of the particle size of nEVs and aEVs were similar (Figure 1b). NTA results demonstrated a significant increase in particles per ml and particles per cell of aEVs compared to nEVs (Figure 1c,d). We confirmed that 24 h of TNFα− activation of microglia BV2 cells did not affect their proliferation rate (Figure S2A‐C). Furthermore, western blotting revealed the presence of the EV markers CD81, Annexin A2 and Flotillin1 in both nEVs and aEVs. To confirm the purity of nEVs and aEVs, we analyzed for the absence of the endoplasmic marker Grp94. Our data indicate the absence of Grp94 in both nEVs and aEVs protein lysate (Figure 1e). Together, these analyses confirmed the successful isolation of EVs from BV2 microglia conditioned cell culture media.

### 3.1 Internalization of microglial‐EVs by microglia cell lines

In order to characterize the biological activities of microglia‐EVs (M‐EVs) in cells, the internalization of M‐EVs by donor (BV2) and recipient (C20) cells was investigated. EVs derived from a donor cell can incorporate into a recipient cell by a paracrine mechanism of action (Hood, Pan, Lanza, & Wickline, 2009). To examine the uptake of nEVs and aEVs in microglial cell lines, we investigated whether fluorescent DiI‐labelled EVs could be incorporated into these different cell types. Cellular uptake of fluorescently labelled EVs was evaluated by flow cytometry and confocal microscopy techniques.

Using flow cytometry, we observed that both subsets of DiI‐labelled M‐EVs (nEVs and aEVs), were incorporated into both microglia subtypes (Figure 1f‐i). After 3 h of EVs uptake in cells, DiI fluorescence was detected in cells stimulated with the labelled nEVs or aEVs, but not in the non‐stimulated control cells without EVs, supporting the occurrence of interaction between labelled EVs and the recipient cells (Figure 1f‐i). There were significant differences observed in the uptake of labelled EVs when compared to control cells without labelled EVs in both the BV2 microglia (Figure 1f‐g) and C20 microglia (Figure 1h‐i). Of note, the internalization of EVs in cells is dependent on the concentration of EVs used as a significantly higher uptake of EVs was observed when an EVs concentration of 5 × 10^9^ per ml was used compared to an EVs concentration of 2 × 10^9^ per ml (Figure 1i). Overall, no difference in cell uptake was observed when nEVs uptake was compared to aEVs uptake in both the BV2 and C20 microglia cells (Figure 1g,i). Furthermore, we examined the localization of the internalized M‐EVs in the human microglia C20 recipient cells. A similar result was observed using confocal microscopy, consistently showing that both nEVs and aEVs were incorporated into recipient cells (Figure 1j‐k). Taken together, these results show that M‐EVs can be taken up into different microglial cell types and that the level of internalized EVs is similar between nEVs and aEVs but can be influenced by the particle number.

### 3.2 M‐EVs activates autophagy in microglia cell lines and in primary cells

To investigate whether the internalized EVs could modify autophagy activities in non‐activated and TNFα‐activated microglia, we evaluated the effect of nEVs and aEVs incorporated in BV2 and C20 microglia cell lines as well as in primary microglia cells. Autophagy plays an important role in the protection of cells under stress and can actively influence the activities of inflammatory responses in microglia (Jin et al., 2018). C20 microglia and primary cells were cultured with or without M‐EVs and immunofluorescence analysis was performed using microtubule associated protein 1 light chain (MAP1LC3/LC3) antibody. The LC3 autophagy marker is usually converted from the LC3‐I form to the lipidated LC3‐II form, which correlates with the number of autophagosomes (Mizushima & Yoshimori, 2007). As a control group, cells were left non‐stimulated (PBS, negative control) or treated for 3 h with serum deprivation or rapamycin, an inducer of autophagy (positive control), and the lysosomal blocker bafilomycin A1 (Baf) or chloroquine.

**FIGURE 2:**
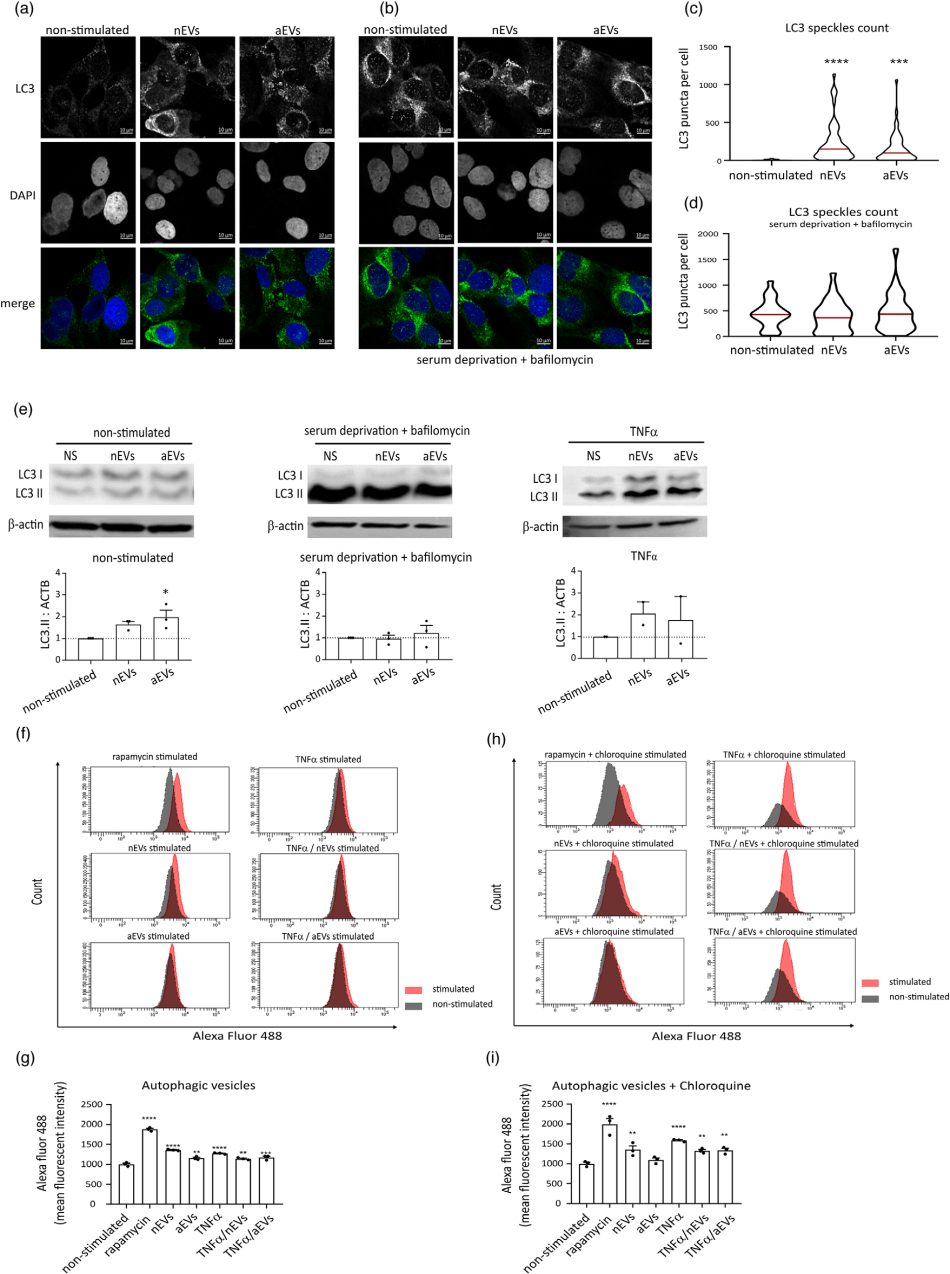
Microglia EVs pre‐treatment activates autophagy in microglia cell lines. (a‐b) Immunofluorescence staining of human C20 microglia cells stimulated with nEVs and aEVs. Cells were either treated or not with serum starvation and bafilomycin A1 (Baf) for 3 h. Non‐stimulated cells were used as controls. Representative image showing, LC3 (green), the DAPI nucleus stain (blue) and merged images (scale bar 10 μm). (c‐d) Cell Profiler quantification of LC3‐positive puncta detected by immunofluorescence (n = 3 biological replicates per group, 30+ cells per experiment). Data are expressed as means, ***P < 0.001, ****P < 0.0001. (e) Western blotting analysis of LC3‐II in C20 cells exposed to nEVs or aEVs either under non‐activated conditions or activated with TNFα or 3 h starvation and bafilomycin A1. The levels of LC3‐II were calculated from three independent western blotting experiments (n = 3 biological replicates), normalized to β‐actin. Band intensities were determined by quantifying the mean pixel gray values using ImageJ software. Data are presented as mean ± SEM (*P < 0.05). (f‐i) Flow cytometry monitoring of CYTO‐ID autophagic flux in BV2 cells using the CYTO‐ID green detection dye that selectively labels accumulated autophagic vacuoles. Data are normalized to non‐stimulated control = 1000 MFI and shown as mean ± SEM (n = 3 biological replicates per group, 10,000 events per experiment). GraphPad was used to perform a one‐way ANOVA Dunnett's multiple comparison test to determine significance (*P < 0.05, **P < 0.01, ***P < 0.001, ****P < 0.0001)

**FIGURE 3:**
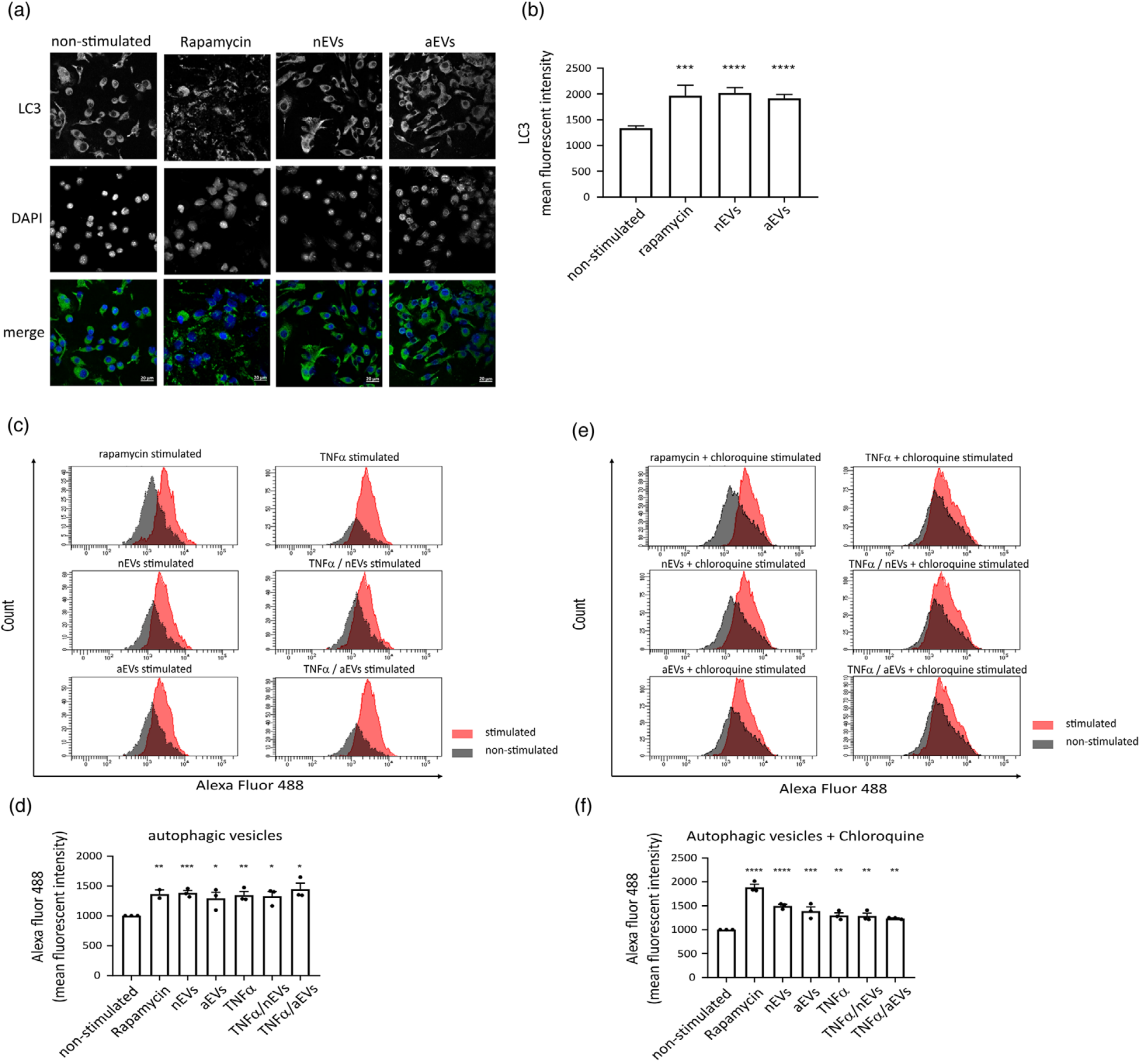
Microglia EVs pre‐treatment activates autophagy in primary microglia cells. (a) Immunofluorescence staining of mouse primary microglia cells stimulated with nEVs and aEVs. Non‐stimulated cells were used as negative control and rapamycin stimulated cells as positive control. Representative image showing, LC3 (green), the DAPI nucleus stain (blue) and merged images (scale bar 20 μm). (b) Cell Profiler quantification of LC3 fluorescence intensity detected by immunofluorescence (n = 5 biological replicates per group, 30+ cells per experiment). Data are expressed as means, ***P < 0.001, ****P < 0.0001. (c‐f) Flow cytometry monitoring of CYTO‐ID autophagic flux in primary cells using the CYTO‐ID green detection dye that selectively labels accumulated autophagic vacuoles. Data are normalized to non‐stimulated control = 1000 MFI and shown as mean ± SEM (n = 3 biological replicates per group, 10,000 events per experiment). GraphPad was used to perform a one‐way ANOVA Dunnett's multiple comparison test to determine significance (*P < 0.05, **P <0.01, ***P < 0.001, ****P < 0.0001).

Compared with the non‐stimulated (PBS) control group, both nEVs and aEVs exhibited significantly increased levels in LC3‐positive puncta in human cells (Figure 2a,c). A similar result was observed in mouse primary cells stimulated with M‐EVs (Figure 3a,b). Furthermore, in the presence of bafilomycin A1 treatment and serum deprivation, used to induce autophagy, no significant difference was observed between the Baf‐only‐treated group and the Baf + M‐EVs treated groups in C20 cells (Figure 2b,d). In line with this, C20 cells exposed to M‐EVs displayed a slightly higher amount of LC3‐II protein on western blots compared to non‐stimulated control cells (Figure 2e). Semi‐quantitation of the LC3‐II immunoblot showed a significant increase only for the aEVs stimulated cells but not for the nEVs stimulated cells. Serum deprivation and Baf treatment did not lead to a difference in LC3‐II expression between the Baf‐only‐treated group and the Baf + M‐EVs treated group (Figure 2e). A similar result was observed when TNFα−activated cells were exposed to M‐EVs, that is, a slight but non‐significant increase in LC3‐II level on western blot was observed, when the M‐EVs + TNFα−treated group was compared to TNFα‐only treated group (Figure 2e).

We further evaluated autophagy flux using the Cyto‐ID Autophagy Detection Kit with flow cytometry in BV2 microglia and in primary cells. As expected, stimulation with M‐EVs to either BV2 cell line or primary cells increased autophagy levels. A significant increase was observed for both the nEVs and aEVs stimulated cells compared with non‐stimulated control cells (Figure 2f,g). The same experiment was validated in primary microglia cells and results show that M‐EVs significantly activated autophagy in primary cells (Figure 3c‐d). In addition, cells stimulated with both M‐EVs and activated with TNFα also show a significant increase in autophagy flux both in BV2 (Figure 2f‐g) and in primary live cells (Figure 3c‐d). In addition, in the presence of chloroquine, which inhibits autophagic flux, M‐EVs showed a significant increase in autophagic flux in both BV2 (Figure 2h‐i) and primary microglia cells (Figure 3e‐f). Furthermore, in the presence of chloroquine, cells activated with TNFα to induce cellular stress, also showed a significant increase in autophagy flux (Figure 2i, 3f). Additionally, autophagy flux results were confirmed within 6 days after primary microglia isolation by CD11b‐MACS microbeads (Supplementary Figure S1C‐D), thereby indicating that primary microglia isolated with either CD11b‐MACS or the shake‐off method, both exhibited activation of autophagy upon stimulation with M‐EVs.

These results indicate that M‐EVs can activate autophagy through LC3B‐positive autophagosomes formation in microglia to maintain cellular homeostasis.

### 3.3 Formation of large autophagic vacuoles is activated in cells exposed to microglial EVs

**FIGURE 4:**
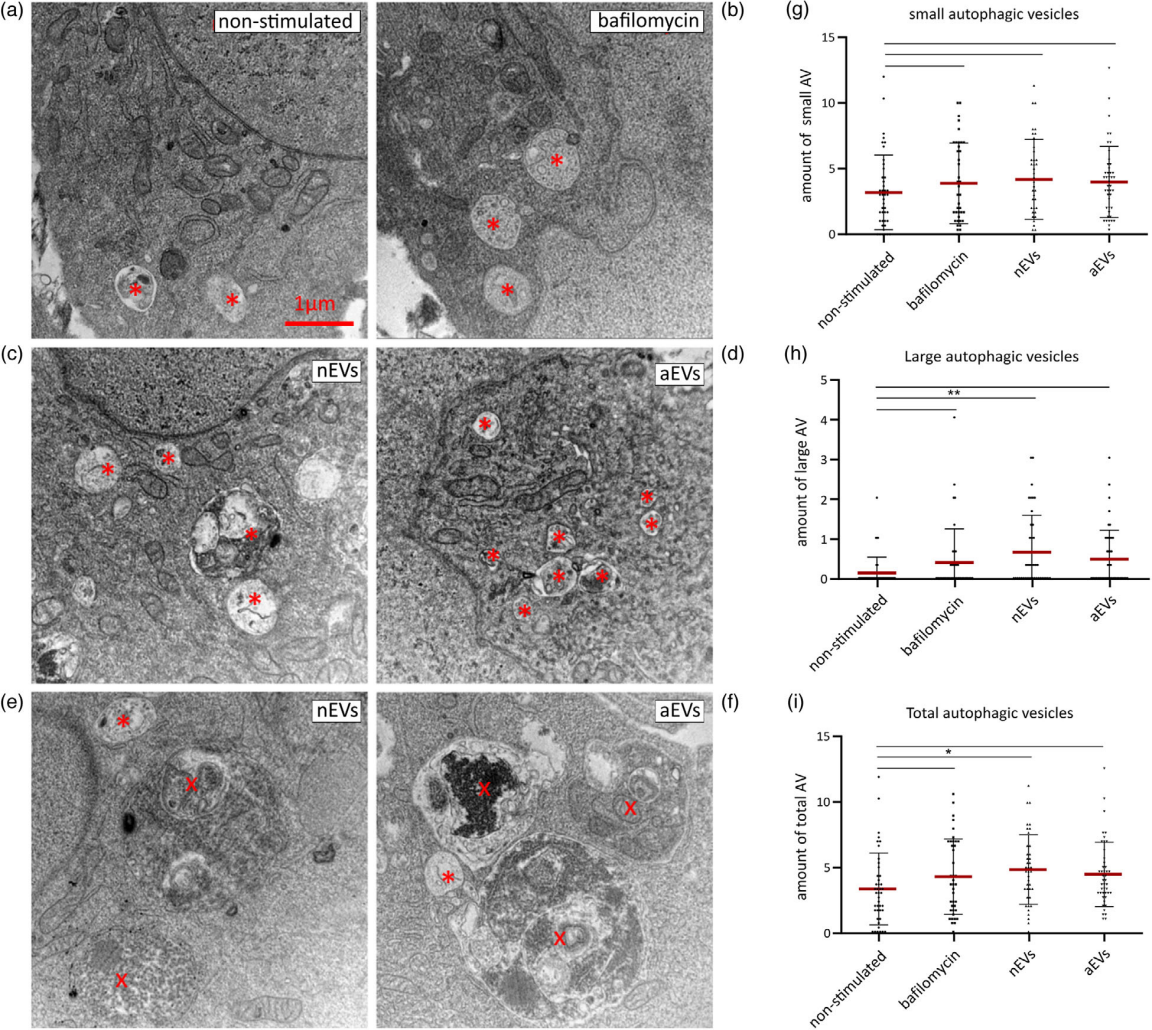
M‐EVs exposure increases the number of large autophagic vesicles in microglia. Transmission electron micrograph of microglia C20 cells in (a) non‐stimulated control, (b) treated with bafilomycin A1 or stimulated with (c,e) nEVs or (d,f) aEVs. Autophagic vesicles were identified based on ultrastructural morphological characteristics. Small autophagic vesicles (asterisk, A, B, C, D) could easily be recognized in all conditions. The number of small autophagic vesicles, including autophagosomes and autolysosomes, is slightly increased after EVs stimulation of microglia C20 cells. Exposing the microglia to nEVs or aEVs also induced the presence of very prominent large (auto) phagocytotic (cross) vesicles as seen in images E and F. Scale bar: 1 μm. (g‐i) Quantification of the number of small and large vesicles. Per cell, the total number of autophagic vesicles present on the section was counted and expressed per cytoplasmic area covered. Data are shown as mean ± SEM. GraphPad was used for statistical analysis. One‐way ANOVA Bonferroni multiple comparison test was used to determine significance

As autophagy plays an important role in protecting cells against stress, we examined, at the ultrastructural level, the effect of M‐EVs on autophagy activation in human C20 microglia. Bafilomycin A1 was used as a positive control to inhibit autophagy and to prevent maturation of autophagic vacuoles by inhibiting fusion between autophagosomes and lysosomes (Figure 4b). The autophagy levels in the microglia stimulated with nEVs and aEVs were evaluated and quantified using transmission electron microscopy (TEM). In this study, microglia contained abundant and very prominent vesicular structures. Two distinct groups of vesicular structures could be discriminated. On the one hand, we observed small autophagic vesicles (with diameters ranging from 0.2 to 1.0 μm) at various stages of degradation including autophagosomes and autolysosomes (Figure 4c‐d). On the other hand, large (auto)phagocytic vesicles (with diameters ranging from 1.5 to 3.8 μm), enclosing specific organelle fragments were found (Figure 4e‐f).

The results show that the total number of autophagic vesicles per 50 μm^2^ cytoplasm area were significantly higher in the nEVs stimulated cells compared to non‐stimulated control cells and cells treated with bafilomycin A1 (Figure 4i). Quantification did not reveal a significant difference in small autophagic vesicle numbers between nEVs or aEVs and bafilomycin A1‐treated groups (Figure 4g). The total number of large (auto)phagocytic vesicles per 50 μm^2^ cytoplasm area was significant higher in nEVs stimulated cells compared to non‐stimulated control cells (Figure 4h). All autophagy results combined suggest that M‐EVs induce autophagy in microglia in vitro.

### 3.4 Non‐activated microglia exposed to M‐EVs mediate multi‐target gene signaling

**FIGURE 5:**
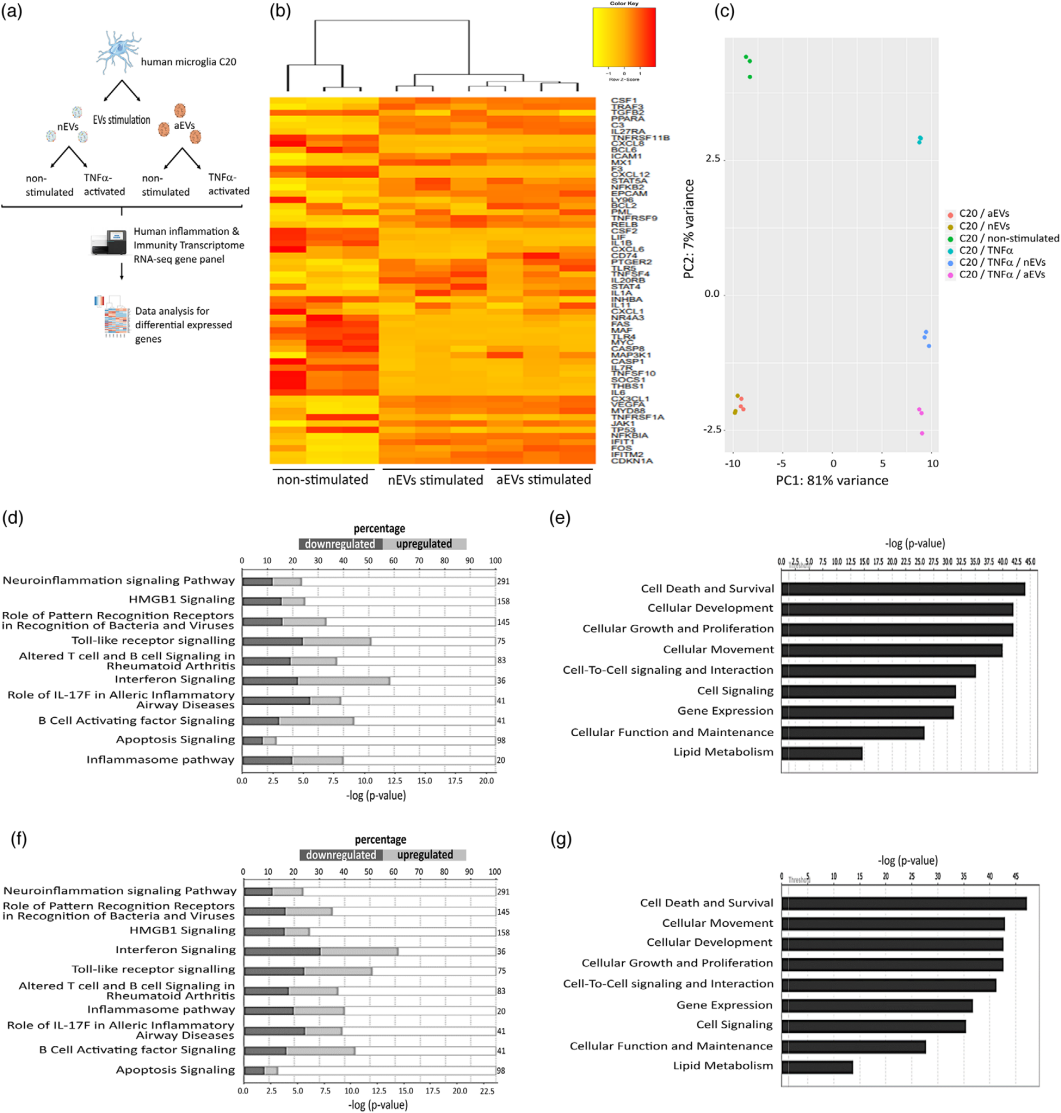
M‐EVs are multi‐target signaling mediators influencing microglial function and maintenance. (a) Design of the targeted RNA sequencing analysis. Human C20 microglia cells were stimulated with BV2‐derived nEVs or aEVs. After 24 h of incubation, cells were either activated with TNFα (20 ng/ml) or left non‐activated. After 48 h of incubation, targeted RNA‐sequencing libraries were prepared and sequenced using the Illumina Miseq technology. After sequencing, data analysis for differentially expressed genes was performed. (b) Hierarchical clusterization of 50 differentially expressed genes in C20 microglia stimulated with nEVs or aEVs compared to non‐stimulated control cells. Yellow marks low expression and orange‐red marks high expression (n = 3 per group). A complete list of differentially expressed genes can be found in Tables ST1‐ST2. (c) PCA plot showing disparities of microglia stimulated with M‐EVs, and corresponding non‐stimulated or TNFα‐activated controls. (d,e) List of top significant IPA canonical pathways and molecular function and their P‐value in non‐stimulated C20 microglia versus nEVs‐stimulated C20 microglia and (f,g) aEVs‐stimulated cells compared to non‐stimulated control cells. All RNA sequencing data represent the average of three biological replicates

The biological activities occurring at the transcriptional level in the C20 human microglia after internalization of M‐EVs were elucidated. Accordingly, the gene expression profiles, potential molecular and canonical pathways were evaluated. The design of the targeted RNA sequencing using the human inflammation and immunity transcriptome gene panel performed in this study is shown in Figure 5a. We profiled the expression of 475 genes involved in regulating immune and inflammatory processes and assessed the effects of nEVs and aEVs on the cell survival pathway and related biological processes.

We investigated the relatedness of transcriptional level gene expression of microglia exposed to M‐EVs, and corresponding non‐stimulated or TNFα‐activated controls using principal component analysis (PCA) (Figure 5c). Analysis of the relationship using the first and second principal components showed separately distinct clusters. When comparing the microglia gene signature after exposure to the different M‐EV subsets, we found that in microglia stimulated with nEVs, 311 out of 475 genes that were differentially expressed (24% downregulated: 74 of 311 genes and 24% upregulated: 70 of 311 genes; *P*  < 0.05 and log2fold change ≥ ± 0.5). Analysis of microglia stimulated with aEVs presented 319 out of 475 differentially expressed genes (24% downregulated: 79 of 319 genes and 21% upregulated: 68 of 319 genes; *P*  < 0.05 and log2fold change ≥ +‐ 0.5). We further analyzed expression patterns of differentially expressed genes by unsupervised hierarchical clusterization between non‐stimulated control cells and cells stimulated with either nEVs or aEVs. We found similar changes in the gene expression (log2 fold difference ≥ ± 0.5) between normal C20 microglia exposed to nEVs or aEVs subsets, which were normalized against the mean expression level of the non‐stimulated controls cells. Further, we observed that the gene expression profile of key genes, that are involved in cell death and survival including caspase 1 and caspase 8, were downregulated compared to non‐stimulated control cells (Figure 5b). Therefore, we investigated the impact of M‐EVs on the cell death and survival pathway and related biological processes. Using ingenuity pathway analysis (IPA) we were able to predict deregulation of genes in several canonical signaling pathways such as the neuroinflammation, inflammasome and apoptosis signalling pathways (Figure 5d,f). Prediction of molecular and cellular function showed that genes involved in the cell death and survival pathway were most significantly affected (Figure 5e,g). Notably, we did not observe obvious changes in the canonical or functional prediction pathways when the nEVs group was compared to the aEVs group, suggesting that the gene expression pattern of both nEVs and aEVs subsets are relatively similar (Figure 5b‐g). In addition, the involved pathological conditions include disorders affecting inflammatory responses and immunological or neurological diseases (data not shown). Collectively, these results suggest that M‐EVs subsets can influence multiple genes and biological processes in microglia to modulate neuroinflammation, cell death and survival signaling pathways.

### 3.5 M‐EVs are multimodal signalling mediators in TNFα‐activated microglia

In line with the previous section, we investigated if M‐EVs could reduce apoptosis and deregulate the abundance of RNA transcripts involved in neuroinflammation, cell death and survival signalling pathways in TNFα‐activated microglia. The same targeted RNA sequencing protocol as previously described (Figure 5a) was used to perform transcriptome profiling in TNFα‐activated microglia stimulated with M‐EVs subsets.

**FIGURE 6:**
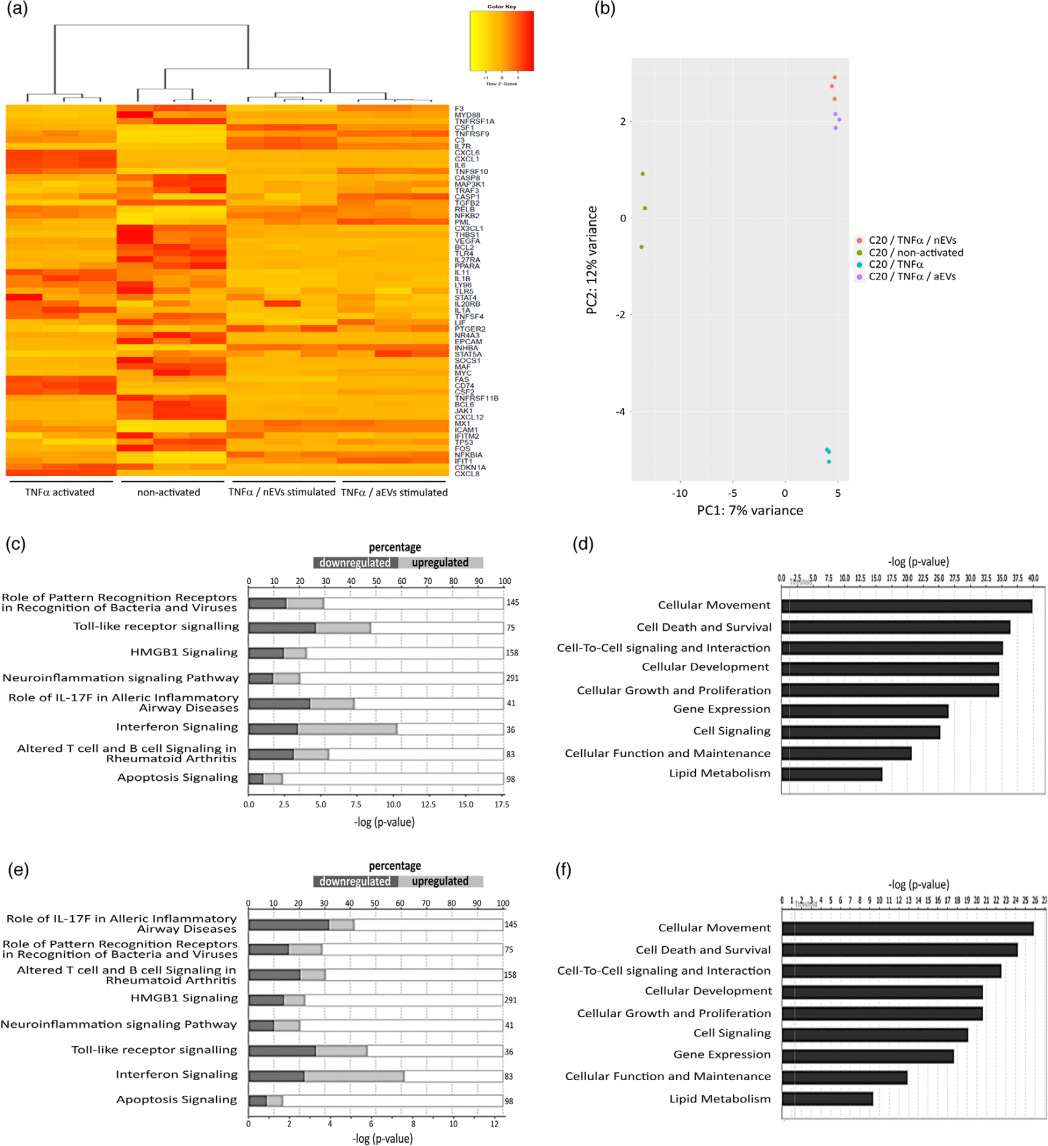
M‐EVs are multimodal signaling mediators in TNFα‐activated cellular inflammation. (a) Hierarchical clusterization of 50 differentially expressed genes in C20 microglia stimulated with a combination of TNFα and nEVs or aEVs compared to only TNFα−activated control cells and non‐activated control cells. Yellow marks low expression and orange‐red marks high expression (n = 3 per group). A complete list of differentially expressed genes can be found in Tables ST3‐ST4. (b) PCA plot showing disparities of microglia activated with TNFα, either non‐stimulated or M‐EVs stimulated. (c,d) List of top significant IPA canonical pathways and molecular function and their P ‐values in TNFα‐activated microglia versus TNFα/nEVs stimulated microglia and (E,F) TNFα/aEVs stimulated cells compared to TNFα‐activated control cells. All RNA sequencing data represent the average of three biological experiments

As controls, microglia cells activated with TNFα only, and non‐activated control cells were used to compare gene expression changes to cells activated with TNFα and either stimulated with nEVs or aEVs (Figure 6a). PCA data shows that microglia, exposed to M‐EVs + TNFα were well separated into distinct clusters using all genes detected (Figure 6b). In TNFα + nEVs stimulated cells, we detected 286 out of 475 genes were differentially expressed (20% downregulated: 56 of 286 genes and 11% upregulated: 31 of 286 genes; *P*  < 0.05 and log2fold change ≥ ± 0.5). In microglia exposed to TNFα + aEVs, 288 of 475 genes were differentially expressed (18% downregulated: 51 of 286 genes and 13% upregulated: 36 of 286 genes *P*  < 0.05 and log2fold change ≥ ± 0.5). Additionally, the unsupervised hierarchical clusterization (Figure 6a) shows very similar changes in the expression of 50 differentially expressed genes (log2 fold difference ≥ ± 0.5) when comparing TNFα‐activated microglia stimulated with nEVs or aEVs subsets. Data were normalized against the mean of the TNFα only‐activated control group. In line with previous data, we observed that upon TNFα‐activation and M‐EVs stimulation, the gene expression profiles of key genes involved in cell death and survival pathways including IL1β, FAS, TRAIL (TNFSF10) were either reduced or not affected compared to TNFα  only activated microglia (Figure 6a). We investigated the impact of TNFα + M‐EVs on the cell death and survival pathway and related biological processes. Using ingenuity pathway analysis (IPA) we could predict deregulation of genes in several canonical signalling pathways such as the neuroinflammation, interferon and Toll‐like receptor signalling pathways (Figure 6c,e). Molecular and cellular function prediction analysis showed that genes involved in cellular movement and cell death and survival pathway were most significantly affected (Figure 6d,f). In line with previous data, we did not observe obvious changes in the canonical or molecular and cellular prediction analysis when the TNFα + nEVs group was compared to the TNFα + aEVs group. The results suggest that the gene expression pattern of both TNFα + M‐EVs subsets could communicate in a relatively similar manner (Figure 6a‐e). The pathological conditions, mostly predicted to be associated include diseases affecting immunological and inflammatory responses (data not shown). Together, these results suggest that TNFα + M‐EVs can mediate multimodal signalling to reduce apoptotic crosstalk and modulate the abundance of RNA transcripts involved in neuroinflammation, interferon and toll‐like receptor signalling pathways in TNFα‐activated microglia.

### 3.6 Meta‐analysis of differentially expressed genes in TNFα‐activated and non‐activated microglia exposed to M‐EVs

**FIGURE 7:**
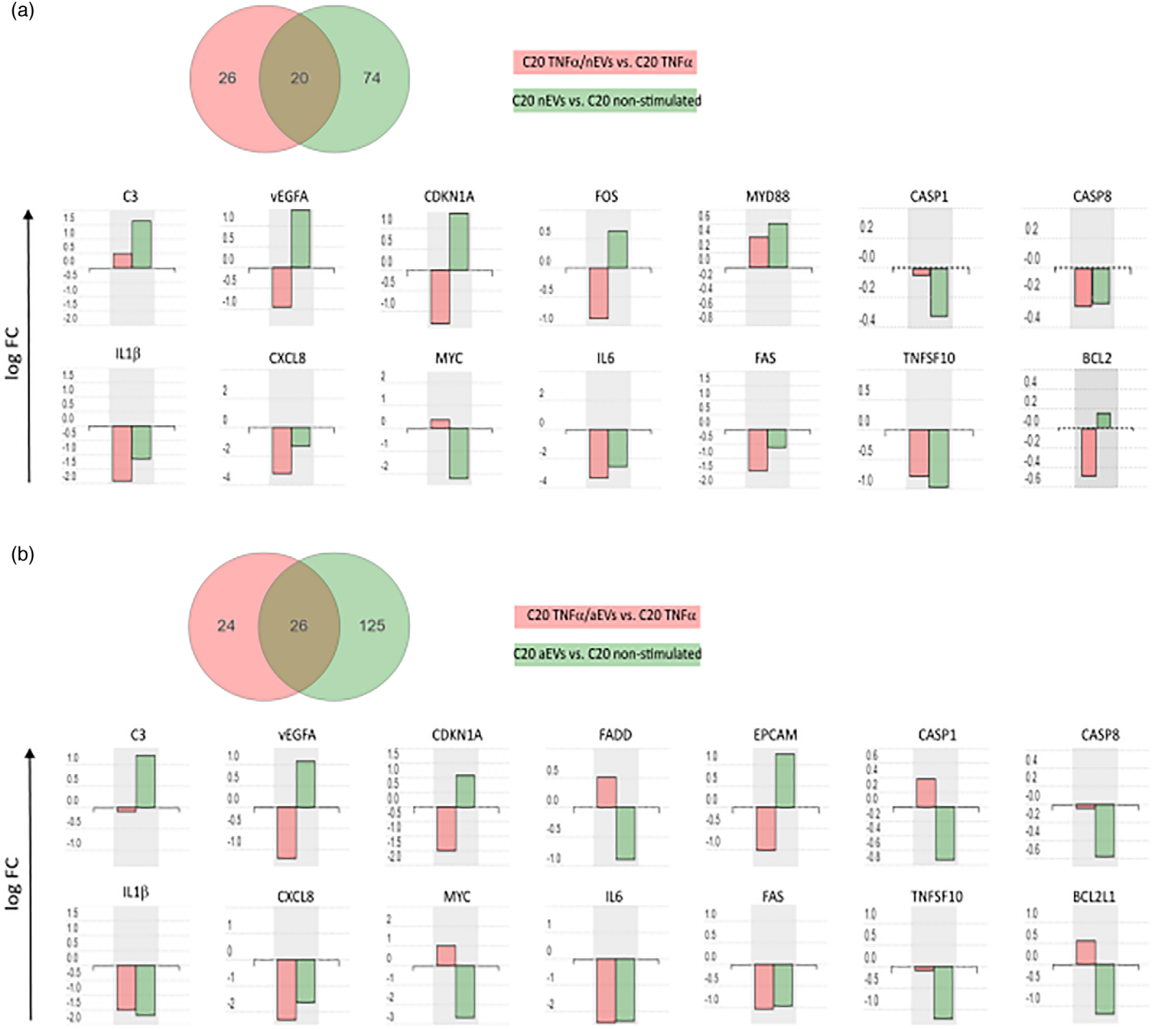
Meta‐analysis of differentially expressed annotated genes involved in cell death and survival signaling in the presence and absence of TNFα‐induced cellular inflammation. (a) Venn diagram representing the distribution of differentially expressed genes in human microglia C20 cells, activated with either TNFα or TNFα/nEVs (red) compared to non‐activated or nEVs stimulated C20 cells (green). Genes involved in cell death and survival, cellular function and maintenance pathways are represented in the bar graphs. (b) Venn diagram representing the distribution of differentially expressed genes in C20 cells, activated with either TNFα or TNFα/aEVs (red) compared to non‐activated or aEVs stimulated C20 cells (green). Genes involved in cell death and survival, cellular function and maintenance pathways are represented in the bar graphs

Meta‐analysis of differentially expressed genes in TNFα‐activated or non‐activated microglia showed 20 genes (when stimulated with nEVs) and 26 genes (when stimulated with aEVs) exhibiting similar gene expression profiles (Figure 7a‐b). We compared the gene expression profile of key genes involved in neuroinflammation and cell death and survival signaling pathways. The results revealed similar gene expression patterns in the transcript abundance of genes regulating neuroinflammation signaling (C3, VEGFA, CDKN1A, FAS, Caspase 8, IL1β, CXCL8, MYC, IL6, TNFSF10: TRAIL) in both aEVs and nEVs stimulated cells with or without TNFα‐activation (Figure 7). We observed that caspase 1 gene expression was reduced only in non‐activated microglia stimulated with either nEVs or aEVs, but was slightly increased or remained unchanged in the presence of TNFα + M‐EVs stimulation (Figure 7a‐b). We detected some genes that either showed unique expression or no detected change in expression for one group compared to the respective control groups (FOS, MYD88, FADD, EPCAM, BCL2) (Figure 7a‐b). A complete differentially expressed gene list can be found in Tables S1 to S4. The transcriptome profile of the two subsets of M‐EVs cultured cells, exhibited both relatively similar but also unique differentially expressed genes in the expression profile, although for most genes involved in neuroinflammation, cell death and survival, a similar expression pattern was observed in nEVs and aEVs. Collectively, the results suggest that M‐EVs are involved in modulating neuroinflammation and cell death and survival signaling pathways in microglia.

### 3.7 Upstream regulator prediction analysis of modified target genes

**FIGURE 8:**
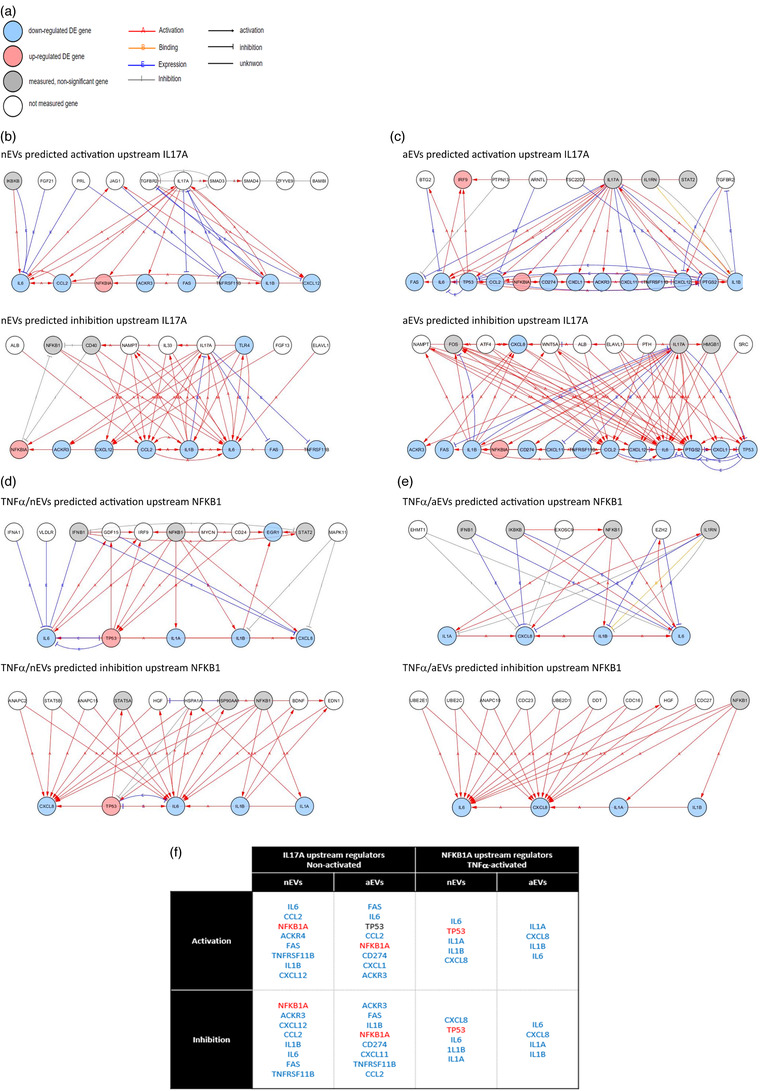
Network analysis showing upstream regulators predicted to affect differentially expressed genes in M‐EVs exposed cells. (a) Network analysis legend (b,c) Upstream transcriptional regulators of IL17A predicted as activated or inhibited in nEVs or aEVs stimulated human microglia C20 cells, in the absence of inflammation. (d,e) Upstream transcriptional regulators of NFKB1 predicted as activated or inhibited in nEVs or aEVs stimulated human microglia C20 cells in the presence of TNFα‐induced inflammation. (f) Overview table highlighting similarities and differences upstream of IL17A and NFKB1 gene activation or inhibition in either TNFα−activated or non‐activated microglia stimulated with either nEVs or aEVs

In order to further unravel the signaling pathways and biological activities occurring in microglia cells stimulated with M‐EVs, we performed an upstream regulator analysis in an attempt to identify the cascade that could explain the observed gene expression changes. The predicted upstream regulator analysis is based on the data of the differentially expressed genes from our RNA sequencing results that we analysed using iPathwayGuide software. iPathwayGuide predicts activation or inhibition of each regulator, based on the number of differentially expressed genes whose fold changes are consistent with the regulator predicted activity. This regulator can either be activated or inhibited, and the lines are signs of interaction between the regulator and target genes. Considering the role of M‐EVs in cell‐to‐cell signaling and interaction we should be able to extend these results and predict, by means of knowledge database sources, the transcriptional regulators and their potential target genes involved in the respective pathways and demonstrate how these upstream molecules may regulate one another. One of the canonical pathways that was significantly deregulated in our RNA sequencing data is the IL17 pathway in inflammation. Upstream regulator analysis of IL17A in non‐activated microglia stimulated with either nEVs or aEVs, both predicted activation and inhibition of key target genes including upregulation of NFKB1A and downregulation of IL6, IL1β, FAS, CXCL12, CCL2, ACKR3 targets genes (Figure 8b,c,f, upstream regulator IL17A). Of note is the observation that NFKB1 was predicted to be upregulated in both nEVs or aEVs stimulated cells. Activation of NFKB signaling pathways in microglia is known to result in the production of inflammatory mediators that can promote neuronal cell death (Dresselhaus & Meffert, 2019). Consequently, upstream regulator analysis of NFKB1 in TNFα‐activated microglia stimulated with either nEVs or aEVs, both predicted downregulation of key target genes including of IL6, IL1β, IL1a, CXCL8 (Figure 8d,e,f, upstream regulator NFKB1). Interestingly, NFKB1 upstream regulator analysis in the TNFα‐activated microglia, stimulated with nEVs, revealed in either the activation or inhibition state, an upregulation of the TP53 target gene (Figure 8d,f). Reports have shown that activation of TP53 induces expression of microRNAs that support microglia pro‐inflammatory functions and suppress anti‐inflammatory and tissue repair behaviours (Aloi, Su, & Garden, 2015). This impact analysis lends further support to our observation that microglial EVs are multi‐target molecular regulators capable of modulating protective activities towards survival and maintenance of homeostatic and repair functions of microglia.

### 3.8 Microglia EV‐stimulated cells do not promote cell death in vitro

**FIGURE 9:**
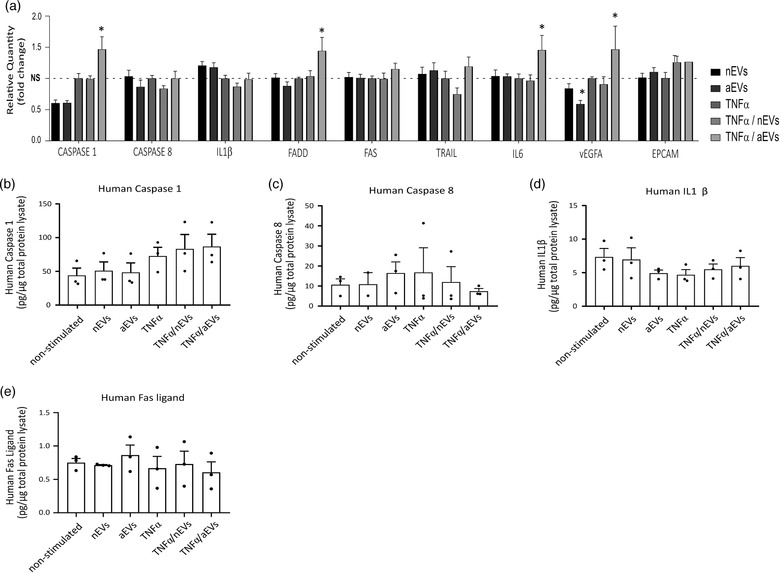
Quantification of key inflammatory and apoptotic markers that were not significantly upregulated in M‐EVs exposed cells. (a) Relative qPCR expression of candidate genes: caspase1, caspase8, IL1β, FADD, FAS, TRAIL, IL6, VEGFA and EPCAM in human microglia C20 cell lines stimulated with nEVs or aEVs either non‐activated or TNFα‐activated. Data were analysed using two stable reference genes and the fold changes were plotted as the mean ± SEM and normalized to non‐stimulated control cells (n = 3 biological replicates per group). (b‐e) ELISA analysis of apoptosis related markers: caspase 1, caspase 8, FAS and IL1β in microglia cell lysate stimulated with M‐EVs either non‐activated or TNFα‐activated. Values are represented as the mean ± SEM of three independent biological replicates. One‐way ANOVA Dunnett's multiple comparison test was used in GraphPad to determine statistical significance where P  < 0.05 was considered as statistically significant

To further validate some genes involved in the neuroinflammation, cell death and survival signaling pathways and confirm that M‐EVs stimulation of microglia does not significantly induce cell death, we performed several cell‐based assays. We measured the expression of genes involved in the cell death and survival pathways including IL‐1β, IL‐6, caspase 1, caspase 8, FADD, FAS, TNFSF10 (TRAIL), VEGFA and EPCAM in cells stimulated with nEVs or aEVs either in TNFα‐activated cells or non‐activated cells using quantitative PCR (qPCR) analysis. We observed a slight reduction or no significant change in gene expression of all the genes tested in non‐activated cells stimulated with either nEVs or aEVs (Figure 9a). In addition, we detected a slight but significant increase in caspase 1, FADD, IL6, VEGFA gene expression in TNFα‐activated cells stimulated with only aEVs. Furthermore, we performed ELISA analysis to further quantify the abundance of inflammatory and apoptotic proteins induced upon stimulation of cells with M‐EVs. Microglia EVs did not induce a significant increase in FAS, IL‐1β, caspase 1 or caspase 8 protein levels when compared to control cells in either the non‐activated or TNFα‐activated microglia (Figure 9b‐e). Taken together, the results show that stimulation of microglia EVs on cells did not induce major cell death activities *in vitro* and indicate that the slight upregulation detected in the TNFα + aEVs group could be involved in the maintenance of homeostatic and repair functions of microglia.

### 3.9 Membrane phosphatidylserine exposure and mitochondrial membrane potential are not impaired in microglial EVs stimulated cells

We have shown that M‐EVs stimulation of cells is able to modify key pro‐apoptotic gene expression levels including caspase 1 and caspase 8 transcripts. RNA sequencing data and RT‐PCR both showed that M‐EVs can modulate caspase 1 and caspase 8 expression levels in microglia cells (Figures 7, 8). Caspase 1, formerly called IL‐1β converting enzyme (ICE), is a cysteine protease that is involved in mediating programmed cell death by promoting the cleavage of critical intracellular proteins upon apoptotic activation. In order to determine whether cleaved forms of caspase 1 and caspase 8 proteins are excessively increased after stimulating cells with M‐EVs subsets, we performed immunoblotting and demonstrated a slight but non‐significant increase in the cleaved form of caspase 1 protein in cells stimulated with both nEVs and aEVs when compared to non‐stimulated control cells (Figure S2D). However, upon TNFα‐activation, immunoblotting analysis showed no significant difference in the cleaved caspase 1 form between M‐EVs + TNFα‐activated cells and TNFα only‐activated control cells (Supplementary figure S2E). The same experiment was performed for cleaved caspase 8 protein, yielding a similar trend of a slight but non‐significant increase in the cleavage form of caspase 8 protein when compared with the non‐activated control cells (Figure S2F). In the presence of TNFα‐activation, M‐EVs did not enhance the cleavage of caspase 8 protein (Figure S2G). In addition, immunoblot analysis using cleaved caspase 3 antibody did not reveal any cleaved form of caspase 3 protein (Figure S2H‐I).

**FIGURE 10:**
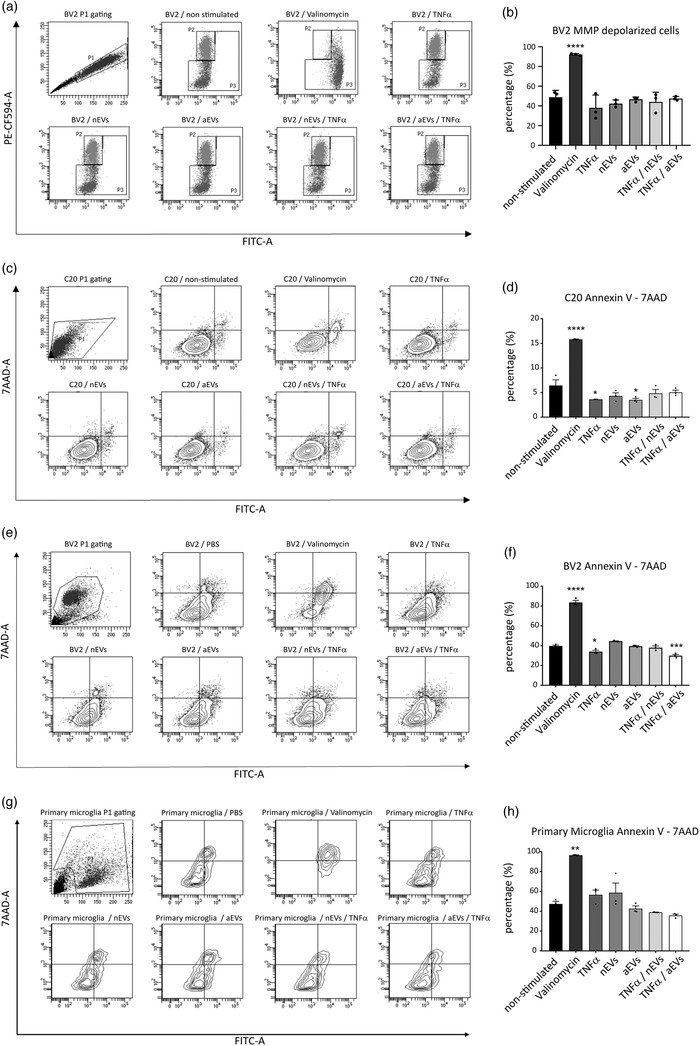
M‐EVs do not elicit apoptosis in microglia cells in vitro. (a,b) Quantification of JC‐1 mitochondrial membrane potential in human microglia C20 cells and Annexin V for the translocation of membrane phosphatidylserine in human microglia C20 cells (c,d) mouse microglia BV2 cells (e,f) and primary microglia (g,h). As a positive control, 1 μM of valinomycin was used to trigger depolarization of the mitochondrial membrane potential and to induce PS exposure. Data are shown as mean ± SEM (n = 3 biological replicates per group, 10,000 events per experiment). GraphPad was used to perform a one‐way ANOVA Dunnett's multiple comparison test to determine significance (*P < 0.05, **P < 0.01, ***P < 0.001, ****P < 0.0001).

Next, we sought to determine if the observed slight increase in cleaved caspase 1 and caspase 8 proteins could elicit changes in mitochondrial membrane potential indicative of early signs of apoptosis. We used the JC‐1 dye to monitor mitochondrial health and performed apoptosis studies in M‐EVs stimulated cells by flow cytometry. Our results show that microglia cells stimulated with either nEVs or aEVs did not elicit defects in mitochondria membrane potential when compared to non‐stimulated cells and valinomycin‐treated positive controls (Figure 10a,b). Another earlier sign of apoptosis is translocation of membrane phosphatidylserine from the inner side of the plasma membrane to the surface. We used Annexin V, a Ca^2+^‐dependent phospholipid‐binding protein with high affinity for phosphatidylserine, for the detection of exposed phosphatidylserine in M‐EVs stimulated cells by flow cytometry. In line with previous data, our results suggest that C20 microglia cells stimulated with either nEVs or aEVs showed a reduction in phosphatidylserine when compared to non‐stimulated cells (Figure 10c,d). Notably, there was a significant reduction in TNFα‐only and aEVs stimulated C20 cells (Figure 10d). In BV2 microglia cells stimulated with nEVs or aEVs, a similar trend was observed in the overall reduction of Annexin V positive cells (Figure 10e,f). In particular, there was a significant reduction in Annexin V positive cells observed in the TNFα only activated group and in the TNFα + aEVs stimulated group (Figure 10f). The same Annexin V experiment was validated in primary microglia cells and results demonstrated a similar trend that M‐EVs do not activate apoptosis in primary microglia cells (Figure 10g,h). Overall, our results show that M‐EVs exposure to C20, BV2 and primary microglia cells does not elicit apoptosis in vitro.

## 4 DISCUSSION

Our study reveals that microglial derived EVs play a role in the modulation of microglia cell death and survival gene expression signaling by the activation of autophagy and the reduction of apoptotic cell death.

Specifically, we analysed the biological effects of small extracellular vesicles (EVs) produced *in vitro* on either non‐activated or TNFα‐activated microglia. We present evidence that microglial extracellular vesicles (M‐EVs) can be internalized by the BV2 donor, C20 recipient microglia cell lines or primary cells, demonstrating that EVs from microglia origins can be taken up into different microglia subtypes. Furthermore, elucidating microglial cell‐specific molecular functions during EV‐mediated cell‐to‐cell communication is important to understand how the signaling processes could contribute to diseases (Paolicelli et al., 2019).

Importantly, our study reveals an increase in LC3B‐II protein and autophagic flux in live microglia cells stimulated with either nEVs or aEVs. The increased autophagy activation was demonstrated in both microglia cell lines and primary microglia cells, either isolated by the shake‐off method or by the CD11b‐MACS method, exposed to both TNFα and M‐EVs subsets, as well as by cells exposed to chloroquine treatment. In C20 cells exposed to both serum deprivation and bafilomycin treatment, no significant LC3 increase was observed after M‐EVs stimulation. This observation could be explained because the maximum response had already been induced by serum deprivation and Baf treatment. Therefore, addition of M‐EVs could not influence this maximum effect. We confirmed the activation of autophagy by M‐EVs using ultrastructural electron microscopy analysis and detected membrane‐enclosed vesicles that had engulfed cellular components.

Understanding the biological and molecular effects of M‐EVs on cells can provide insights on how to modulate toxic microglial inflammatory responses occurring in chronic demyelinating and neurodegenerative diseases. Following a targeted RNA sequencing approach, we used the inflammation and immunity transcriptome gene panel sequencing to elucidate the biological activities occurring at the transcriptional level in C20 microglia exposed to both nEVs and aEVs. We demonstrated that M‐EVs stimulation on activated or non‐activated microglia can influence multiple gene expression profiles, modify upstream regulators predicted to activate or inhibit target genes, enrichment pathways, and specifically modulate genes involved in the inflammasome pathway including caspase 1, caspase 8 and IL1β in both normal and activated microglia. In addition, M‐EVs stimulated cells showed significant downregulation of key apoptotic and inflammatory genes including death receptor FAS, TNFSF10 (TRAIL), CXCL8, caspase 8, IL‐6, and IL‐1β transcripts. These results support the notion that M‐EVs play a beneficial role in modulating protective activities of microglia cells upon inflammatory stress. Upstream regulator analysis lends further support to our observation that microglial EVs are multi‐target molecular mediators capable of activating cell survival signaling to maintain microglia homeostasis.

Recent studies have reported a critical role of EVs originating from neurons in regulating microglial activities (Kamentsky et al., 2011; Levine & Kroemer, 2008; Rong et al., 2019), by upregulating the complement molecule C3 to facilitate the removal of degenerating neurites by microglia (Levine & Kroemer, 2008; Rong et al., 2019). Similar to these earlier publications, we observed in our RNA sequencing study upregulation of the complement molecule C3 and MYD88 in M‐EVs stimulated cells, which could facilitate M‐EVs mediated autophagy. In this study, transcriptome analysis did not reveal obvious distinct neuroinflammation and cell death and survival signaling pathways between the two subsets of M‐EVs used to stimulate target C20 human microglia. The cell death and survival differentially expressed gene patterns exhibited by nEVs and aEVs showed only minor differences. Of note is that the aEVs used in this study were produced with a mild stimulation of only 10 ng/ ml TNFα for 24 h. We chose a low dose, which is optimal for inducing pro‐inflammatory responses without inducing toxicity. We opted for a dose of 10 ng/ml TNFα, which has previously been validated by our research group as an optimal dose to produce sufficient amounts of activated EVs without inducing cell death (Hosseinkhani et al., 2017). However, inflammatory EVs used in a more recent published report were produced from cells stimulated with a cocktail of 3 different Th1 cytokines, (IL1β, + TNFα + IFNɣ) for 48 h (Lombardi et al., 2019). Differences in readout could be observed when cells are stimulated with a higher concentration of inflammatory cytokines with a longer exposure time. Of note, we validated our key findings in primary cells showing that M‐EVs as well as the low dose of TNFα is sufficient to activate autophagy without inducing cell death.

EVs mediated cellular crosstalk is vital for a range of biological functions and for the maintenance of protein homeostasis within the CNS. Our study showed that *in vitro* produced M‐EVs significantly affected microglia cellular movement, cell death and survival as well as cellular growth and proliferation functions. Furthermore, we showed that M‐EVs did not trigger early signs of apoptosis neither in their mitochondrial membrane potential nor in the early exposure of phosphatidylserine at the outer surface of the plasma membrane. The link between autophagy and apoptosis in microglia could be influenced by M‐EVs ability to activate autophagy as a protective response during early cellular stress. Hence, microglial EVs are multimodal signaling mediators capable of influencing different neural cell types, and could be exploited to develop novel approaches for myelin repair, not only in multiple sclerosis but also in other demyelinating and neurological diseases (Lombardi et al., 2019). To our knowledge, we demonstrated for the first time that BV2 cell culture‐isolated M‐EVs can activate autophagy in target acceptor C20 human and in mouse primary microglia cells. M‐EVs ability to activate autophagy in cells is important upon neuroinflammation since toxic proteins and dying cells can be removed to prevent the accumulation of toxic molecules and to promote CNS homeostasis (Sierra, Abiega, Shahraz, & Neumann, 2013). Of note, it is reported that in human mesial temporal lobe epilepsy, inability of microglia to phagocytose dying cells due to the hyperactivity of the neuronal network, resulted in delayed cell clearance and neuroinflammation (Abiega et al., 2016).

In summary, we provide the perspective that a beneficial activity of *in vitro* cell culture produced EVs could be the activation of autophagy during cellular stress. This study demonstrates that during microglia stress, BV2‐derived M‐EVs can effectively incorporate into primary cells and into C20 human microglia and modulate the cell transcriptome profile, to activate autophagy and attenuate apoptosis and inflammatory immunomodulatory signaling. We used a monoculture system to study microglia‐microglia crosstalk which is important in the prevention and propagation of inflammation in the brain. This can have an important impact on lesion progression and repair. In particular, we found that M‐EVs can modulate the abundance of neuroinflammation and cell death and survival gene expression transcripts to maintain protein homeostasis upon microglia stress. Taken together, our findings provide the basis for the discovery of novel EVs mediated biological pathways and suggest that *in vitro* EVs may be used as promising new tools to develop cell type‐specific therapeutic approaches modulating neuroinflammatory diseases.

## CONFLICTS OF INTEREST

The authors declare that the research was conducted in the absence of any commercial or financial relationships that could be construed as a potential conflict of interest. The authors declare that they have no conflict of interest.

## AUTHOR CONTRIBUTIONS

Bram Van den Broek, Isabel Pintelon, and Joy Irobi designed and performed the experiments. Bram Van den Broek, Isabel Pintelon, Jean‐Pierre Timmermans, Ibrahim Hamad and Joy Irobi analyzed the data. Bram Van den Broek, and Joy Irobi wrote the manuscript. Sofie Kessels, Bert Brône, Ibrahim Hamad, Mansour Haidar, Markus Kleinewietfeld, Vincent Timmerman and Isabel Pintelon provided experimental materials. All authors revised the manuscript. Veerle Somers, Jerome J.A. Hendriks, Luc Michiels and Niels Hellings participated in the coordination of the project.

## Supporting information




**Supplementary figure 1: Phenotyping of primary microglia and autophagy flux assay. (S1A)** . qPCR was performed using the shake‐off and CD11b‐MACS methods for microglial genes (CD11b, HexB, Iba1, and Cx3cr1) and non‐microglial genes (NeuN, GFAP) in comparison with mouse brain. Graphpad was used to perform a one‐way ANOVA multiple comparison test or a student t‐test to determine significance (*** = p < 0.001, **** = p < 0.0001). **(S1B)** *Isolation of high purity microglia as verified by immunocytochemistry analysis. Representative image showing: microglia marker (Iba‐1, red), astrocyte marker (GFAP, green) and the DAPI nucleus stain (blue) (scale bar 100μm)*. **(S1C‐S1D)** Flow cytometry monitoring of CYTO‐ID autophagic flux in primary cells isolated by CD11b‐MACS microbeads using the CYTO‐ID green detection dye. Data are normalized to non‐stimulated control = 1000 MFI and shown as mean ± SEM (n = 3 biological replicates per group). Graphpad was used to perform a one‐way ANOVA to determine significance (* = p < 0.05, ** = p<0.01, *** = p < 0.001).

**Supplementary figure 2: TNFα proliferation assay and western blotting of key apoptotic markers caspase 1, caspase 8 and caspase 3. (S2A‐S2C)** *TNFα proliferation experiment showing total numbers of cells* ***(S2A)*** *and cell viability* ***(S2B)*** *from which EVs containing medium was collected to determine M‐EVs concentrations using nanoparticle tracking analysis* ***(S2C)***. Graphpad was used to perform a student t‐test to determine significance (** = p<0.01) **. (S2D‐S2I)** *Western blotting analysis of cleaved caspase 1* ***(S2D‐S2E)*,** *caspase 8* ***(S2F‐S2G)*,** *caspase 3* ***(S2H‐S2I)*** *in microglia C20 cells stimulated with nEVs or aEVs. Cells were either non‐activated or TNFα‐activated. The levels of cleaved fractions were calculated from 3 biological replicates and normalized to β−actin. Band intensities were determined by quantifying the mean pixel gray values using the ImageJ software. Flow cytometry was performed to determine the percentage of early signs of apoptosis in nEVs or aEVs stimulated cells, either TNFα‐activated or non‐activated. Graphpad was used to perform a one‐way ANOVA Dunnett's multiple comparison test to determine significance*.

Supplementary table 1: differential expressed gene counts tables from nEVs stimulated cells compared to non‐stimulated control cells.

Supplementary table 2: differential expressed gene counts tables from aEVs stimulated cells compared to non‐stimulated control cells.

Supplementary table 3: differential expressed gene counts tables from nEVs + TNFα‐stimulated cells compared to TNFα only‐stimulated cells.

Supplementary table 4: differential expressed gene counts tables from aEVs + TNFα‐stimulated cells compared to control TNFα only‐stimulated cells.

Supplementary table 5: qPCR primer sequences.

## Data Availability

The datasets used and/or analyzed during the current study are available from the corresponding author on reasonable request.

## Appendix

The human Inflammation & Immunity Transcriptome QIAseq targeted RNA panel contains cytokines, chemokines, growth factors and their receptors, downstream signaling pathway genes and transcription factors involved in coordinating diverse functions of the immune system. Genes with pro‐ or anti‐apoptotic effects in immune or targeted host cells are also included. Many of these genes are acutely upregulated due to cell damage, injury or infection to promote and eventually resolve the inflammatory response. Chronic inflammation, the expression of these cytokines and receptors at low levels over long periods of time, promotes various pathological conditions. More information on the QIAseq Targeted RNA Panels can be found on the link: https://b2b.qiagen.com/us/shop/sample-technologies/qiaseq-targeted-rna-panels/?catno = RHS‐005Z
