# Supplementary figures and images for: Microglial derived extracellular vesicles activate autophagy and mediate multi‐target signaling to maintain cellular homeostasis
Source: J Extracell Vesicles. 2020 Nov 25;10(1):e12022. doi: 10.1002/jev2.12022 (PMC7890546; doi:10.1002/jev2.12022)

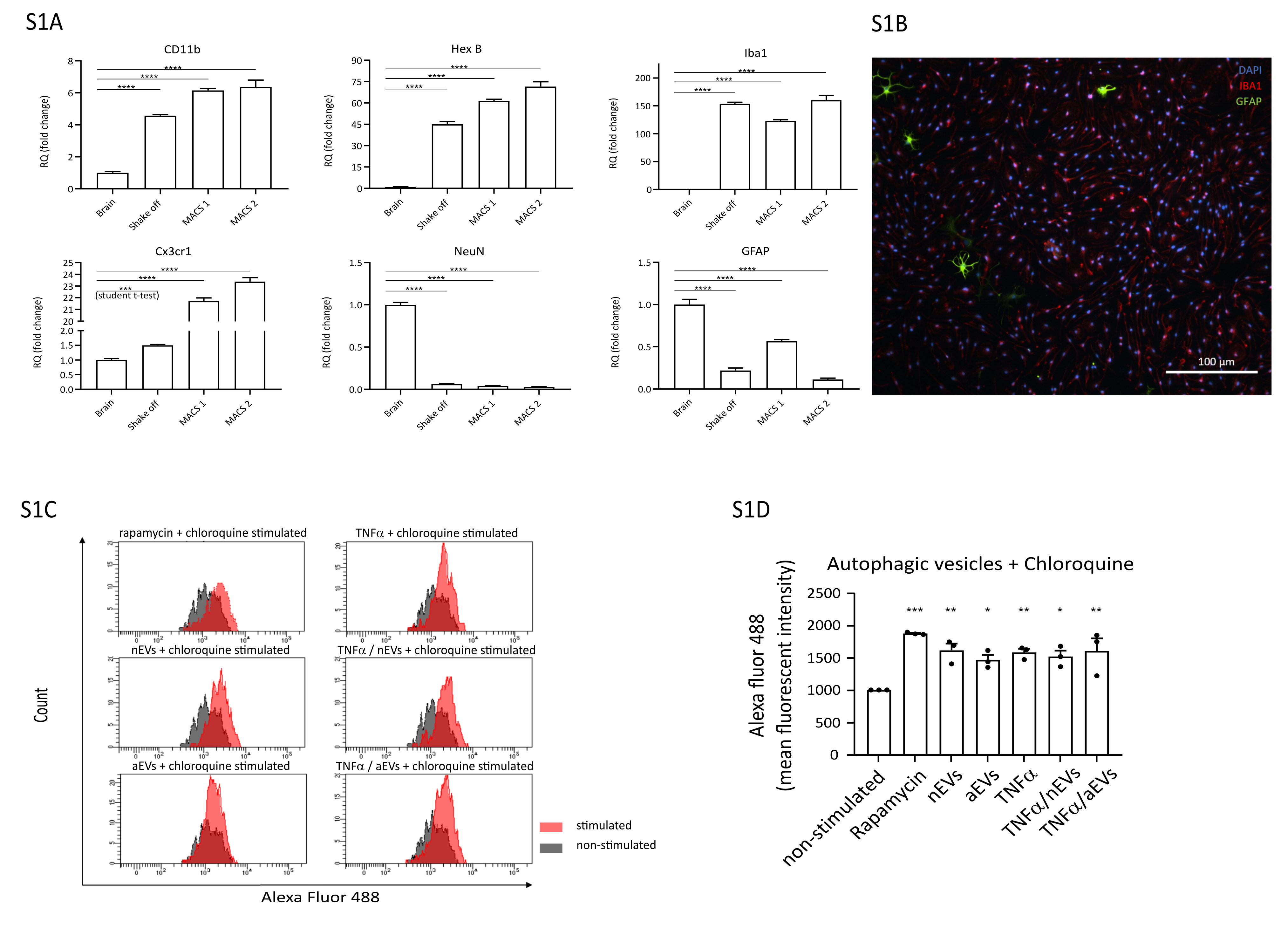

Supplement: Supplementary file 1 — Supplementary figure 1: Phenotyping of primary microglia and autophagy flux assay. (S1A) . qPCR was performed using the shake‐off and CD11b‐MACS methods for microglial genes (CD11b, HexB, Iba1, and Cx3cr1) and non‐microglial genes (NeuN, GFAP) in comparison with mouse brain. Graphpad was used to perform a one‐way ANOVA multiple comparison test or a student t‐test to determine significance (*** = p < 0.001, **** = p < 0.0001). (S1B) Isolation of high purity microglia as verified by immunocytochemistry analysis. Representative image showing: microglia marker (Iba‐1, red), astrocyte marker (GFAP, green) and the DAPI nucleus stain (blue) (scale bar 100μm). (S1C‐S1D) Flow cytometry monitoring of CYTO‐ID autophagic flux in primary cells isolated by CD11b‐MACS microbeads using the CYTO‐ID green detection dye. Data are normalized to non‐stimulated control = 1000 MFI and shown as mean ± SEM (n = 3 biological replicates per group). Graphpad was used to perform a one‐way ANOVA to determine significance (* = p < 0.05, ** = p<0.01, *** = p < 0.001). [file JEV2-10-e12022-s001.tiff]

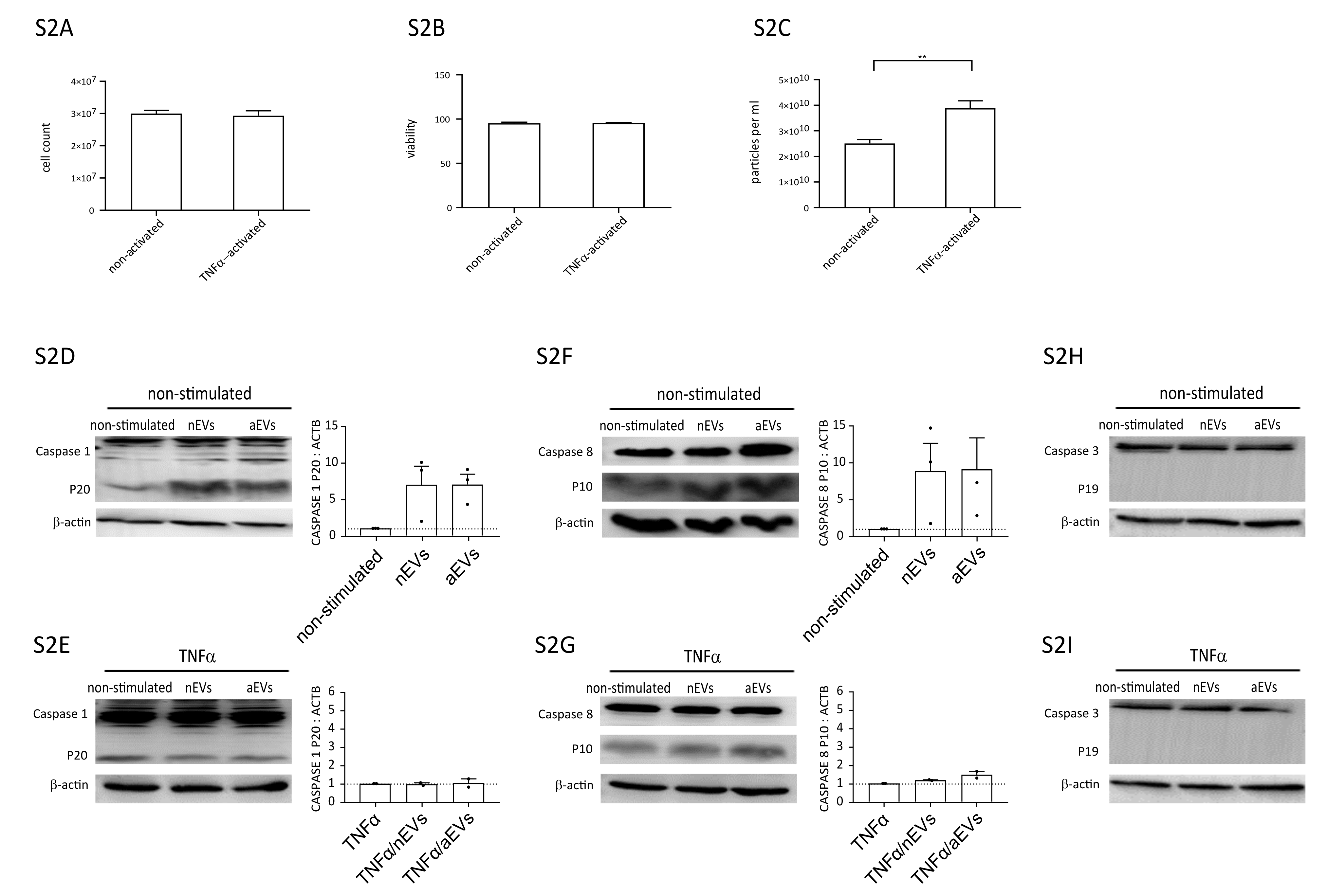

Supplement: Supplementary file 2 — Supplementary figure 2: TNFα proliferation assay and western blotting of key apoptotic markers caspase 1, caspase 8 and caspase 3. (S2A‐S2C) TNFα proliferation experiment showing total numbers of cells (S2A) and cell viability (S2B) from which EVs containing medium was collected to determine M‐EVs concentrations using nanoparticle tracking analysis (S2C). Graphpad was used to perform a student t‐test to determine significance (** = p<0.01) . (S2D‐S2I) Western blotting analysis of cleaved caspase 1 (S2D‐S2E), caspase 8 (S2F‐S2G), caspase 3 (S2H‐S2I) in microglia C20 cells stimulated with nEVs or aEVs. Cells were either non‐activated or TNFα‐activated. The levels of cleaved fractions were calculated from 3 biological replicates and normalized to β−actin. Band intensities were determined by quantifying the mean pixel gray values using the ImageJ software. Flow cytometry was performed to determine the percentage of early signs of apoptosis in nEVs or aEVs stimulated cells, either TNFα‐activated or non‐activated. Graphpad was used to perform a one‐way ANOVA Dunnett's multiple comparison test to determine significance. [file JEV2-10-e12022-s002.tiff]
