# Supplementary material for: Microglial derived extracellular vesicles activate autophagy and mediate multi‐target signaling to maintain cellular homeostasis
Source: J Extracell Vesicles. 2020 Nov 25;10(1):e12022. doi: 10.1002/jev2.12022 (PMC7890546; doi:10.1002/jev2.12022)
Supplement: Supplementary file 3 — Supplementary table 1: differential expressed gene counts tables from nEVs stimulated cells compared to non‐stimulated control cells. [file JEV2-10-e12022-s003.pdf]

Supplementary table 1 (non-stimulated control C20 cells vs nEVs stimulated C20 cells)

ensembl	hgnc_symbol	log2FoldChange	pvalue	padj
ENSG00000144648	ACKR2	0.692886925	0.109605621	0.190432112
ENSG00000144476	ACKR3	-1.479720806	6.15182E-09	5.03478E-08
ENSG00000129048	ACKR4	-0.527979542	0.200037202	0.304958675
ENSG00000196839	ADA	0.172215639	0.398544828	0.516447673
ENSG00000160710	ADAR	0.566390539	9.55901E-05	0.000443709
ENSG00000164022	AIMP1	0.379584556	0.0076016	0.021108014
ENSG00000164111	ANXA5	-1.16062574	3.32285E-32	2.58352E-30
ENSG00000115307	AUP1	-0.484378361	0.002175294	0.007047046
ENSG00000171791	BCL2	0.159699578	0.324333659	0.436657005
ENSG00000113916	BCL6	-0.23791345	0.099122079	0.177166475
ENSG00000095585	BLNK	0.808048654	0.033724574	0.075455702
ENSG00000125378	BMP4	-0.236209801	0.474740523	0.583574319
ENSG00000153162	BMP6	-0.576811425	0.066501894	0.127667215
ENSG00000130303	BST2	0.787801463	5.46163E-06	3.14549E-05
ENSG00000010671	BTK	1.108046107	0.008750842	0.024084176
ENSG00000125730	C3	1.631709841	2.36789E-27	1.47283E-25
ENSG00000171860	C3AR1	-0.889956217	0.031821315	0.073306881
ENSG00000106804	C5	0.23884258	0.282354605	0.39377705
ENSG00000197405	C5AR1	0.456985658	0.174049454	0.273380708
ENSG00000137752	CASP1	-0.329773963	0.05119101	0.105433139
ENSG00000064012	CASP8	-0.245497073	0.1189049	0.200975131
ENSG00000105974	CAV1	-0.827802601	3.73242E-13	5.10817E-12
ENSG00000114423	CBLB	0.164384091	0.209337931	0.316039303
ENSG00000108691	CCL2	-0.699999835	0.000475365	0.001919979
ENSG00000115009	CCL20	0.304220544	0.414363214	0.530180604
ENSG00000102962	CCL22	1.17150231	0.003484491	0.010836768
ENSG00000006606	CCL26	0.083634859	0.818331808	0.874574544
ENSG00000163823	CCR1	-0.480727042	0.270333293	0.383897964
ENSG00000184451	CCR10	2.012467527	2.14043E-14	4.43782E-13
ENSG00000121797	CCRL2	-0.790984618	0.037590869	0.082913194
ENSG00000170458	CD14	0.913009935	1.39729E-06	9.05327E-06
ENSG00000134061	CD180	-1.902331168	7.51718E-06	4.10148E-05
ENSG00000120217	CD274	-0.153423902	0.442249045	0.552367282
ENSG00000103855	CD276	-0.323364823	0.101445644	0.180283401
ENSG00000010610	CD4	1.13216233	0.000293252	0.001262555
ENSG00000101017	CD40	0.735565545	0.08109549	0.149251241
ENSG00000026508	CD44	-1.360101871	1.0073E-16	2.84791E-15
ENSG00000196776	CD47	-0.165777394	0.137800109	0.224376093
ENSG00000135404	CD63	-0.922338364	3.02566E-07	2.09107E-06
ENSG00000019582	CD74	0.703594506	0.010560741	0.028313711
ENSG00000110651	CD81	-0.430473987	0.000571339	0.002193659
ENSG00000114013	CD86	0.901619351	0.009674151	0.026391761
ENSG00000010278	CD9	-0.111833831	0.622424518	0.709062363
ENSG00000237350	CDC42P6	-1.024154539	0.015293182	0.039307269
ENSG00000123374	CDK2	-0.356196508	0.033019774	0.074874339

ENSG00000124762	CDKN1A	1.3655918	1.81846E-11	2.0946E-10
ENSG00000111276	CDKN1B	0.431491889	0.000156438	0.00069503
ENSG00000172216	CEBPB	1.068702658	2.7039E-05	0.000131393
ENSG00000213341	CHUK	-0.114140988	0.412295481	0.529850804
ENSG00000179583	CIITA	-0.363302477	0.401170796	0.517693434
ENSG00000174600	CMKLR1	0.101276199	0.798537147	0.859325442
ENSG00000109846	CRYAB	0.897157756	1.39529E-09	1.35605E-08
ENSG00000184371	CSF1	0.612684268	1.03606E-06	7.0047E-06
ENSG00000182578	CSF1R	-0.619482438	0.118764355	0.200975131
ENSG00000164400	CSF2	-2.230981722	1.50307E-13	2.46028E-12
ENSG00000006210	CX3CL1	1.668827153	3.9637E-18	1.23271E-16
ENSG00000163739	CXCL1	-0.059917356	0.776303381	0.838299831
ENSG00000169245	CXCL10	-0.117981726	0.7286352	0.803565771
ENSG00000169248	CXCL11	-0.625919103	0.037905953	0.083019375
ENSG00000107562	CXCL12	-3.403511885	5.69751E-49	8.85962E-47
ENSG00000145824	CXCL14	-2.518002546	6.20887E-11	6.89628E-10
ENSG00000161921	CXCL16	1.051669767	4.33873E-09	3.74818E-08
ENSG00000081041	CXCL2	0.170991277	0.352614955	0.464674792
ENSG00000163734	CXCL3	0.806371425	0.054406742	0.110591482
ENSG00000163735	CXCL5	-0.00090492	0.994616243	0.994616243
ENSG00000124875	CXCL6	-0.262648547	0.321497806	0.434720946
ENSG00000169429	CXCL8	-1.292656328	7.78387E-09	6.20714E-08
ENSG00000121966	CXCR4	-0.368705621	0.056113253	0.112588527
ENSG00000107201	DDX58	0.353678085	0.00748123	0.020960924
ENSG00000149091	DGKZ	-0.319099956	0.169048257	0.268234734
ENSG00000179611	DGKZP1	-0.118268114	0.757882385	0.827022533
ENSG00000197635	DPP4	-0.342260564	0.132824476	0.217412695
ENSG00000105246	EBI3	0.264057048	0.277937309	0.389362626
ENSG00000138798	EGF	-0.365421354	0.228264302	0.334859424
ENSG00000146648	EGFR	0.215963079	0.149307847	0.239354331
ENSG00000120738	EGR1	-0.064695389	0.700952054	0.781347988
ENSG00000122877	EGR2	-0.219578176	0.300353113	0.412767383
ENSG00000179388	EGR3	-0.326057053	0.090420755	0.163493341
ENSG00000055332	EIF2AK2	0.864518113	1.19132E-13	2.05834E-12
ENSG00000126767	ELK1	-0.147421258	0.716527547	0.795857382
ENSG00000119888	EPCAM	1.449674751	5.65068E-09	4.74963E-08
ENSG00000141736	ERBB2	0.393072687	0.044879758	0.095123776
ENSG00000117525	F3	-1.946047023	3.70748E-38	3.84342E-36
ENSG00000026103	FAS	-0.615153121	0.000524526	0.002064907
ENSG00000112787	FBRSL1	0.825264725	1.01698E-09	1.0843E-08
ENSG00000137312	FLOT1	-0.415971438	0.336918776	0.445879742
ENSG00000170345	FOS	0.639440452	0.001777284	0.006007994
ENSG00000175592	FOSL1	-0.985814437	1.00141E-18	3.67819E-17
ENSG00000114861	FOXP1	0.625100915	0.001845428	0.006171271
ENSG00000049768	FOXP3	-0.669279063	0.118318172	0.200975131
ENSG00000107485	GATA3	-0.801433952	0.002726039	0.008651
ENSG00000117228	GBP1	-0.134665571	0.446671211	0.555658986
ENSG00000162676	GFI1	-0.779851784	0.05945483	0.117773581
ENSG00000105220	GPI	-0.031435517	0.933022776	0.94211066
ENSG00000094631	HDAC6	0.123909121	0.509814492	0.612171069

ENSG00000048052	HDAC9	-1.136385683	6.15644E-06	3.41902E-05
ENSG00000100644	HIF1A	0.118004736	0.294243684	0.406710159
ENSG00000203812	HIST2H2AA3	-0.045871938	0.91583147	0.930796037
ENSG00000272196	HIST2H2AA4	-0.726541877	0.000296355	0.001262555
ENSG00000184260	HIST2H2AC	-0.399425466	0.318884177	0.433069778
ENSG00000206503	HLA-A	-0.923270459	0.002962858	0.009307564
ENSG00000234745	HLA-B	0.021570932	0.960320384	0.963418192
ENSG00000204525	HLA-C	0.349968441	0.184520443	0.286929289
ENSG00000204632	HLA-G	0.150504525	0.684063252	0.768027695
ENSG00000189403	HMGB1	-0.114602098	0.583521025	0.677145667
ENSG00000080824	HSP90AA1	0.134359008	0.479366664	0.586941073
ENSG00000106211	HSPB1	-0.344014459	0.14056723	0.226509888
ENSG00000152137	HSPB8	-0.862226167	0.047930366	0.100042576
ENSG00000144381	HSPD1	0.593335091	0.004461486	0.013214497
ENSG00000090339	ICAM1	0.401685339	0.004516288	0.013250618
ENSG00000115738	ID2	-0.727861326	6.94494E-05	0.000332289
ENSG00000131203	IDO1	-0.698154594	0.02915189	0.0681672
ENSG00000163565	IFI16	0.916055007	4.66994E-08	3.37756E-07
ENSG00000165949	IFI27	-0.02443584	0.942096569	0.948194282
ENSG00000137965	IFI44	0.426620719	0.002574493	0.008254301
ENSG00000137959	IFI44L	0.383510156	0.014505647	0.037593801
ENSG00000126709	IFI6	-0.29108059	0.00441367	0.013198572
ENSG00000115267	IFIH1	0.437823645	0.010758167	0.028596496
ENSG00000185745	IFIT1	0.770458989	3.09798E-09	2.75277E-08
ENSG00000119922	IFIT2	0.304401961	0.026416674	0.062714395
ENSG00000119917	IFIT3	-0.633182334	1.56915E-05	8.00007E-05
ENSG00000185885	IFITM1	0.220861631	0.609119673	0.696456685
ENSG00000185201	IFITM2	1.355800903	8.82745E-21	4.57556E-19
ENSG00000142089	IFITM3	0.62404233	0.000342137	0.001418728
ENSG00000142166	IFNAR1	-0.244387885	0.025832697	0.061799761
ENSG00000171855	IFNB1	0.952366967	0.028802493	0.06786042
ENSG00000184995	IFNE	0.345084425	0.301280373	0.412767383
ENSG00000027697	IFNGR1	-0.912535647	1.30698E-12	1.69363E-11
ENSG00000159128	IFNGR2	-0.614797497	0.161601924	0.257734351
ENSG00000185436	IFNLR1	-0.054312843	0.843411046	0.892179712
ENSG00000104365	IKBKB	-0.350861841	0.125293112	0.208375176
ENSG00000095752	IL11	-0.026964541	0.894947743	0.921618371
ENSG00000137070	IL11RA	0.113319861	0.723335712	0.800560166
ENSG00000168811	IL12A	0.331472432	0.272808236	0.383906613
ENSG00000113302	IL12B	-0.495758359	0.243913744	0.352824067
ENSG00000081985	IL12RB2	-0.233321319	0.592047909	0.684486616
ENSG00000131724	IL13RA1	0.039470063	0.768860239	0.836068302
ENSG00000164136	IL15	0.114067781	0.635872495	0.719113985
ENSG00000177663	IL17RA	0.206032813	0.485914955	0.590789981
ENSG00000056736	IL17RB	0.067781767	0.848428845	0.894445325
ENSG00000163701	IL17RE	0.131596813	0.698977709	0.781347988
ENSG00000150782	IL18	0.292878262	0.488209084	0.590789981
ENSG00000115604	IL18R1	0.541467055	0.107963192	0.188632319
ENSG00000115008	IL1A	0.75498045	0.063723014	0.123861609
ENSG00000125538	IL1B	-1.14757139	1.6171E-05	8.11161E-05

ENSG00000115594	IL1R1	0.524573765	0.001163521	0.004207616
ENSG00000196083	IL1RAP	-0.228389419	0.113858101	0.196721497
ENSG00000174564	IL20RB	1.503668693	5.79872E-06	3.27891E-05
ENSG00000103522	IL21R	-0.542000018	0.21314272	0.320228918
ENSG00000110944	IL23A	0.62929833	0.038233143	0.083150402
ENSG00000104998	IL27RA	1.306857356	3.77775E-13	5.10817E-12
ENSG00000100385	IL2RB	1.019370233	0.018568865	0.045471789
ENSG00000164509	IL31RA	-1.188004906	0.001939633	0.006417297
ENSG00000113520	IL4	-0.571859068	0.189737049	0.293573245
ENSG00000077238	IL4R	-0.224210735	0.357959635	0.469727622
ENSG00000136244	IL6	-2.551572597	8.02137E-51	2.49464E-48
ENSG00000160712	IL6R	0.566748352	0.172479699	0.272290287
ENSG00000104432	IL7	-0.300569518	0.202591312	0.307345844
ENSG00000168685	IL7R	-0.582458899	0.000104302	0.000476843
ENSG00000123999	INHA	1.258074271	7.88651E-06	4.2288E-05
ENSG00000122641	INHBA	-0.984131147	0.017920655	0.04458659
ENSG00000184216	IRAK1	-0.055741798	0.878197029	0.910967239
ENSG00000134070	IRAK2	0.28122813	0.241289768	0.350659429
ENSG00000198001	IRAK4	-0.453806542	0.001406661	0.005028408
ENSG00000125347	IRF1	0.015028791	0.922859925	0.934884159
ENSG00000168310	IRF2	-0.058678801	0.602375198	0.693846987
ENSG00000170604	IRF2BP1	0.321185252	0.056751861	0.113139928
ENSG00000126456	IRF3	0.23778097	0.138997092	0.225146332
ENSG00000128604	IRF5	0.988375822	0.022442526	0.054105625
ENSG00000117595	IRF6	0.03310972	0.905313049	0.926159073
ENSG00000185507	IRF7	0.374682714	0.377122152	0.492794073
ENSG00000187608	ISG15	-0.643515141	1.28088E-07	9.05352E-07
ENSG00000172183	ISG20	0.060098736	0.732484836	0.804410224
ENSG00000078747	ITCH	0.15587005	0.250158825	0.358522556
ENSG00000213949	ITGA1	-0.43003216	0.010154019	0.027459999
ENSG00000005961	ITGA2B	0.796293027	0.047549982	0.09991922
ENSG00000162434	JAK1	0.374843085	0.00388594	0.011848308
ENSG00000096968	JAK2	0.126546143	0.549391419	0.647475795
ENSG00000105639	JAK3	1.480134605	0.000563255	0.002189652
ENSG00000177606	JUN	0.161338082	0.196190797	0.300568166
ENSG00000100578	KIAA0586	0.074862992	0.551707671	0.647475795
ENSG00000049130	KITLG	-0.58867115	2.60255E-05	0.000128475
ENSG00000089692	LAG3	0.742778477	0.069554408	0.13109952
ENSG00000116678	LEPR	0.683801428	0.000105795	0.000476843
ENSG00000131981	LGALS3	-0.031443294	0.878864901	0.910967239
ENSG00000128342	LIF	-1.875983866	1.98555E-09	1.87123E-08
ENSG00000123384	LRP1	0.823857114	3.3729E-06	2.05681E-05
ENSG00000154589	LY96	0.157642274	0.544571758	0.646419148
ENSG00000254087	LYN	-0.318444695	0.029689617	0.068906499
ENSG00000090382	LYZ	-0.805770113	0.055378116	0.111835026
ENSG00000178573	MAF	-1.456095751	1.06443E-18	3.67819E-17
ENSG00000034152	MAP2K3	-0.706244621	0.000594251	0.002253804
ENSG00000095015	MAP3K1	0.111614421	0.549833663	0.647475795
ENSG00000169967	MAP3K2	0.224691538	0.083264454	0.152324973
ENSG00000135341	MAP3K7	0.367310332	0.011434952	0.030137883

ENSG00000100030	MAPK1	-0.188096982	0.219274096	0.324734494
ENSG00000107643	MAPK8	-0.38806112	0.017644987	0.044254765
ENSG00000105976	MET	-0.113339436	0.415961632	0.530180604
ENSG00000130731	METTL26	-0.059610654	0.773703241	0.838299831
ENSG00000204520	MICA	-0.05873314	0.877041087	0.910967239
ENSG00000158411	MITD1	0.019537451	0.903754459	0.926159073
ENSG00000156738	MS4A1	2.47125268	2.04845E-09	1.87373E-08
ENSG00000196814	MVB12B	-0.041384587	0.839965587	0.891567569
ENSG00000157601	MX1	0.643088631	1.52228E-05	7.8905E-05
ENSG00000183486	MX2	0.471906513	9.36777E-05	0.000441421
ENSG00000136997	MYC	-2.213053291	6.5888E-14	1.20536E-12
ENSG00000172936	MYD88	0.602519592	0.000864078	0.003199146
ENSG00000158092	NCK1	0.333656111	0.062081405	0.121429666
ENSG00000184983	NDUFA6	-0.090766705	0.633511069	0.719058184
ENSG00000131196	NFATC1	0.779003914	0.044657415	0.095123776
ENSG00000101096	NFATC2	0.579622696	0.107567111	0.188632319
ENSG00000072736	NFATC3	0.222495332	0.097579712	0.175417863
ENSG00000109320	NFKB1	0.311377284	0.071388226	0.133745412
ENSG00000077150	NFKB2	0.595607892	0.004204345	0.012694672
ENSG00000100906	NFKBIA	0.937556193	1.82128E-06	1.13284E-05
ENSG00000170322	NFRKB	0.182582665	0.382110095	0.49722276
ENSG00000123609	NMI	0.459713946	0.03439914	0.076415233
ENSG00000106100	NOD1	-0.032849626	0.881675688	0.910967239
ENSG00000148400	NOTCH1	0.473632052	0.0169767	0.042924826
ENSG00000177463	NR2C2	0.175918222	0.219207598	0.324734494
ENSG00000113580	NR3C1	0.21595737	0.116653654	0.200438046
ENSG00000123358	NR4A1	-2.154000636	6.34225E-16	1.6437E-14
ENSG00000119508	NR4A3	-0.666465726	0.013303694	0.034768478
ENSG00000111335	OAS2	0.252248696	0.218831472	0.324734494
ENSG00000261371	PECAM1	-0.426638625	0.326176913	0.437245775
ENSG00000197329	PELI1	0.062117692	0.606121081	0.695585447
ENSG00000140464	PML	0.041637188	0.832863987	0.887057192
ENSG00000028277	POU2F2	-0.936439079	0.022173571	0.053874849
ENSG00000186951	PPARA	1.009235938	2.09154E-08	1.58651E-07
ENSG00000132170	PPARG	0.755752527	1.66065E-06	1.05401E-05
ENSG00000084072	PPIE	-0.599274378	3.81674E-06	2.23963E-05
ENSG00000100023	PPIL2	0.016680235	0.909050746	0.926933712
ENSG00000067606	PRKCZ	0.236042528	0.426021402	0.53424458
ENSG00000180228	PRKRA	0.019808704	0.874673985	0.910967239
ENSG00000092010	PSME1	-0.011693704	0.867625266	0.910967239
ENSG00000125384	PTGER2	0.927058439	0.001439031	0.005028524
ENSG00000073756	PTGS2	-0.611015728	0.053605604	0.109679888
ENSG00000111737	RAB35	-0.397026275	0.005668779	0.016476546
ENSG00000136238	RAC1	-0.442323222	0.001436911	0.005028524
ENSG00000162924	REL	0.648383251	3.55432E-06	2.12576E-05
ENSG00000173039	RELA	0.64927884	0.000490899	0.001957304
ENSG00000104856	RELB	1.047256814	2.06E-08	1.58651E-07
ENSG00000132005	RFX1	0.818155957	8.61821E-12	1.03087E-10
ENSG00000104312	RIPK2	0.228906511	0.124820194	0.208375176
ENSG00000133135	RNF128	-0.23615308	0.574928142	0.669672855

ENSG00000069667	RORA	0.508228044	0.03211553	0.07344066
ENSG00000143365	RORC	2.49786748	2.37321E-19	1.05438E-17
ENSG00000159216	RUNX1	-0.261792595	0.050776766	0.105277161
ENSG00000020633	RUNX3	0.703299268	0.068744589	0.130363214
ENSG00000170989	S1PR1	-0.405710958	0.271607509	0.383906613
ENSG00000188404	SELL	-0.828019078	0.041829859	0.090340877
ENSG00000133661	SFTPD	0.090154901	0.817446787	0.874574544
ENSG00000185187	SIGIRR	-0.214599634	0.421502988	0.533598895
ENSG00000145147	SLIT2	-0.343068512	0.310654993	0.42374431
ENSG00000185338	SOCS1	-1.502907346	1.2146E-09	1.21852E-08
ENSG00000184557	SOCS3	-0.10515417	0.423790762	0.533598895
ENSG00000171150	SOCS5	0.174768883	0.131329652	0.21610329
ENSG00000118785	SPP1	-1.878283357	1.10421E-06	7.30657E-06
ENSG00000115415	STAT1	0.263068434	0.067473458	0.128737701
ENSG00000170581	STAT2	0.492524625	0.000300708	0.001263785
ENSG00000168610	STAT3	0.107008955	0.540841055	0.644450452
ENSG00000138378	STAT4	0.406810211	0.292030431	0.405452964
ENSG00000126561	STAT5A	0.753326441	0.000208983	0.000915405
ENSG00000166888	STAT6	0.202776663	0.460910166	0.568821673
ENSG00000231925	TAPBP	-1.054242508	0.016647006	0.04243622
ENSG00000183735	TBK1	-0.208782238	0.065254943	0.126051473
ENSG00000073861	TBX21	-0.963750689	0.006645009	0.018959614
ENSG00000163235	TGFA	-0.439596932	0.224706448	0.331202394
ENSG00000105329	TGFB1	-0.879108264	0.000377495	0.001544751
ENSG00000092969	TGFB2	-0.221904699	0.090169239	0.163493341
ENSG00000119699	TGFB3	-1.150066121	0.000700426	0.002624486
ENSG00000041988	THAP3	-0.301460012	0.195632694	0.300568166
ENSG00000137801	THBS1	-1.827750568	2.79899E-12	3.48194E-11
ENSG00000127666	TICAM1	0.52116946	0.018379562	0.045365426
ENSG00000102265	TIMP1	-0.059350751	0.532051158	0.636415038
ENSG00000150455	TIRAP	-0.244836529	0.561910426	0.65697046
ENSG00000174125	TLR1	-0.135838101	0.660991276	0.744812633
ENSG00000137462	TLR2	-0.562596753	0.076869128	0.143151489
ENSG00000164342	TLR3	-0.181013795	0.329506209	0.439813009
ENSG00000136869	TLR4	-1.495879213	1.58293E-13	2.46146E-12
ENSG00000187554	TLR5	1.030042375	0.00175588	0.006000863
ENSG00000174130	TLR6	-1.230225084	0.000996609	0.003646418
ENSG00000184584	TMEM173	-0.454019953	0.001587974	0.005487334
ENSG00000104689	TNFRSF10A	-0.120362963	0.487096203	0.590789981
ENSG00000164761	TNFRSF11B	-1.243417703	3.73297E-15	8.93041E-14
ENSG00000157873	TNFRSF14	0.288794596	0.49843808	0.600830398
ENSG00000186891	TNFRSF18	-0.290198588	0.422169233	0.533598895
ENSG00000067182	TNFRSF1A	-0.467999682	0.005913552	0.017028838
ENSG00000120949	TNFRSF8	0.854797888	0.033223983	0.074874339
ENSG00000049249	TNFRSF9	0.933806521	2.8094E-08	2.0803E-07
ENSG00000121858	TNFSF10	-0.962584344	1.25144E-05	6.59659E-05
ENSG00000239697	TNFSF12	0.591217308	0.002145029	0.007022148
ENSG00000102524	TNFSF13B	-0.32438352	0.458916524	0.568617685
ENSG00000117586	TNFSF4	0.328276565	0.253980111	0.362329425
ENSG00000078902	TOLLIP	-0.261167424	0.12001449	0.201754088

ENSG00000141510	TP53	-0.386861787	0.006819729	0.019281233
ENSG00000164938	TP53INP1	-0.28344159	0.081104372	0.149251241
ENSG00000131323	TRAF3	0.416299318	0.003769299	0.011606455
ENSG00000175104	TRAF6	0.287884554	0.044962042	0.095123776
ENSG00000074319	TSG101	-0.859321715	1.85034E-14	4.11039E-13
ENSG00000084652	TXLNA	-0.283767842	0.128745662	0.212978196
ENSG00000105397	TYK2	-0.227632328	0.335384637	0.445746249
ENSG00000025708	TYMP	-0.128065453	0.734573967	0.804410224
ENSG00000177889	UBE2N	-1.267722915	5.32814E-14	1.03566E-12
ENSG00000162692	VCAM1	0.171822749	0.241262284	0.350659429
ENSG00000112715	VEGFA	1.378371242	1.04595E-09	1.0843E-08
ENSG00000167987	VPS37C	-0.32957866	0.105424187	0.18628933
ENSG00000160685	ZBTB7B	0.328826359	0.178417268	0.278833017
ENSG00000083838	ZNF446	0.331111976	0.061374881	0.120807519
ENSG00000089127		0.194879956	0.248652304	0.358013272
ENSG00000125726		-0.967465492	2.89987E-13	4.29457E-12
