# Supplementary material for: Microglial derived extracellular vesicles activate autophagy and mediate multi‐target signaling to maintain cellular homeostasis
Source: J Extracell Vesicles. 2020 Nov 25;10(1):e12022. doi: 10.1002/jev2.12022 (PMC7890546; doi:10.1002/jev2.12022)
Supplement: Supplementary file 4 — Supplementary table 2: differential expressed gene counts tables from aEVs stimulated cells compared to non‐stimulated control cells. [file JEV2-10-e12022-s004.pdf]

Supplementary table 2 (non-stimulated control C20 cells vs aEVs stimulated C20 cells)

ensembl	hgnc_symbol	log2FoldChange	pvalue	padj
ENSG00000144648	ACKR2	0.429440834	0.194675017	0.292930804
ENSG00000144476	ACKR3	-1.735488147	8.26235E-17	9.76182E-16
ENSG00000129048	ACKR4	-0.417722307	0.283586279	0.402062325
ENSG00000196839	ADA	-0.006550687	0.567786058	0.680916363
ENSG00000160710	ADAR	0.573047253	2.24188E-06	1.02166E-05
ENSG00000164022	AIMP1	0.238803301	0.006345117	0.015569942
ENSG00000164111	ANXA5	-1.17409444	1.37239E-92	3.34242E-90
ENSG00000115307	AUP1	-0.358242521	0.000565441	0.001768391
ENSG00000171791	BCL2	0.017447369	0.565439475	0.680661104
ENSG00000113916	BCL6	-0.391605359	0.002874464	0.007578133
ENSG00000095585	BLNK	0.2675578	0.067732302	0.124175888
ENSG00000125378	BMP4	-0.222545926	0.661764818	0.753939203
ENSG00000153162	BMP6	-0.698053494	0.033135002	0.067756832
ENSG00000130303	BST2	1.087884507	1.74809E-13	1.50714E-12
ENSG00000010671	BTK	0.686121863	0.011893739	0.028104466
ENSG00000125730	C3	1.395743236	1.91192E-33	6.7767E-32
ENSG00000171860	C3AR1	-0.866901889	0.024293538	0.052718631
ENSG00000106804	C5	0.34741468	0.176646238	0.272222946
ENSG00000197405	C5AR1	0.777800336	0.025509769	0.054614874
ENSG00000137752	CASP1	-0.449456108	0.004410433	0.011438439
ENSG00000064012	CASP8	-0.365604487	0.014820888	0.034013404
ENSG00000105974	CAV1	-0.874404037	7.08181E-33	2.2591E-31
ENSG00000114423	CBLB	0.148814215	0.336904358	0.463243492
ENSG00000108691	CCL2	-0.858125913	4.71939E-07	2.24699E-06
ENSG00000115009	CCL20	0.255338246	0.636087552	0.732061734
ENSG00000102962	CCL22	-0.729922226	0.000499087	0.001592088
ENSG00000131142	CCL25	0.375214793	0.080227514	0.142180984
ENSG00000006606	CCL26	0.28775854	0.754996457	0.819145235
ENSG00000163823	CCR1	-0.312501331	0.446064959	0.566816586
ENSG00000184451	CCR10	1.736271198	1.21909E-17	1.55556E-16
ENSG00000121797	CCRL2	-0.37306307	0.062082674	0.115941652
ENSG00000170458	CD14	0.744733323	3.10286E-07	1.49972E-06
ENSG00000134061	CD180	-1.904466648	1.05136E-08	5.88391E-08
ENSG00000120217	CD274	-0.383626491	0.105436	0.179861411
ENSG00000103855	CD276	-0.474879549	0.013000079	0.030270257
ENSG00000010610	CD4	1.107204908	0.000113253	0.000392692
ENSG00000101017	CD40	0.027862557	0.167289248	0.261594462
ENSG00000026508	CD44	-1.419602773	1.22901E-33	4.90068E-32
ENSG00000196776	CD47	-0.234924869	0.018932218	0.041855719
ENSG00000135404	CD63	-0.923266651	9.27922E-12	6.6869E-11
ENSG00000173762	CD7	0.193647243	0.41220781	0.53891103
ENSG00000125726	CD70	-0.890396439	2.57589E-17	3.16042E-16
ENSG00000019582	CD74	-0.980991599	3.97592E-08	2.07921E-07
ENSG00000110651	CD81	-0.367852722	7.79728E-06	3.27281E-05
ENSG00000114013	CD86	-0.485908409	0.009089208	0.021965586

ENSG00000010278	CD9	-0.235705576	0.503677482	0.617973526
ENSG00000237350	CDC42P6	-1.122581862	0.00087433	0.002606647
ENSG00000123374	CDK2	-0.408443317	0.00625377	0.015464749
ENSG00000124762	CDKN1A	1.233920228	1.23223E-14	1.19116E-13
ENSG00000111276	CDKN1B	0.2446965	2.11787E-05	8.44501E-05
ENSG00000172216	CEBPB	0.953768637	3.5036E-06	1.55462E-05
ENSG00000213341	CHUK	-0.202205354	0.230867874	0.339386414
ENSG00000179583	CIITA	-0.654882861	0.253847956	0.363127793
ENSG00000174600	CMKLR1	0.490248518	0.434848008	0.561605323
ENSG00000109846	CRYAB	0.875356772	8.42087E-16	9.59378E-15
ENSG00000184371	CSF1	0.626680066	8.88282E-11	5.66724E-10
ENSG00000182578	CSF1R	-0.498579968	0.157835731	0.250495513
ENSG00000164400	CSF2	-2.004967867	1.19424E-18	1.73165E-17
ENSG00000198223	CSF2RA	-0.238029018	0.747649101	0.81399339
ENSG00000006210	CX3CL1	1.592617956	2.9867E-25	6.80541E-24
ENSG00000163739	CXCL1	-0.256726123	0.44682701	0.566816586
ENSG00000169245	CXCL10	0.304944154	0.403369659	0.531714551
ENSG00000169248	CXCL11	-0.545139827	0.043280995	0.085774646
ENSG00000107562	CXCL12	-3.327989239	2.09556E-92	3.34242E-90
ENSG00000145824	CXCL14	-2.188931041	7.66394E-15	7.63999E-14
ENSG00000161921	CXCL16	1.027162879	1.91772E-14	1.79927E-13
ENSG00000081041	CXCL2	0.16898917	0.50099077	0.617050408
ENSG00000163734	CXCL3	1.275341357	0.001065824	0.003063044
ENSG00000163735	CXCL5	0.211438549	0.738045595	0.809060291
ENSG00000124875	CXCL6	-0.652575865	0.03078265	0.064180819
ENSG00000169429	CXCL8	-1.229520866	4.53237E-14	4.13093E-13
ENSG00000121966	CXCR4	-0.546204436	0.004927288	0.012675846
ENSG00000107201	DDX58	0.489469915	7.51315E-06	3.19559E-05
ENSG00000149091	DGKZ	-0.346542613	0.156011625	0.248838541
ENSG00000179611	DGKZP1	0.255069726	0.736467145	0.809060291
ENSG00000197635	DPP4	-0.422635195	0.105098109	0.179861411
ENSG00000105246	EBI3	0.222604479	0.475315488	0.594610356
ENSG00000138798	EGF	-0.493059245	0.166079093	0.260981432
ENSG00000146648	EGFR	0.268056975	0.072348886	0.131881683
ENSG00000120738	EGR1	0.057122636	0.616235717	0.712243455
ENSG00000122877	EGR2	-0.21438482	0.353334185	0.477600021
ENSG00000179388	EGR3	-0.300393977	0.081145524	0.143013382
ENSG00000055332	EIF2AK2	0.934555824	8.74139E-27	2.32375E-25
ENSG00000126767	ELK1	-0.02147249	0.882583669	0.926132205
ENSG00000119888	EPCAM	1.279858343	3.7221E-12	2.89597E-11
ENSG00000141736	ERBB2	0.322050831	0.064403777	0.118756097
ENSG00000117525	F3	-1.906889302	4.70687E-80	3.75373E-78
ENSG00000168040	FADD	-0.264435748	0.659617393	0.753939203
ENSG00000026103	FAS	-0.635443279	3.50887E-06	1.55462E-05
ENSG00000112787	FBRSL1	0.69949484	6.85853E-15	7.05765E-14
ENSG00000137312	FLOT1	-0.142447623	0.573351039	0.684560937
ENSG00000170345	FOS	0.424561786	0.002373003	0.006308234
ENSG00000175592	FOSL1	-0.989466545	2.62282E-39	1.67336E-37
ENSG00000114861	FOXP1	0.449135428	0.002148281	0.005758836
ENSG00000049768	FOXP3	-0.032791642	0.276153766	0.39327255

ENSG00000107485	GATA3	-0.730162421	0.000614251	0.001866154
ENSG00000117228	GBP1	-0.282126749	0.177580496	0.27234701
ENSG00000162676	GFI1	-0.716516919	0.055185004	0.104165777
ENSG00000105220	GPI	-0.048455784	0.987738186	0.994937056
ENSG00000145649	GZMA	-0.088025946	0.856646317	0.907874335
ENSG00000135077	HAVCR2	-0.157079263	0.721436033	0.807502086
ENSG00000094631	HDAC6	0.139491869	0.720058437	0.807502086
ENSG00000048052	HDAC9	-1.627785787	4.02E-12	3.05329E-11
ENSG00000100644	HIF1A	0.034646157	0.601050404	0.702326297
ENSG00000203812	HIST2H2AA3	0.011335204	0.988822153	0.994937056
ENSG00000272196	HIST2H2AA4	-0.744939613	2.21682E-05	8.73043E-05
ENSG00000184260	HIST2H2AC	-0.774666273	0.137647674	0.225177476
ENSG00000206503	HLA-A	0.244950152	0.015583737	0.035508657
ENSG00000234745	HLA-B	0.065244003	0.987487336	0.994937056
ENSG00000204525	HLA-C	0.303174007	0.302410027	0.423027522
ENSG00000204632	HLA-G	-0.17412716	0.704349651	0.796764322
ENSG00000189403	HMGB1	-0.225266207	0.465147057	0.584180753
ENSG00000080824	HSP90AA1	-0.121935656	0.378198625	0.50060316
ENSG00000106211	HSPB1	-0.372882631	0.144638684	0.232175554
ENSG00000152137	HSPB8	-0.913265627	0.02290728	0.050050838
ENSG00000144381	HSPD1	0.421950219	0.006245032	0.015464749
ENSG00000090339	ICAM1	0.384945305	0.000598464	0.001835674
ENSG00000115738	ID2	-0.916177541	2.17081E-09	1.33171E-08
ENSG00000131203	IDO1	-0.627473031	0.028605988	0.060034936
ENSG00000163565	IFI16	0.88043295	3.97028E-11	2.69472E-10
ENSG00000165949	IFI27	0.58933303	0.317268444	0.438132613
ENSG00000137965	IFI44	0.579497784	2.01053E-06	9.29507E-06
ENSG00000137959	IFI44L	0.644360284	2.34603E-05	9.12661E-05
ENSG00000126709	IFI6	-0.036481871	0.010350173	0.024824852
ENSG00000115267	IFIH1	0.6928157	9.62112E-06	3.98589E-05
ENSG00000185745	IFIT1	0.892114874	4.04965E-18	5.38266E-17
ENSG00000119922	IFIT2	0.433610889	0.001050791	0.003047293
ENSG00000119917	IFIT3	-0.264360187	1.19455E-06	5.60384E-06
ENSG00000185885	IFITM1	-0.28528331	0.594467197	0.702326297
ENSG00000185201	IFITM2	1.160122873	1.55151E-22	2.6049E-21
ENSG00000142089	IFITM3	0.589968734	6.51518E-05	0.000236175
ENSG00000142166	IFNAR1	-0.303655489	0.00028719	0.000934834
ENSG00000171855	IFNB1	-0.111656213	0.037165742	0.075037163
ENSG00000184995	IFNE	0.049756464	0.484289324	0.601121768
ENSG00000027697	IFNGR1	-0.877736497	2.79737E-25	6.80541E-24
ENSG00000159128	IFNGR2	-0.010462223	0.36514822	0.489934591
ENSG00000185436	IFNLR1	0.109911094	0.776198126	0.836510819
ENSG00000104365	IKBKB	-0.481688434	0.047804811	0.092422635
ENSG00000095752	IL11	-0.023695407	0.968981908	0.987556641
ENSG00000137070	IL11RA	-0.127641713	0.793533159	0.84945328
ENSG00000168811	IL12A	0.290035659	0.417982594	0.54201808
ENSG00000113302	IL12B	-0.579879283	0.251723765	0.361711176
ENSG00000081985	IL12RB2	-0.39463616	0.600453624	0.702326297
ENSG00000131724	IL13RA1	-0.023948751	0.930436043	0.963665902
ENSG00000164136	IL15	0.079818939	0.897205256	0.935321819

ENSG00000172349	IL16	0.406929818	0.110752503	0.186931473
ENSG00000177663	IL17RA	-0.014236568	0.73513381	0.809060291
ENSG00000056736	IL17RB	0.032292704	0.986367273	0.994937056
ENSG00000163701	IL17RE	0.242128033	0.747017489	0.81399339
ENSG00000150782	IL18	0.08197776	0.757516753	0.819145235
ENSG00000115604	IL18R1	0.286428806	0.235828357	0.344978322
ENSG00000115008	IL1A	0.732997098	0.062150541	0.115941652
ENSG00000125538	IL1B	-1.298676117	9.20944E-09	5.24609E-08
ENSG00000115594	IL1R1	0.555958474	1.89985E-05	7.67153E-05
ENSG00000196083	IL1RAP	-0.311173894	0.018353827	0.040943154
ENSG00000174564	IL20RB	1.824357719	4.93701E-11	3.28106E-10
ENSG00000103522	IL21R	-1.189629771	0.01179718	0.028084331
ENSG00000110944	IL23A	0.308365442	0.07972824	0.142085522
ENSG00000104998	IL27RA	1.229307085	2.40067E-19	3.64673E-18
ENSG00000100385	IL2RB	0.567453771	0.038877781	0.078000077
ENSG00000164509	IL31RA	-1.61569521	1.37021E-05	5.60382E-05
ENSG00000113520	IL4	-0.531208602	0.236838239	0.344978322
ENSG00000077238	IL4R	0.01998452	0.440852141	0.564786477
ENSG00000136244	IL6	-1.971895691	9.01516E-88	9.58612E-86
ENSG00000160712	IL6R	0.590925517	0.18870542	0.285293976
ENSG00000104432	IL7	-0.202328947	0.304869346	0.423027522
ENSG00000168685	IL7R	-0.666594212	2.35922E-08	1.25432E-07
ENSG00000123999	INHA	1.334998712	2.07033E-08	1.11938E-07
ENSG00000122641	INHBA	-0.921827503	0.014328474	0.033121617
ENSG00000184216	IRAK1	-0.155125663	0.905628859	0.941028033
ENSG00000134070	IRAK2	0.038346324	0.439729446	0.564786477
ENSG00000198001	IRAK4	-0.442872703	3.15542E-05	0.000118421
ENSG00000125347	IRF1	-0.178422903	0.542619584	0.660670409
ENSG00000168310	IRF2	-0.111740425	0.410296433	0.538619598
ENSG00000170604	IRF2BP1	0.372723335	0.043290652	0.085774646
ENSG00000126456	IRF3	0.32199283	0.072992593	0.132299075
ENSG00000128604	IRF5	0.363385228	0.050229856	0.095948048
ENSG00000117595	IRF6	0.005541357	0.991818131	0.994937056
ENSG00000185507	IRF7	0.277472555	0.575117025	0.684560937
ENSG00000187608	ISG15	-0.068314877	2.69102E-09	1.61969E-08
ENSG00000172183	ISG20	0.315827996	0.076345757	0.137594895
ENSG00000078747	ITCH	0.063536834	0.447767335	0.566816586
ENSG00000213949	ITGA1	-0.389326669	0.001785661	0.004827339
ENSG00000005961	ITGA2B	0.806049523	0.032128985	0.066517141
ENSG00000162434	JAK1	0.40872524	6.37598E-05	0.000236175
ENSG00000096968	JAK2	0.056258137	0.864490512	0.913153885
ENSG00000105639	JAK3	0.984914992	0.000427371	0.001377084
ENSG00000177606	JUN	0.199619776	0.113727882	0.190943128
ENSG00000100578	KIAA0586	0.011600298	0.867393287	0.913196233
ENSG00000049130	KITLG	-0.740416698	7.93739E-11	5.1674E-10
ENSG00000089692	LAG3	0.252312739	0.174466015	0.27148614
ENSG00000116678	LEPR	0.487216877	7.87177E-05	0.00027901
ENSG00000131981	LGALS3	-0.145042979	0.724227013	0.807791668
ENSG00000128342	LIF	-1.396269716	9.43293E-12	6.6869E-11
ENSG00000123384	LRP1	0.745868964	2.62721E-07	1.28935E-06

ENSG00000154589	LY96	-0.236886895	0.297594507	0.418205496
ENSG00000254087	LYN	-0.258363959	0.016596316	0.037283273
ENSG00000090382	LYZ	-0.730772412	0.044560549	0.087745772
ENSG00000178573	MAF	-1.359223515	1.80189E-31	5.22547E-30
ENSG00000034152	MAP2K3	-0.878983872	5.21609E-06	2.24856E-05
ENSG00000095015	MAP3K1	-0.07737949	0.598182143	0.702326297
ENSG00000169967	MAP3K2	0.300118814	0.019025327	0.041855719
ENSG00000135341	MAP3K7	0.230581024	0.012545429	0.02942641
ENSG00000100030	MAPK1	-0.220988844	0.144836788	0.232175554
ENSG00000107643	MAPK8	-0.360912241	0.004298371	0.011239184
ENSG00000105976	MET	-0.106505536	0.417430441	0.54201808
ENSG00000130731	METTL26	-0.075718576	0.891957539	0.932899853
ENSG00000204520	MICA	-0.374428145	0.612842315	0.710897086
ENSG00000158411	MITD1	-0.00898693	0.995587973	0.995587973
ENSG00000156738	MS4A1	-0.546029509	6.61054E-12	4.9041E-11
ENSG00000196814	MVB12B	0.064484205	0.784360755	0.842461552
ENSG00000157601	MX1	1.011071208	8.83519E-16	9.71871E-15
ENSG00000183486	MX2	0.771396945	1.66469E-13	1.4751E-12
ENSG00000136997	MYC	-1.884866086	1.33905E-18	1.85721E-17
ENSG00000172936	MYD88	0.458234901	0.00059755	0.001835674
ENSG00000158092	NCK1	0.176751932	0.138927947	0.226112322
ENSG00000184983	NDUFA6	-0.460637166	0.035515865	0.072162809
ENSG00000131196	NFATC1	0.709383845	0.045270649	0.088056933
ENSG00000101096	NFATC2	0.301775669	0.208798156	0.309798194
ENSG00000072736	NFATC3	0.177143183	0.144148722	0.232175554
ENSG00000109320	NFKB1	0.169946669	0.118052311	0.197165902
ENSG00000077150	NFKB2	0.585967632	0.001325529	0.003645204
ENSG00000100906	NFKBIA	0.81635959	1.05153E-07	5.32443E-07
ENSG00000170322	NFRKB	-0.041432163	0.555357993	0.673609124
ENSG00000123609	NMI	-8.55715E-05	0.480196553	0.598369924
ENSG00000106100	NOD1	-0.034597205	0.963739969	0.98536234
ENSG00000148400	NOTCH1	0.19354919	0.032320241	0.066517141
ENSG00000177463	NR2C2	0.15987576	0.292674749	0.413111704
ENSG00000113580	NR3C1	0.136643203	0.207888014	0.309798194
ENSG00000123358	NR4A1	-1.834040882	1.1917E-24	2.37595E-23
ENSG00000119508	NR4A3	-0.780222073	0.001259992	0.003523432
ENSG00000089127	OAS1	0.553233753	0.000204868	0.000687927
ENSG00000111335	OAS2	0.750176207	2.45172E-05	9.42287E-05
ENSG00000261371	PECAM1	-0.545479502	0.305004169	0.423027522
ENSG00000197329	PELI1	-0.032121418	0.73152344	0.809060291
ENSG00000140464	PML	-0.041540895	0.9484604	0.975996347
ENSG00000028277	POU2F2	-0.833602926	0.016356095	0.037004216
ENSG00000186951	PPARA	0.95882702	5.84911E-13	4.91017E-12
ENSG00000132170	PPARG	0.687189428	1.39627E-07	6.95952E-07
ENSG00000084072	PPIE	-0.503148344	5.97684E-09	3.53076E-08
ENSG00000100023	PPIL2	-0.032968872	0.960230667	0.984931135
ENSG00000067606	PRKCZ	0.369434165	0.347858073	0.474216775
ENSG00000180228	PRKRA	-0.07631568	0.70743495	0.797426675
ENSG00000092010	PSME1	-0.073083801	0.948364143	0.975996347
ENSG00000125384	PTGER2	0.801107023	0.00062447	0.001879302

ENSG00000073756	PTGS2	-0.264995628	0.086974988	0.151612138
ENSG00000111737	RAB35	-0.385137827	0.000277012	0.000910997
ENSG00000136238	RAC1	-0.480117782	5.13073E-06	2.24206E-05
ENSG00000162924	REL	0.634721177	8.52284E-09	4.94325E-08
ENSG00000173039	RELA	0.630070775	7.61514E-05	0.000272947
ENSG00000104856	RELB	1.054694542	2.58112E-10	1.61446E-09
ENSG00000132005	RFX1	0.695750185	1.94034E-12	1.54742E-11
ENSG00000104312	RIPK2	0.138771158	0.206028771	0.308559522
ENSG00000133135	RNF128	0.151519781	0.666111409	0.756190532
ENSG00000069667	RORA	0.854830171	0.000228605	0.000759634
ENSG00000143365	RORC	2.148110527	5.56828E-24	1.04487E-22
ENSG00000159216	RUNX1	-0.174847556	0.045045363	0.088056933
ENSG00000020633	RUNX3	0.626277878	0.07702144	0.138032805
ENSG00000170989	S1PR1	-0.41133764	0.341468041	0.467503455
ENSG00000188404	SELL	-0.336566436	0.085834321	0.15044587
ENSG00000133661	SFTPD	-0.662518086	0.211477079	0.312320316
ENSG00000185187	SIGIRR	-0.166038622	0.606459931	0.706061015
ENSG00000145147	SLIT2	-0.35604181	0.377053222	0.50060316
ENSG00000185338	SOCS1	-1.518815899	5.75265E-15	6.11698E-14
ENSG00000184557	SOCS3	-0.208167805	0.160839205	0.253998547
ENSG00000171150	SOCS5	0.041002561	0.237916084	0.344978322
ENSG00000118785	SPP1	-1.522673049	1.79835E-08	9.89091E-08
ENSG00000115415	STAT1	0.434467072	0.001270203	0.003523432
ENSG00000170581	STAT2	0.469603186	0.000138665	0.000470576
ENSG00000168610	STAT3	0.005097315	0.808917429	0.863025618
ENSG00000138378	STAT4	0.61959262	0.175695205	0.272071701
ENSG00000126561	STAT5A	0.767086505	2.9399E-05	0.000111646
ENSG00000166888	STAT6	0.290701262	0.514357622	0.628659316
ENSG00000231925	TAPBP	-0.949707502	0.007581273	0.018461268
ENSG00000183735	TBK1	-0.282468587	0.00548934	0.013788184
ENSG00000073861	TBX21	-0.687659487	0.005423844	0.013731796
ENSG00000163235	TGFA	-0.112589935	0.3658403	0.489934591
ENSG00000105329	TGFB1	-0.84876235	8.23122E-05	0.000288545
ENSG00000092969	TGFB2	-0.136965573	0.110499925	0.186931473
ENSG00000119699	TGFB3	-0.713660284	0.001015027	0.002998088
ENSG00000041988	THAP3	-0.308043773	0.18813454	0.285293976
ENSG00000137801	THBS1	-1.785709452	1.84306E-20	2.93968E-19
ENSG00000127666	TICAM1	0.575931221	0.005149834	0.013142376
ENSG00000102265	TIMP1	-0.060193641	0.488817606	0.604390761
ENSG00000150455	TIRAP	0.268160861	0.558646399	0.675031065
ENSG00000174125	TLR1	0.030329756	0.813628556	0.865158365
ENSG00000137462	TLR2	-0.473831319	0.089652165	0.155429569
ENSG00000164342	TLR3	-0.180785991	0.367066982	0.489934591
ENSG00000136869	TLR4	-1.530189918	8.09904E-24	1.43533E-22
ENSG00000187554	TLR5	1.083185801	0.000134366	0.000460891
ENSG00000174130	TLR6	-0.942341936	0.000527149	0.001664955
ENSG00000184584	TMEM173	-0.437344029	6.49704E-05	0.000236175
ENSG00000104689	TNFRSF10A	-0.075177861	0.637972295	0.732061734
ENSG00000164761	TNFRSF11B	-1.555052853	3.4543E-34	1.57417E-32
ENSG00000157873	TNFRSF14	-0.05349118	0.729741278	0.809060291

ENSG00000186891	TNFRSF18	-0.973308058	0.027655069	0.058423622
ENSG00000067182	TNFRSF1A	-0.453476802	0.001216854	0.003435188
ENSG00000120949	TNFRSF8	0.843273547	0.025069391	0.054034701
ENSG00000049249	TNFRSF9	1.026808566	9.86572E-12	6.84166E-11
ENSG00000121858	TNFSF10	-0.835848534	8.67354E-08	4.46268E-07
ENSG00000239697	TNFSF12	0.540441969	0.001044565	0.003047293
ENSG00000102524	TNFSF13B	-0.483172904	0.451229309	0.568941302
ENSG00000125735	TNFSF14	0.419078733	0.580642831	0.688569007
ENSG00000117586	TNFSF4	0.534662429	0.099454422	0.171491679
ENSG00000078902	TOLLIP	-0.161550309	0.134002491	0.220344302
ENSG00000141510	TP53	-0.374956324	0.001167194	0.003324419
ENSG00000164938	TP53INP1	-0.268556744	0.049985555	0.095948048
ENSG00000131323	TRAF3	0.364981998	0.001419916	0.003871394
ENSG00000175104	TRAF6	0.193408569	0.062892047	0.116642808
ENSG00000074319	TSG101	-0.871119333	2.27758E-36	1.21091E-34
ENSG00000084652	TXLNA	-0.252964053	0.126529837	0.210224053
ENSG00000105397	TYK2	-0.531535121	0.054992669	0.104165777
ENSG00000025708	TYMP	0.67227204	0.179303092	0.27367314
ENSG00000177889	UBE2N	-1.202750711	4.85163E-25	1.03178E-23
ENSG00000162692	VCAM1	0.190142627	0.24378866	0.351894039
ENSG00000112715	VEGFA	1.299216114	7.91541E-13	6.4744E-12
ENSG00000167987	VPS37C	-0.2281195	0.131813509	0.217867923
ENSG00000160685	ZBTB7B	0.137561148	0.352794474	0.477600021
ENSG00000083838	ZNF446	0.400549486	0.025905571	0.055092515
