# Supplementary material for: Microglial derived extracellular vesicles activate autophagy and mediate multi‐target signaling to maintain cellular homeostasis
Source: J Extracell Vesicles. 2020 Nov 25;10(1):e12022. doi: 10.1002/jev2.12022 (PMC7890546; doi:10.1002/jev2.12022)
Supplement: Supplementary file 5 — Supplementary table 3: differential expressed gene counts tables from nEVs + TNFα‐stimulated cells compared to TNFα only‐stimulated cells. [file JEV2-10-e12022-s005.pdf]

Supplementary table 3 (TNFα-stimulated C20 cells vs TNFα and nEVs stimulated C20 cells)

ensembl	hgnc_symbol	log2FoldChange	pvalue	padj
ENSG00000144476	ACKR3	-0.341734089	0.18937615	0.33354638
ENSG00000129048	ACKR4	-0.61975429	0.167677234	0.305172566
ENSG00000196839	ADA	0.331416004	0.402123541	0.577788036
ENSG00000160710	ADAR	0.320137083	1.03783E-05	5.66653E-05
ENSG00000164022	AIMP1	-0.330488719	0.002755866	0.009064474
ENSG00000164111	ANXA5	0.157635322	0.012939941	0.034976275
ENSG00000115307	AUP1	0.089383957	0.313796023	0.499031547
ENSG00000171791	BCL2	-0.487487544	0.038264152	0.091632575
ENSG00000113916	BCL6	0.111645536	0.360021202	0.545711699
ENSG00000125845	BMP2	-0.27903211	0.504432482	NA
ENSG00000125378	BMP4	0.398917089	0.375222254	0.559757788
ENSG00000153162	BMP6	-0.795036936	0.072504947	0.157094052
ENSG00000130303	BST2	0.050482189	0.455924836	0.63499423
ENSG00000125730	C3	0.493434078	1.31447E-07	9.69868E-07
ENSG00000171860	C3AR1	-0.168959359	0.534774474	0.695206816
ENSG00000106804	C5	-0.704136567	0.042385637	0.099164384
ENSG00000137752	CASP1	-0.053584231	0.671114218	0.783766701
ENSG00000064012	CASP8	-0.264138819	0.089482235	0.179622429
ENSG00000105974	CAV1	0.444050968	1.10935E-06	7.21078E-06
ENSG00000114423	CBLB	0.173309471	0.257793531	0.429131914
ENSG00000108691	CCL2	-0.066202021	0.314408154	0.499031547
ENSG00000115009	CCL20	0.794177185	0.011853103	0.032685829
ENSG00000006606	CCL26	-0.408310262	0.361808855	0.545711699
ENSG00000108688	CCL7	-1.180817597	0.006677554	0.019601852
ENSG00000163823	CCR1	0.343574744	0.444777938	0.625898852
ENSG00000184451	CCR10	0.178901193	0.679150907	0.785627955
ENSG00000121797	CCRL2	0.257088455	0.569793685	0.70858032
ENSG00000170458	CD14	0.441209914	0.006583151	0.019534785
ENSG00000120217	CD274	-0.315206141	0.020408756	0.051588801
ENSG00000103855	CD276	0.413156143	0.00231007	0.007883113
ENSG00000101017	CD40	-0.202050073	0.643721408	NA
ENSG00000026508	CD44	0.025248389	0.776222346	0.857929961
ENSG00000196776	CD47	-0.062839078	0.486330614	0.657268602
ENSG00000135404	CD63	-0.075063233	0.28074131	0.458936393
ENSG00000019582	CD74	-2.250872018	3.28898E-32	8.97893E-31
ENSG00000110651	CD81	0.479872881	3.5602E-11	4.2258E-10
ENSG00000010278	CD9	0.017130862	0.812489608	0.883703836
ENSG00000237350	CDC42P6	-0.311256406	0.356272913	0.543365952
ENSG00000123374	CDK2	-0.015934061	0.893584417	0.934668758
ENSG00000124762	CDKN1A	-1.289123962	5.21177E-35	1.5809E-33
ENSG00000111276	CDKN1B	0.013557323	0.917002479	0.948263927
ENSG00000172216	CEBPB	-0.101057995	0.551592667	0.701417115
ENSG00000213341	CHUK	-0.197273271	0.05696867	0.129603723
ENSG00000179583	CIITA	-0.392707896	0.384804007	0.564255079
ENSG00000109846	CRYAB	0.36320347	0.188672214	0.33354638

ENSG00000184371	CSF1	1.132085319	5.05378E-39	1.7246E-37
ENSG00000182578	CSF1R	0.477060081	0.291616297	0.468301466
ENSG00000164400	CSF2	-1.774925247	6.00709E-54	2.34276E-52
ENSG00000108342	CSF3	-2.176578376	7.40548E-07	5.05424E-06
ENSG00000006210	CX3CL1	0.508211819	0.006942322	0.019950042
ENSG00000163739	CXCL1	-3.12917192	2.3629E-154	1.6127E-152
ENSG00000169245	CXCL10	0.762722403	2.13745E-25	4.86269E-24
ENSG00000169248	CXCL11	-0.127622267	0.110395386	0.216819715
ENSG00000107562	CXCL12	0.091591921	0.628459859	0.749211972
ENSG00000161921	CXCL16	0.279496646	0.13361524	0.251565246
ENSG00000081041	CXCL2	-2.904067235	3.1143E-107	1.7004E-105
ENSG00000163734	CXCL3	-1.985622079	7.28685E-08	5.85091E-07
ENSG00000163735	CXCL5	0.404724996	0.004286772	0.013298735
ENSG00000124875	CXCL6	-3.062769275	7.617E-190	6.9315E-188
ENSG00000169429	CXCL8	-3.205585549	0	0
ENSG00000138755	CXCL9	-0.276262478	0.50445284	NA
ENSG00000121966	CXCR4	0.756798006	0.011425415	0.032156064
ENSG00000107201	DDX58	0.220142516	0.003782608	0.012148848
ENSG00000149091	DGKZ	0.031107277	0.810380406	0.883703836
ENSG00000197635	DPP4	0.170854414	0.602048424	0.727253185
ENSG00000105246	EBI3	0.525695656	0.004865875	0.014759819
ENSG00000138798	EGF	-0.508351957	0.240174767	0.402255898
ENSG00000146648	EGFR	0.369404593	0.002652374	0.008830466
ENSG00000120738	EGR1	-1.328291877	5.34397E-13	6.94716E-12
ENSG00000122877	EGR2	-0.242624806	0.581615863	0.712023007
ENSG00000179388	EGR3	-0.539322326	0.231290677	0.394639717
ENSG00000055332	EIF2AK2	0.114896677	0.132022826	0.250293274
ENSG00000126767	ELK1	0.287793974	0.022630575	0.056164973
ENSG00000119888	EPCAM	-0.804344776	0.037274727	NA
ENSG00000141736	ERBB2	0.605125816	0.003832878	0.012167159
ENSG00000117525	F3	0.083444478	0.342204797	0.527855361
ENSG00000026103	FAS	-1.411775502	4.84281E-23	9.44348E-22
ENSG00000112787	FBRSL1	-0.131232414	0.287981459	0.467969871
ENSG00000170345	FOS	-0.875738659	0.000150543	0.000684969
ENSG00000175592	FOSL1	-0.230687611	0.00044984	0.0018607
ENSG00000114861	FOXP1	-0.205964364	0.041069859	0.097496275
ENSG00000107485	GATA3	0.256612658	0.389352075	0.564255079
ENSG00000117228	GBP1	0.462285205	5.94327E-10	6.0093E-09
ENSG00000162676	GFI1	-0.692599849	0.104872997	0.207466145
ENSG00000105220	GPI	0.225305746	0.003579425	0.011633132
ENSG00000094631	HDAC6	0.558275494	0.011785655	0.032685829
ENSG00000048052	HDAC9	-0.372916956	0.006907231	0.019950042
ENSG00000100644	HIF1A	0.264697294	0.00067482	0.002669939
ENSG00000203812	HIST2H2AA3	-0.39774054	0.013522988	0.035842483
ENSG00000272196	HIST2H2AA4	-0.488875099	0.00394412	0.012376377
ENSG00000184260	HIST2H2AC	0.013087491	0.976446646	0.980036523
ENSG00000206503	HLA-A	1.009506151	NA	NA
ENSG00000234745	HLA-B	-0.087760679	0.819295284	0.88406171
ENSG00000204525	HLA-C	0.084743837	0.742806703	0.844942625
ENSG00000204632	HLA-G	0.120899856	0.757079908	0.847634623

ENSG00000189403	HMGB1	-0.40875108	1.21097E-05	6.48223E-05
ENSG00000117594	HSD11B1	0.334504454	0.067132311	0.147799362
ENSG00000080824	HSP90AA1	0.07695112	0.346310847	0.53113967
ENSG00000106211	HSPB1	0.471285627	0.000530726	0.00216251
ENSG00000144381	HSPD1	-0.066219676	0.389078174	0.564255079
ENSG00000090339	ICAM1	0.221846713	0.001390896	0.005062861
ENSG00000115738	ID2	-1.295869534	7.38371E-06	4.11378E-05
ENSG00000131203	IDO1	0.166071291	0.321579681	0.50746389
ENSG00000163565	IFI16	0.40861081	1.96764E-06	1.19517E-05
ENSG00000165949	IFI27	0.00190317	0.991670047	0.991670047
ENSG00000137965	IFI44	-0.416477155	1.95325E-10	2.05092E-09
ENSG00000137959	IFI44L	-0.572775414	5.93189E-17	9.52592E-16
ENSG00000126709	IFI6	0.311725659	0.000324815	0.00139572
ENSG00000115267	IFIH1	0.495323406	9.9877E-07	6.65035E-06
ENSG00000185745	IFIT1	0.188482403	0.015776648	0.041019286
ENSG00000119922	IFIT2	0.670749906	6.12554E-20	1.11485E-18
ENSG00000119917	IFIT3	0.305282544	1.97006E-06	1.19517E-05
ENSG00000185885	IFITM1	-0.038706149	0.921774822	0.949601986
ENSG00000185201	IFITM2	0.273700294	0.015438045	0.040524869
ENSG00000142089	IFITM3	0.279180931	0.000831088	0.003195592
ENSG00000142166	IFNAR1	-0.154481047	0.07768507	0.165687689
ENSG00000171855	IFNB1	-0.288259116	0.516244042	0.677664636
ENSG00000184995	IFNE	-0.248267302	0.566977085	0.70858032
ENSG00000027697	IFNGR1	-0.318876375	0.000387571	0.001627797
ENSG00000159128	IFNGR2	-0.213288978	0.581381986	0.712023007
ENSG00000185436	IFNLR1	-0.240351204	0.59145013	0.71843307
ENSG00000104365	IKBKB	-0.096882462	0.57361264	0.70858032
ENSG00000095752	IL11	-1.756618784	2.22494E-14	3.37449E-13
ENSG00000137070	IL11RA	0.09400567	0.834344555	0.893239465
ENSG00000168811	IL12A	-0.775820669	0.004544063	0.013938531
ENSG00000096996	IL12RB1	-0.083616549	0.836505988	NA
ENSG00000131724	IL13RA1	-0.18300129	0.063709576	0.142563232
ENSG00000164136	IL15	-0.432216112	0.026617475	0.064880096
ENSG00000177663	IL17RA	0.526231284	0.209126563	0.359066363
ENSG00000163701	IL17RE	0.02840527	0.947267526	NA
ENSG00000115604	IL18R1	0.096286918	0.763216149	0.847634623
ENSG00000115008	IL1A	-2.195702993	2.94287E-07	2.11422E-06
ENSG00000125538	IL1B	-1.909398962	3.877E-13	5.2921E-12
ENSG00000115594	IL1R1	0.38357722	0.018385497	0.047351327
ENSG00000196083	IL1RAP	-0.207309147	0.195008058	0.339090445
ENSG00000136689	IL1RN	-0.594889155	0.160710544	0.294456231
ENSG00000174564	IL20RB	-0.140735695	0.720161514	0.82261127
ENSG00000103522	IL21R	0.187537711	0.667753015	NA
ENSG00000110944	IL23A	-0.537946733	0.235056518	0.396372954
ENSG00000104998	IL27RA	-0.114644294	0.637955191	0.757225075
ENSG00000164509	IL31RA	-0.351504882	0.437658797	0.622036126
ENSG00000077238	IL4R	-0.132320053	0.512079024	0.677664636
ENSG00000136244	IL6	-3.307326566	1.4755E-269	2.014E-267
ENSG00000160712	IL6R	0.180091204	0.669935145	NA
ENSG00000104432	IL7	-0.344118147	0.27725256	0.455963548

ENSG00000168685	IL7R	1.679264227	1.88218E-60	8.56393E-59
ENSG00000123999	INHA	-1.361931909	0.001710945	0.006066079
ENSG00000122641	INHBA	1.179441788	1.90964E-08	1.73777E-07
ENSG00000184216	IRAK1	0.324596671	0.338116212	0.527461291
ENSG00000134070	IRAK2	-0.183397127	0.273708905	0.452863824
ENSG00000198001	IRAK4	-0.541436229	0.000237805	0.001064277
ENSG00000125347	IRF1	0.108868079	0.511602952	0.677664636
ENSG00000168310	IRF2	-0.019660614	0.806733817	0.883703836
ENSG00000170604	IRF2BP1	0.264115009	0.140427636	0.262580443
ENSG00000126456	IRF3	0.123055072	0.186050084	0.331971718
ENSG00000128604	IRF5	-0.26566951	0.548903466	0.701417115
ENSG00000117595	IRF6	0.102963051	0.815405462	NA
ENSG00000185507	IRF7	0.153531492	0.687903723	0.792395427
ENSG00000187608	ISG15	0.507439308	3.55875E-11	4.2258E-10
ENSG00000172183	ISG20	0.517218666	2.59794E-09	2.44565E-08
ENSG00000078747	ITCH	-0.024047422	0.847281219	0.903545987
ENSG00000213949	ITGA1	-0.231828792	0.176413361	0.318946009
ENSG00000160255	ITGB2	0.075748886	0.865318476	0.912092447
ENSG00000162434	JAK1	0.187872366	0.084812914	0.175554753
ENSG00000096968	JAK2	0.022717208	0.906109575	0.940991222
ENSG00000177606	JUN	-0.090723842	0.369665343	0.554498015
ENSG00000100578	KIAA0586	0.073186455	0.659693316	0.779637555
ENSG00000049130	KITLG	-0.115265286	0.543534613	0.701417115
ENSG00000116678	LEPR	-1.299931638	2.06904E-05	0.000106575
ENSG00000131981	LGALS3	-0.887201378	1.31328E-26	3.25933E-25
ENSG00000128342	LIF	-0.439987691	0.184972306	0.331971718
ENSG00000123384	LRP1	-0.119866634	0.458423131	0.63499423
ENSG00000154589	LY96	-2.70652041	2.40355E-25	5.04746E-24
ENSG00000254087	LYN	0.070039408	0.57198503	0.70858032
ENSG00000178573	MAF	0.11058524	0.751426751	0.847634623
ENSG00000034152	MAP2K3	-0.052303272	0.818720938	0.88406171
ENSG00000095015	MAP3K1	0.412082412	0.009234616	0.026260939
ENSG00000169967	MAP3K2	-0.044014326	0.750281264	0.847634623
ENSG00000135341	MAP3K7	-0.355462629	8.20623E-05	0.000393035
ENSG00000100030	MAPK1	0.013003964	0.875614822	0.919395563
ENSG00000107643	MAPK8	-0.427427912	8.04248E-05	0.000393035
ENSG00000105976	MET	0.009357879	0.932043688	0.956571154
ENSG00000130731	METTL26	-0.225217827	0.033438467	0.08078497
ENSG00000158411	MITD1	-0.62547971	5.16923E-06	3.00255E-05
ENSG00000100985	MMP9	-0.840469384	0.053785281	0.124435439
ENSG00000196814	MVB12B	-0.144411223	0.506566907	0.677664636
ENSG00000157601	MX1	0.681162427	3.30262E-13	4.74534E-12
ENSG00000183486	MX2	0.398930876	1.85139E-10	2.02172E-09
ENSG00000136997	MYC	0.373395641	0.063085389	0.142333151
ENSG00000172936	MYD88	0.415180669	2.32501E-06	1.37984E-05
ENSG00000158092	NCK1	-0.732017661	5.28834E-06	3.00774E-05
ENSG00000184983	NDUFA6	-0.884023075	0.000916407	0.00347471
ENSG00000131196	NFATC1	0.319515226	0.462141042	0.63499423
ENSG00000072736	NFATC3	0.105318386	0.465197238	0.63499423
ENSG00000109320	NFKB1	0.159981338	0.13042679	0.250293274

ENSG00000077150	NFKB2	0.210302497	0.080012088	0.168891372
ENSG00000100906	NFKBIA	0.432901038	3.85022E-08	3.28472E-07
ENSG00000170322	NFRKB	0.067585421	0.828381968	0.890347549
ENSG00000123609	NMI	0.145677841	0.085745434	0.176003785
ENSG00000106100	NOD1	0.270525559	0.405825763	0.580054625
ENSG00000148400	NOTCH1	-0.153512365	0.460642135	0.63499423
ENSG00000177463	NR2C2	-0.125501204	0.330954261	0.519255823
ENSG00000113580	NR3C1	-0.243912881	0.00582286	0.017468579
ENSG00000123358	NR4A1	-0.111656795	0.793160884	NA
ENSG00000119508	NR4A3	-1.733275714	1.33864E-05	7.02783E-05
ENSG00000111335	OAS2	0.437772629	1.28708E-06	8.17149E-06
ENSG00000197329	PELI1	-0.312550713	0.054506208	0.125043655
ENSG00000140464	PML	0.421000532	0.001456595	0.005232242
ENSG00000028277	POU2F2	-0.969885833	0.00059787	0.002400271
ENSG00000186951	PPARA	-0.134015846	0.519801366	0.67897499
ENSG00000132170	PPARG	-0.438838809	0.084883617	0.175554753
ENSG00000084072	PPIE	-0.182343691	0.042499022	0.099164384
ENSG00000100023	PPIL2	0.172084121	0.152634367	0.281548528
ENSG00000180228	PRKRA	-0.511358278	8.04676E-08	6.27648E-07
ENSG00000092010	PSME1	-0.28736537	0.5084155	0.677664636
ENSG00000125384	PTGER2	1.361200425	2.48907E-05	0.000125837
ENSG00000073756	PTGS2	-0.655052374	0.118332361	0.230748104
ENSG00000111737	RAB35	0.256569018	0.075423519	0.162130872
ENSG00000136238	RAC1	0.063773594	0.342235894	0.527855361
ENSG00000162924	REL	-0.342781992	0.064746033	0.14370461
ENSG00000173039	RELA	0.141427934	0.089010995	0.179622429
ENSG00000104856	RELB	0.070308722	0.552269458	0.701417115
ENSG00000132005	RFX1	0.005567251	0.965864779	0.972992933
ENSG00000104312	RIPK2	-0.13265344	0.203985482	0.352455928
ENSG00000069667	RORA	-0.940846612	0.023909794	0.058805169
ENSG00000159216	RUNX1	-0.371823667	0.001897277	0.006556413
ENSG00000020633	RUNX3	0.206335858	0.592115167	0.71843307
ENSG00000170989	S1PR1	0.078089644	0.861651293	0.911747298
ENSG00000188404	SELL	0.060616389	0.880044378	NA
ENSG00000185187	SIGIRR	-0.026285641	0.943094208	0.964287337
ENSG00000145147	SLIT2	-0.361484141	0.080424463	0.168891372
ENSG00000185338	SOCS1	-0.043260087	0.854915279	0.908139577
ENSG00000184557	SOCS3	-0.615423959	0.000970557	0.003629618
ENSG00000171150	SOCS5	-0.084069621	0.622423286	0.745269987
ENSG00000118785	SPP1	0.382024941	0.390638132	0.564255079
ENSG00000115415	STAT1	0.188410423	0.00186046	0.006511611
ENSG00000170581	STAT2	0.295159629	0.019970176	0.050951946
ENSG00000168610	STAT3	0.204169463	0.021182944	0.053054528
ENSG00000138378	STAT4	-0.486593381	0.235210324	0.396372954
ENSG00000126561	STAT5A	0.592478662	0.012538544	0.034230225
ENSG00000166888	STAT6	0.302301279	0.464155873	0.63499423
ENSG00000168394	TAP1	-0.107410595	0.798417062	NA
ENSG00000231925	TAPBP	0.434897932	0.290430931	0.468301466
ENSG00000183735	TBK1	0.006276326	0.952688015	0.96685438
ENSG00000105329	TGFB1	-0.15673996	0.670256395	0.783766701

ENSG00000092969	TGFB2	0.491824238	0.000276279	0.00121652
ENSG00000119699	TGFB3	0.052118315	0.906522679	0.940991222
ENSG00000041988	THAP3	-0.089476038	0.67467097	0.783766701
ENSG00000137801	THBS1	1.104391525	4.7623E-08	3.93972E-07
ENSG00000127666	TICAM1	0.508441205	0.002503957	0.008439262
ENSG00000102265	TIMP1	-0.023996254	0.759226128	0.847634623
ENSG00000174125	TLR1	-0.520736675	0.191310533	0.334793433
ENSG00000137462	TLR2	0.687621858	0.001023709	0.003776656
ENSG00000164342	TLR3	0.513125498	0.000737749	0.002877222
ENSG00000136869	TLR4	0.119786608	0.619986981	0.745269987
ENSG00000187554	TLR5	-0.235847828	0.553293497	0.701417115
ENSG00000174130	TLR6	-0.349906123	0.439754477	0.622036126
ENSG00000184584	TMEM173	0.284141742	0.068900104	0.150477828
ENSG00000104689	TNFRSF10A	0.093946154	0.554967388	0.701417115
ENSG00000164761	TNFRSF11B	-0.662942301	1.10815E-07	8.40347E-07
ENSG00000157873	TNFRSF14	0.024833295	0.950019128	0.96685438
ENSG00000186891	TNFRSF18	-0.682927782	0.130645227	0.250293274
ENSG00000067182	TNFRSF1A	0.413297062	9.29189E-05	0.000429947
ENSG00000120949	TNFRSF8	-0.022485832	0.958939133	0.969594013
ENSG00000049249	TNFRSF9	0.067977787	0.483159651	0.656231765
ENSG00000121858	TNFSF10	-0.780459438	1.62187E-18	2.76732E-17
ENSG00000239697	TNFSF12	0.042916258	0.692181192	0.793972544
ENSG00000102524	TNFSF13B	0.173232583	0.672942585	0.783766701
ENSG00000117586	TNFSF4	-0.906616449	0.013425002	0.035842483
ENSG00000078902	TOLLIP	0.157642975	0.384622687	0.564255079
ENSG00000141510	TP53	0.427939711	8.19635E-05	0.000393035
ENSG00000164938	TP53INP1	-0.601658896	0.000327202	0.00139572
ENSG00000131323	TRAF3	0.233689521	0.086603178	0.176437819
ENSG00000175104	TRAF6	-0.271036815	0.143697983	0.266867682
ENSG00000074319	TSG101	-0.479500877	2.25207E-08	1.98327E-07
ENSG00000084652	TXLNA	0.127870528	0.558755391	0.70295033
ENSG00000105397	TYK2	0.230382952	0.377364253	0.559893702
ENSG00000025708	TYMP	0.590318861	0.092524233	0.184373106
ENSG00000177889	UBE2N	-0.407655313	8.95226E-05	0.000421374
ENSG00000162692	VCAM1	-0.171611841	0.131899852	0.250293274
ENSG00000112715	VEGFA	-0.94916269	7.59822E-11	8.64298E-10
ENSG00000167987	VPS37C	0.138410254	0.516315913	0.677664636
ENSG00000160685	ZBTB7B	0.100765446	0.763802627	0.847634623
ENSG00000083838	ZNF446	0.069006121	0.795702212	0.875914129
ENSG00000089127	OAS1	0.494016379	1.28444E-09	1.25233E-08
ENSG00000125726	CD70	-0.497410017	4.58873E-07	3.21211E-06
