# Supplementary material for: Microglial derived extracellular vesicles activate autophagy and mediate multi‐target signaling to maintain cellular homeostasis
Source: J Extracell Vesicles. 2020 Nov 25;10(1):e12022. doi: 10.1002/jev2.12022 (PMC7890546; doi:10.1002/jev2.12022)
Supplement: Supplementary file 6 — Supplementary table 4: differential expressed gene counts tables from aEVs + TNFα‐stimulated cells compared to control TNFα only‐stimulated cells. [file JEV2-10-e12022-s006.pdf]

Supplementary table 4 (TNFα-stimulated C20 cells vs TNFα and aEVs stimulated C20 cells)

ensembl	hgnc_symbol	log2FoldChange	pvalue	padj
ENSG00000144476	ACKR3	-0.341517497	0.156194067	0.276343348
ENSG00000129048	ACKR4	-0.290274593	0.500482137	0.628381546
ENSG00000196839	ADA	-0.028085509	0.941475147	0.96102668
ENSG00000160710	ADAR	0.309505183	3.67604E-08	2.6015E-07
ENSG00000164022	AIMP1	-0.372844463	0.000143086	0.000607565
ENSG00000164111	ANXA5	0.09302267	0.055659235	0.121920229
ENSG00000115307	AUP1	0.0478459	0.504625751	0.628381546
ENSG00000171791	BCL2	-0.511093889	0.012872659	0.034162056
ENSG00000113916	BCL6	0.067697497	0.523088909	0.638662144
ENSG00000125378	BMP4	0.18746568	0.683121391	0.775890963
ENSG00000153162	BMP6	-0.415526834	0.368945103	0.528448143
ENSG00000130303	BST2	-0.087772662	0.140774257	0.262524967
ENSG00000125730	C3	-0.201031458	0.011308328	0.031210985
ENSG00000171860	C3AR1	-0.503007458	0.049762765	0.113508455
ENSG00000106804	C5	-0.739278656	0.036006239	0.086414974
ENSG00000137752	CASP1	0.222154723	0.022907183	0.058003508
ENSG00000064012	CASP8	-0.12863198	0.351923849	0.508539174
ENSG00000105974	CAV1	0.247569739	0.001800648	0.006135542
ENSG00000114423	CBLB	-0.063989175	0.653896992	0.748861285
ENSG00000108691	CCL2	-0.180508787	7.0817E-05	0.00031525
ENSG00000115009	CCL20	0.073130035	0.823132081	0.890919429
ENSG00000006606	CCL26	-0.240567502	0.604178715	0.700644224
ENSG00000108688	CCL7	-1.531774457	0.000519505	0.001886624
ENSG00000163823	CCR1	0.221932302	0.626780229	0.723813152
ENSG00000184451	CCR10	-0.092881514	0.82902561	0.893793236
ENSG00000121797	CCRL2	0.346913718	0.446397329	0.603949327
ENSG00000170458	CD14	-0.093205555	0.505437331	0.628381546
ENSG00000120217	CD274	-0.047404844	0.678935635	0.774323286
ENSG00000103855	CD276	0.317437869	0.010724995	0.029899986
ENSG00000101017	CD40	-0.613555107	0.162725057	NA
ENSG00000026508	CD44	0.089068094	0.462731736	0.614762686
ENSG00000196776	CD47	-0.098419186	0.403222929	0.55093826
ENSG00000135404	CD63	-0.045189887	0.346312548	0.50572626
ENSG00000019582	CD74	-2.424089164	1.11995E-48	4.4158E-47
ENSG00000110651	CD81	0.383426872	2.94501E-10	2.4631E-09
ENSG00000010278	CD9	-0.304670974	6.78682E-08	4.56869E-07
ENSG00000237350	CDC42P6	0.652617435	0.154386799	0.274908106
ENSG00000123374	CDK2	0.308352564	0.000818614	0.002859967
ENSG00000124762	CDKN1A	-1.59675286	1.45101E-53	6.67465E-52
ENSG00000111276	CDKN1B	-0.018410503	0.866135649	0.912417706
ENSG00000172216	CEBPB	-0.554702924	0.000677716	0.002398072
ENSG00000213341	CHUK	-0.06316483	0.469981618	0.614762686
ENSG00000179583	CIITA	0.25282333	0.546996751	0.64794465
ENSG00000109846	CRYAB	0.030615081	0.905020908	0.932466766
ENSG00000184371	CSF1	0.505979757	3.08525E-11	2.93631E-10

ENSG00000182578	CSF1R	0.550816135	0.224005117	0.368008406
ENSG00000164400	CSF2	-1.081697316	3.92323E-33	9.02343E-32
ENSG00000108342	CSF3	-2.771484632	1.25004E-10	1.11294E-09
ENSG00000006210	CX3CL1	-0.888887787	1.66533E-05	8.62928E-05
ENSG00000163739	CXCL1	-3.299823848	1.1542E-244	1.0618E-242
ENSG00000169245	CXCL10	0.812265144	9.56004E-48	3.29821E-46
ENSG00000169248	CXCL11	0.404640528	5.69448E-14	6.54865E-13
ENSG00000107562	CXCL12	0.308509008	0.056720131	0.123265797
ENSG00000161921	CXCL16	-0.022588225	0.897331553	0.932466766
ENSG00000081041	CXCL2	-2.730114907	1.508E-127	8.3242E-126
ENSG00000163734	CXCL3	-1.79039284	3.99791E-07	2.50778E-06
ENSG00000163735	CXCL5	-0.126858012	0.375649707	0.528976118
ENSG00000124875	CXCL6	-3.260852056	0	0
ENSG00000169429	CXCL8	-2.485614084	0	0
ENSG00000138755	CXCL9	0.66726648	0.147749112	0.26652781
ENSG00000121966	CXCR4	0.106627866	0.731695172	0.812690052
ENSG00000107201	DDX58	0.579843847	1.89352E-24	4.02009E-23
ENSG00000149091	DGKZ	-0.000276549	0.993449055	0.993449055
ENSG00000179611	DGKZP1	0.038994094	0.928849581	NA
ENSG00000197635	DPP4	0.037940985	0.898958944	0.932466766
ENSG00000105246	EBI3	0.071660622	0.692073528	0.782837269
ENSG00000138798	EGF	-0.256800328	0.525376637	0.638662144
ENSG00000146648	EGFR	0.41815828	5.41779E-05	0.000245223
ENSG00000120738	EGR1	-1.071811251	1.74565E-11	1.72071E-10
ENSG00000122877	EGR2	-0.008939272	0.97812885	0.981685683
ENSG00000179388	EGR3	-0.605281313	0.188301979	0.318842615
ENSG00000055332	EIF2AK2	0.131280009	0.051646348	0.115889366
ENSG00000126767	ELK1	0.278588584	0.019509067	0.049856505
ENSG00000119888	EPCAM	-0.408646883	0.373360101	0.528448143
ENSG00000141736	ERBB2	0.351062504	0.07004315	0.146453859
ENSG00000117525	F3	0.521903259	1.28195E-12	1.41527E-11
ENSG00000026103	FAS	-1.105805903	2.22503E-20	3.83818E-19
ENSG00000112787	FBRSL1	-0.33915488	0.003832657	0.012020605
ENSG00000137312	FLOT1	-0.026250892	0.954252059	0.966989427
ENSG00000170345	FOS	-0.473304767	0.012161563	0.032907758
ENSG00000175592	FOSL1	-0.113795359	0.027878826	0.068093416
ENSG00000114861	FOXP1	-0.227828863	0.005582566	0.01693174
ENSG00000107485	GATA3	0.203590133	0.469831393	0.614762686
ENSG00000117228	GBP1	0.470711325	4.45505E-17	6.14797E-16
ENSG00000162676	GFI1	-0.365194533	0.345563938	0.50572626
ENSG00000105220	GPI	0.313927429	4.57724E-07	2.74635E-06
ENSG00000094631	HDAC6	0.706253062	0.000273394	0.001062773
ENSG00000048052	HDAC9	-0.104292937	0.334297535	0.498735781
ENSG00000100644	HIF1A	0.11766563	0.088287711	0.174052916
ENSG00000203812	HIST2H2AA3	0.794009785	0.085788116	0.170341872
ENSG00000272196	HIST2H2AA4	1.535130486	0.000846962	0.00292202
ENSG00000184260	HIST2H2AC	0.077504604	0.859192857	0.908571757
ENSG00000206503	HLA-A	0.865570541	0.060971807	0.129447836
ENSG00000234745	HLA-B	-0.713411886	0.002492741	0.008289113
ENSG00000204525	HLA-C	-0.288668951	0.220568371	0.368008406

ENSG00000204632	HLA-G	-0.696543302	0.099418513	0.193235984
ENSG00000189403	HMGB1	-0.154017773	0.054359624	0.120026049
ENSG00000117594	HSD11B1	-0.761025838	5.41979E-05	0.000245223
ENSG00000080824	HSP90AA1	0.207536643	0.003258945	0.010581985
ENSG00000106211	HSPB1	0.300767877	0.023198282	0.058206598
ENSG00000144381	HSPD1	-0.084009324	0.185939686	0.317958645
ENSG00000090339	ICAM1	0.099963075	0.042490264	0.099384008
ENSG00000115738	ID2	-1.109125843	8.52471E-06	4.52465E-05
ENSG00000131203	IDO1	0.474990353	0.000409694	0.00154898
ENSG00000163565	IFI16	-0.02630164	0.733187764	0.812690052
ENSG00000165949	IFI27	-0.210798653	0.562708078	0.660882679
ENSG00000137965	IFI44	-0.265220348	6.02966E-08	4.16047E-07
ENSG00000137959	IFI44L	-0.252843487	4.5769E-07	2.74635E-06
ENSG00000126709	IFI6	0.163034663	0.018633316	0.048063507
ENSG00000115267	IFIH1	0.301693671	0.002650303	0.008708138
ENSG00000185745	IFIT1	0.485003964	1.41157E-11	1.44294E-10
ENSG00000119922	IFIT2	0.801145151	1.07808E-35	2.70501E-34
ENSG00000119917	IFIT3	0.400254313	5.40933E-19	8.7822E-18
ENSG00000185885	IFITM1	-0.392406665	0.319731658	0.487546617
ENSG00000185201	IFITM2	-0.029674073	0.774879138	0.855466569
ENSG00000142089	IFITM3	0.085631583	0.247010473	0.389570803
ENSG00000142166	IFNAR1	-0.027762968	0.705672429	0.791730043
ENSG00000171855	IFNB1	0.24311933	0.549509507	0.648139418
ENSG00000184995	IFNE	0.103148132	0.799460124	0.873377623
ENSG00000027697	IFNGR1	-0.149681892	0.060815624	0.129447836
ENSG00000159128	IFNGR2	-0.25126264	0.487901094	0.623429176
ENSG00000185436	IFNLR1	0.083602971	0.84553657	0.901035109
ENSG00000104365	IKBKB	0.305353179	0.023730668	0.059005985
ENSG00000095752	IL11	-1.28816151	9.42079E-12	1.00005E-10
ENSG00000137070	IL11RA	0.24143019	0.597720097	0.696079101
ENSG00000168811	IL12A	-0.517539774	0.028992992	0.070193559
ENSG00000096996	IL12RB1	-0.109636579	0.804332071	NA
ENSG00000131724	IL13RA1	-0.056935644	0.495948778	0.627898453
ENSG00000164136	IL15	-0.237222538	0.152525883	0.273358076
ENSG00000177663	IL17RA	0.345155242	0.39847353	0.549893471
ENSG00000163701	IL17RE	-0.391534541	0.370291929	NA
ENSG00000115604	IL18R1	0.20146186	0.486780983	0.623429176
ENSG00000115008	IL1A	-1.31965023	0.000601269	0.002155198
ENSG00000125538	IL1B	-1.502812508	4.41956E-10	3.58765E-09
ENSG00000115594	IL1R1	0.085098775	0.574943113	0.672391099
ENSG00000196083	IL1RAP	0.016481603	0.905438744	0.932466766
ENSG00000136689	IL1RN	-0.826957082	0.057767929	0.124562098
ENSG00000174564	IL20RB	-0.677360648	0.076116006	0.157955019
ENSG00000103522	IL21R	0.179015722	0.695214365	NA
ENSG00000110944	IL23A	-0.0547379	0.901238692	0.932466766
ENSG00000104998	IL27RA	-0.159531544	0.453974392	0.608237535
ENSG00000164509	IL31RA	-0.392662784	0.393952732	0.546386704
ENSG00000077238	IL4R	-0.382375972	0.039373368	0.093681461
ENSG00000136244	IL6	-2.530939685	3.8328E-225	2.6446E-223
ENSG00000160712	IL6R	0.448057196	0.329348743	0.494023114

ENSG00000104432	IL7	0.042983489	0.882876638	0.926516928
ENSG00000168685	IL7R	1.275624585	8.59458E-47	2.63567E-45
ENSG00000123999	INHA	-1.253038397	0.003733808	0.011845185
ENSG00000122641	INHBA	1.180006494	2.07841E-10	1.79262E-09
ENSG00000184216	IRAK1	-0.2274527	0.511095996	0.632567242
ENSG00000134070	IRAK2	-0.302558453	0.054238536	0.120026049
ENSG00000198001	IRAK4	0.088943088	0.466260126	0.614762686
ENSG00000125347	IRF1	0.091090207	0.538390732	0.644896027
ENSG00000168310	IRF2	0.083099603	0.182746666	0.317220628
ENSG00000170604	IRF2BP1	0.278172639	0.069567472	0.146453859
ENSG00000126456	IRF3	0.077849169	0.315371117	0.483569046
ENSG00000128604	IRF5	-0.41385697	0.368331846	NA
ENSG00000117595	IRF6	0.01320464	0.974434057	NA
ENSG00000185507	IRF7	0.466147465	0.169929129	0.296838224
ENSG00000187608	ISG15	0.636753568	1.91088E-18	2.7758E-17
ENSG00000172183	ISG20	0.568327916	1.83426E-14	2.20112E-13
ENSG00000078747	ITCH	0.119598217	0.242906268	0.387526762
ENSG00000213949	ITGA1	-0.304359888	0.045466322	0.105451302
ENSG00000160255	ITGB2	0.435697442	0.345282396	0.50572626
ENSG00000162434	JAK1	-0.075934021	0.408236112	0.555040231
ENSG00000096968	JAK2	0.119732101	0.479571402	0.619272192
ENSG00000177606	JUN	0.137695379	0.097741311	0.191323418
ENSG00000100578	KIAA0586	0.169670574	0.244955693	0.38855041
ENSG00000049130	KITLG	0.252569087	0.103902733	0.20053954
ENSG00000116678	LEPR	-0.766112981	0.003550593	0.011394926
ENSG00000131981	LGALS3	-0.736029019	8.55747E-24	1.68704E-22
ENSG00000128342	LIF	0.467601102	0.084206469	0.170091752
ENSG00000123384	LRP1	-0.273461428	0.085593826	0.170341872
ENSG00000154589	LY96	-1.828160133	1.16548E-18	1.78707E-17
ENSG00000254087	LYN	0.142032577	0.223133387	0.368008406
ENSG00000178573	MAF	0.464687251	0.131163157	0.24795227
ENSG00000034152	MAP2K3	0.126429184	0.502653838	0.628381546
ENSG00000095015	MAP3K1	0.515774249	0.000189428	0.000762134
ENSG00000169967	MAP3K2	0.032222164	0.784170155	0.862274752
ENSG00000135341	MAP3K7	-0.263409084	0.000171802	0.000707723
ENSG00000100030	MAPK1	0.161896739	0.026261365	0.064715507
ENSG00000107643	MAPK8	-0.112034289	0.241287657	0.38718252
ENSG00000105976	MET	0.097687257	0.296397917	0.459583287
ENSG00000130731	METTL26	-0.158099065	0.076826505	0.158239667
ENSG00000158411	MITD1	-0.478501175	3.5086E-05	0.00016989
ENSG00000100985	MMP9	-0.326810867	0.477391041	0.619272192
ENSG00000196814	MVB12B	-0.142911048	0.522967493	0.638662144
ENSG00000157601	MX1	0.518183297	1.09378E-08	7.94427E-08
ENSG00000183486	MX2	0.395219541	2.35852E-15	2.95887E-14
ENSG00000136997	MYC	0.787938988	1.12862E-06	6.62763E-06
ENSG00000172936	MYD88	0.345964269	1.6488E-06	9.48063E-06
ENSG00000158092	NCK1	-0.659305858	7.37114E-06	3.98909E-05
ENSG00000184983	NDUFA6	-0.341795441	0.142759866	0.264441094
ENSG00000131196	NFATC1	0.272068804	0.527590467	0.638662144
ENSG00000072736	NFATC3	-0.095718739	0.448699372	0.604102569

ENSG00000109320	NFKB1	0.223079335	0.012812565	0.034162056
ENSG00000077150	NFKB2	-0.066550854	0.539749936	0.644896027
ENSG00000100906	NFKBIA	0.272607353	2.46699E-07	1.58346E-06
ENSG00000170322	NFRKB	0.327999401	0.23468926	0.381024916
ENSG00000123609	NMI	0.267878839	2.39029E-05	0.000119949
ENSG00000106100	NOD1	0.189762974	0.537951679	0.644896027
ENSG00000148400	NOTCH1	-0.158302362	0.389999611	0.543635822
ENSG00000177463	NR2C2	0.023155639	0.835900187	0.897698255
ENSG00000113580	NR3C1	-0.254132131	0.000495986	0.001825229
ENSG00000123358	NR4A1	-0.28831612	0.514397186	NA
ENSG00000119508	NR4A3	-1.645240955	6.52505E-06	3.60183E-05
ENSG00000089127	OAS1	0.850374165	9.80314E-37	2.70567E-35
ENSG00000111335	OAS2	0.36771571	5.15977E-05	0.000245223
ENSG00000197329	PELI1	-0.368259923	0.01560266	0.040625794
ENSG00000140464	PML	0.585119742	1.44326E-07	9.4843E-07
ENSG00000028277	POU2F2	-0.646912267	0.010482127	0.029521093
ENSG00000186951	PPARA	-0.534176003	0.007205785	0.021617354
ENSG00000132170	PPARG	-0.255918554	0.236800749	0.382204717
ENSG00000084072	PPIE	-0.107654144	0.135634626	0.25466093
ENSG00000100023	PPIL2	0.284800528	0.004411932	0.013681945
ENSG00000180228	PRKRA	-0.468902152	7.72498E-09	5.76242E-08
ENSG00000092010	PSME1	0.014453079	0.965670756	0.972719448
ENSG00000125384	PTGER2	0.980907245	0.002065194	0.006951139
ENSG00000073756	PTGS2	-0.593905416	0.146192745	0.26652781
ENSG00000111737	RAB35	0.509298412	2.87131E-05	0.000141514
ENSG00000136238	RAC1	-0.01827496	0.730487538	0.812690052
ENSG00000162924	REL	-0.173223353	0.279598128	0.4384607
ENSG00000173039	RELA	-0.252843862	0.000366779	0.001405986
ENSG00000104856	RELB	-0.147647246	0.147152566	0.26652781
ENSG00000132005	RFX1	-0.348503115	0.008208163	0.024317462
ENSG00000104312	RIPK2	-0.115666445	0.19936824	0.33552216
ENSG00000069667	RORA	-0.787399114	0.041606664	0.098149054
ENSG00000159216	RUNX1	-0.161978211	0.128019708	0.243678892
ENSG00000020633	RUNX3	0.341212563	0.35152097	0.508539174
ENSG00000170989	S1PR1	0.295329521	0.495818609	0.627898453
ENSG00000188404	SELL	0.373757797	0.404542463	NA
ENSG00000185187	SIGIRR	0.337571701	0.299498866	0.461797134
ENSG00000145147	SLIT2	0.415283778	0.009985316	0.028411827
ENSG00000185338	SOCS1	0.042165813	0.840963234	0.899635088
ENSG00000184557	SOCS3	-0.42316481	0.008282034	0.024317462
ENSG00000171150	SOCS5	0.124968838	0.40254296	0.55093826
ENSG00000118785	SPP1	0.012521372	0.96497241	NA
ENSG00000115415	STAT1	0.262989906	7.26904E-09	5.57293E-08
ENSG00000170581	STAT2	0.307397894	0.005301411	0.01625766
ENSG00000168610	STAT3	0.104688307	0.1866279	0.317958645
ENSG00000138378	STAT4	-0.727456491	0.080297692	0.164164169
ENSG00000126561	STAT5A	0.770019856	0.000484248	0.001806114
ENSG00000166888	STAT6	0.28908604	0.466052993	0.614762686
ENSG00000168394	TAP1	-0.204212352	0.643230975	NA
ENSG00000231925	TAPBP	0.358486969	0.370036973	0.528448143

ENSG00000183735	TBK1	0.096635534	0.291353856	0.454314488
ENSG00000092969	TGFB2	0.780166119	3.98404E-11	3.66531E-10
ENSG00000119699	TGFB3	-0.02280028	0.956478672	0.966989427
ENSG00000041988	THAP3	0.276750957	0.109611124	0.210087988
ENSG00000137801	THBS1	1.05477844	2.90642E-09	2.29192E-08
ENSG00000102265	TIMP1	-0.466046809	1.58732E-15	2.08619E-14
ENSG00000150455	TIRAP	-0.255571694	0.578111057	NA
ENSG00000174125	TLR1	-0.344334732	0.34166884	0.50572626
ENSG00000137462	TLR2	0.294938335	0.161129571	0.283259628
ENSG00000164342	TLR3	0.515751469	0.000190533	0.000762134
ENSG00000136869	TLR4	0.21638631	0.328118384	0.494023114
ENSG00000187554	TLR5	-0.479704613	0.221514462	0.368008406
ENSG00000174130	TLR6	-0.289945602	0.526549165	0.638662144
ENSG00000184584	TMEM173	0.168171801	0.226547865	0.369983496
ENSG00000104689	TNFRSF10A	0.204693681	0.144678293	0.266208059
ENSG00000164761	TNFRSF11B	-0.520945321	2.23135E-06	1.25684E-05
ENSG00000157873	TNFRSF14	0.087283651	0.822789603	0.890919429
ENSG00000186891	TNFRSF18	-0.903999237	0.050919374	0.11519465
ENSG00000067182	TNFRSF1A	0.356266814	8.81647E-05	0.000386245
ENSG00000120949	TNFRSF8	-0.314579696	0.480160323	0.619272192
ENSG00000049249	TNFRSF9	0.379590936	1.68834E-05	8.62928E-05
ENSG00000121858	TNFSF10	-0.198810179	0.009862929	0.028355921
ENSG00000239697	TNFSF12	-0.336570197	0.000121934	0.000525842
ENSG00000102524	TNFSF13B	0.499910189	0.185760824	0.317958645
ENSG00000117586	TNFSF4	-0.861485467	0.012031222	0.032877399
ENSG00000078902	TOLLIP	-0.066208236	0.697447529	0.785695992
ENSG00000141510	TP53	0.454275874	5.39185E-05	0.000245223
ENSG00000164938	TP53INP1	-0.551940637	0.000166886	0.000697889
ENSG00000131323	TRAF3	0.213374831	0.084429602	0.170091752
ENSG00000175104	TRAF6	-0.4180699	0.008727474	0.025355608
ENSG00000074319	TSG101	-0.174575615	0.013275737	0.034896222
ENSG00000084652	TXLNA	0.120030964	0.542702612	0.645628969
ENSG00000105397	TYK2	0.468997638	0.046942241	0.107967154
ENSG00000025708	TYMP	0.025263602	0.943616777	0.96102668
ENSG00000177889	UBE2N	0.023320569	0.800596154	0.873377623
ENSG00000162692	VCAM1	0.082105555	0.371767259	0.528448143
ENSG00000112715	VEGFA	-1.299028954	4.32642E-22	7.96062E-21
ENSG00000167987	VPS37C	0.189402009	0.382936309	0.5364996
ENSG00000160685	ZBTB7B	-0.024502522	0.936545826	0.960916907
ENSG00000083838	ZNF446	-0.113964133	0.6495157	0.746943055
ENSG00000125726		-0.305167047	0.000197967	0.000780554
ENSG00000105329		0.0590653	0.857240276	0.908571757
ENSG00000127666		0.148696122	0.322219696	0.488640857
