# Supplementary material for: Microglial derived extracellular vesicles activate autophagy and mediate multi‐target signaling to maintain cellular homeostasis
Source: J Extracell Vesicles. 2020 Nov 25;10(1):e12022. doi: 10.1002/jev2.12022 (PMC7890546; doi:10.1002/jev2.12022)
Supplement: Supplementary file 7 — Supplementary table 5: qPCR primer sequences. [file JEV2-10-e12022-s007.pdf]

Supplementary table 5: qPCR primer sequences

	Forward primer	Reverse primer
**EPCAM**	GCCAGTGTACTTCAGTTGGTGC	CCCTTCAGGTTTTGCTCTTCTCC
**VEGFA**	TTGCCTTGCTGCTCTACCTCCA	GATGGCAGTAGCTGCGCTGATA
**FAS**	GGACCCAGAATACCAAGTGCAG	GTTGCTGGTGAGTGTGCATTCC
**TRAIL**	TGGCAACTCCGTCAGCTCGTTA	AGCTGCTACTCTCTGAGGACCT
**IL6**	AGACAGCCACTCACCTCTTCAG	TTCTGCCAGTGCCTCTTTGCTG
**GAPDH**	CGGAGTCAACGGATTTGGTCG	AGCCTTCTCCATGGTGGTGAAGAC
**CASP8**	ATCAATCAGAAGGGAAGACAAGTT	AGTAAGCAACAAGGATGACAAGA
**IL1b**	GGTGCTGATGTACCAGTTGGG	CCAAAGAAGAAGATGGAAAAGC
**CASP1**	GCTGTACCCCAGATTTTGTAGCA	TCCAATAATGGACAAGTCAAGCC
**RPL13a**	GCCCTACGACAAGAAAAAGCG	TACTTCCAGCCAACCTCGTGA
**FADD**	TCTCCTCTCTGAGACTGCTAA	AGAGAGTGCTGTGTGTCAATC
